# VOC emissions from particle filtering half masks – methods, risks and need for further action

**DOI:** 10.17179/excli2021-3734

**Published:** 2021-06-01

**Authors:** Saskia Kerkeling, Christian Sandten, Thomas Schupp, Martin Kreyenschmidt

**Affiliations:** 1 University of Applied Sciences Muenster, Stegerwaldstraße 39, 48565 Steinfurt

**Keywords:** particle filtering half masks, VOC, emissions, filtering face piece, risk

## Abstract

Investigations into volatile organic compound (VOC) emissions from polymer fleeces used in particle filtering half masks were conducted and evaluated against the German hygienic guide value for total volatile organic compounds and the “Lowest Concentration of Interest” for construction products. All masks showed emission of Xylene. In 94 % of samples, up to 24 additional aromatic compounds were found. 17 % of samples showed terpenes, 53 % emitted aldehydes, 77 % exhibited caprolactam and 98 % released siloxanes. All masks exceeded the TVOC hygienic guidance value level 5 of 10 mg/m³. Emission levels were investigated for masks immediately after their packages were opened and for masks that were “vented” for two weeks. Further, the emissions were repeatedly measured to investigate the decrease of emissions. An exponential decline was observed and a fitting function was calculated. The influence of the two commonly gas chromatograph (GC) hyphenated detectors, mass spectrometer (MS) and flame ionization detector (FID) on the VOC quantification, as well as the influence of temperature on the emission of VOCs were investigated. A statistical analysis of emission value differences for Notified Bodies was conducted and CE 2163 and 2020-1XG proved to be outliers.

## Introduction

One effect of the SARS-CoV-2 pandemic has been the expansion of mask-wearing. Almost overnight, the entire global population began wearing masks on a daily basis. This rapid market expansion put a spotlight on every material that could cover a human's face and nose, particle filtering half masks in particular.

While testing other chemical characteristics of particle filtering half masks, strong odors being emitted from some masks upon opening their packaging were noticed. Surprisingly, literature searches revealed that there is no established testing method for volatile compounds emitted from materials used in manufacturing these masks.

It should be emphasized that within the last three decades there have been no reports of detrimental side-effects attributed to wearing particle filtering half masks while there have been numerous reports supporting the benefits. Therefore, one can assume that the benefits of mask-wearing and the protection they provide outweigh most possible risks.

Here, we report on emissions of volatile organic compounds (VOC) for a variety of filtering face pieces (FFP) which were available in local shops. We describe the method of testing, the influence of the temperature on the emissions, and detector selection for the quantification of VOCs, and we provide an initial, tentative toxicological evaluation.

This work is not a call to arms against the use of masks. Rather, we aim to draw attention to a situation where the lack of regulation can potentially lead to health issues.

## Materials and Methods

### Sample selection

Initially, random samples available on the German market were examined. During our investigations, complaints from the public of “bad smell[s]” coming from masks certified by CE 2163 were reported. This led to the higher abundancy of those particular masks in our analysis via public donations or our direct acquisition. A total of 47 masks were investigated: 31 FFP2 masks and 16 KN95 masks. These masks were introduced into our study as duplicate or triplicate measurements, if possible, from separate masks of the same type and brand, depending on the number of available samples.

### Sample preparation

Fleece samples were prepared using a punching die to create discs 40 mm in diameter for a reproducible surface area of 12,56 cm². The discs were weighed, and result calculations were extrapolated to the weights of the entire mask's fleece material.

### Sample collection 

In accordance with DIN ISO 16000-6 (VDI/DIN-Kommission Reinhaltung der Luft (KRdL), 2020[10]), volatile organic compounds in filter fleeces were investigated using a Markes Micro-Chamber/Thermal Extractor set to 40 °C. A nitrogen 5.0 flow of 58 mL/ min was introduced into the microchamber, and emissions were collected for 17 minutes using Tenax TA thermodesorption tubes. The Perkin Elmer TurboMatrix 350 in connection with a Shimadzu GC-MS 2020 was used as the sample introduction system.

To investigate the emission value decline, three masks were sampled 10 times with one liter of nitrogen successively. Sample A was vented for two weeks at 20 °C prior to examination while Samples B and T were opened immediately before testing. Sample A was selected due to the low initial emission. Sample B was chosen to represent masks that emit a lower proportion of aromatic compounds while Sample T was chosen to represent masks that emit a higher proportion of aromatic compounds. 

Comparative measurements were made at 30 °C and 40 °C to examine the influence of the temperature on emissions.

Masks were either investigated immediately after opening their packaging or after being vented for at least seven days at 20 °C.

### TD-GC-MS method

#### Thermal Desorption Unit (TD)

The thermal desorption unit was set to -30 °C cryo focusing, 250 °C valve temperature, 280 °C Tube desorption and 200 °C Transfer line temperature. Column flow was set to 1 mL/min. Inlet and Outlet Split were set so that 5 % of tube adsorbate was injected into the GC system.

Thermal desorption took place at 280 °C over 10 minutes, and the system's cold trap was set to -30 °C.

#### Gas Chromatograph (GC)

The GC temperature program started at 40 °C with a three-minute hold time followed by a ramp of 10 °C/min up to 280 °C with a hold time of three minutes. As a stationary phase, a Shimadzu SH-Rtx-200MS crossbond trifluoropropylmethyl polysiloxane GC column with an inner diameter of 0.25 mm and a length of 30 m was used.

#### Mass Spectrometer (MS)

The Ion Source temperature was set to 200 °C. The interface temperature was set to 250 °C. The MS detector's event time was set to 0.07 seconds.

Qualitative analysis of VOC was based on mass spectra comparison with NIST 05, NIST 05s, NIST 08, and NIST 08s database on Shimadzu's LabSolutions GCMS solution Version 4.45.

Due to the limited number of substances available in the data bank and the fact, that similar molecular structures lead to similar mass spectra, different substances may be assigned to one individual compound.

All compounds with a similarity index above 80 were listed as identified.

### Toluene Equivalent Calculation

VOC emissions were quantified as Toluene Equivalents. The peak area was calculated by integrating the Total Ion Current signal.

The minimum peak width was set to 5 seconds. The sum of all volatile compounds was calculated between 6.75 minutes and 26 minutes.

### Calibrations

Toluene and Xylene were calibrated by injecting known concentrations of n-pentane solutions into thermodesorption tubes subjected to a nitrogen flow of 50 mL/min and followed by five minutes wait period to allow for solvent evaporation. 

Calibration of Toluene was conducted with seven masses equidistantly from 4.5 to 45 ng. Xylene was calibrated using an isomer mixture with seven masses equidistantly from 4.3 to 152 ng. Xylene was calibrated in addition to Toluene due to its detection in all masks. 

Peak area calculation for Xylene was done by integrating the peaks for all three isomers. Ions with a mass to charge ratio of 91 were used as target ions. Additionally, two reference ions with a mass to charge ratio of 106 and 105 were selected. The calibration was performed as group calibration of all isomers.

### GC-FID method

The flame ionization detector temperature was set to 300 °C. The sampling rate was set to 40 msec. Hydrogen flow was set to 45 mL/min, and airflow was set to 450 mL/min. Helium was used as makeup gas at 30 mL/min. A temperature program starting at 40 °C held for one minute followed by a ramp of 20 °C/min up to 100 °C and 45 °C/min to 250 °C with a two minutes hold time was used.

### MS FID comparison

To compare Toluene Equivalents calculated on a GC-MS with those from GC-FID, a solution with equimolar concentrations of Toluene, decane, dodecane, tetradecane, hexadecane, and octadecane was measured using either system. Integrated peak areas on each system were averaged and divided by the Toluene peak area.

### Toxicological evaluation

For a first orientation, the hygienic values for total volatile organic compounds (TVOC) issued by the German Indoor Air Hygiene Commission were used as a benchmark (Ausschuss fuer Innenraumrichtwerte, 2021[3]). According to that scheme, a TVOC below 0.3 mg/m³ is regarded as hygienically non-critical, whereas levels above 10 mg/m³ are regarded as hygienically unacceptable. For the toxicological evaluation of individual substances or substance classes, the ”Lowest Concentration of Interest” (LCI) for construction products was taken as a reference (EU-LCI, 2021[6]). The LCI concept is in most parts identical to the German AgBB scheme (AgBB, 2020[1]), which is one of the predecessors of the LCI concept, and where not only individual compounds are addressed, but also the TVOC, Total Semi Volatile Organic Compounds (TSVOC) and potential interaction of the different VOC.

## Results

### VOC emission behavior 

**Figure 1:**
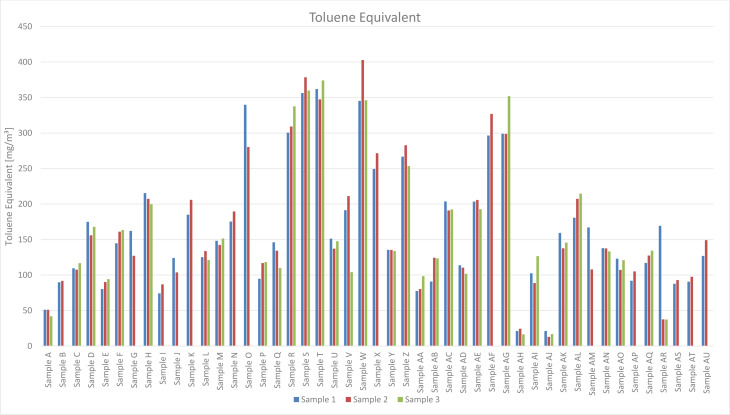
Toluene Equivalents in mg/m³ for Samples A to AU. Samples AH and AJ were measured after venting. The lowest emissions from a newly opened package were observed from Sample A. Toluene Equivalents for freshly unwrapped masks range from 50 mg/m³ (Sample A) to 403 mg/m³ (Sample W) with an arithmetic average of 172 mg/m³ and a median of 144 mg/m³. The lowest emission was determined from a vented mask as 13 mg/m³ TVOC (Sample AJ).

Concerning the TVOC, Toluene Equivalents for freshly unwrapped masks range from 50 mg/m³ (Sample A) to 403 mg/m³ (Sample W) with an arithmetic average of 172 mg/m³ and a median of 144 mg/m³. The lowest emission was determined from a vented mask as 13 mg/m³ TVOC (Sample AJ), as can be seen in Figure 1(Fig. 1).

The emission levels of all masks exceeded the applied TVOC hygienic guidance value of 10 mg/m³. For Samples AH and AJ, the guidance value was exceeded by a factor of approximately two and for Sample W by a factor of 40.

Vented masks showed lower emission values, as seen in Figure 1(Fig. 1). For Sample AR, the first measurement was performed immediately after the original packaging was opened, the second and the third test were performed after the mask was vented. Samples AH and AJ were also vented. 

**Figure 2:**
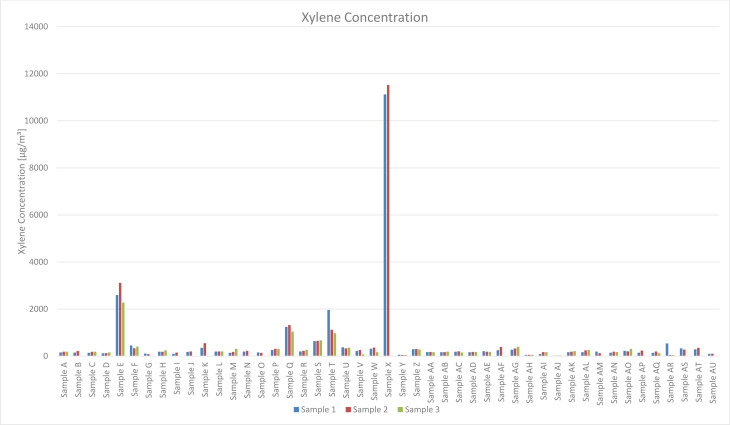
Xylene Isomer Concentrations in µg/m³ for Sample A to AU. Xylene Concentration ranges from 50 µg/m³ (Sample Y) to 12000 µg/m³ (Sample X) with an arithmetic average of 529 µg/m³ and a median of 198 µg/m³.

For aromatic compounds, Xylene isomers were always identified. Xylene Concentration ranges from 50 µg/m³ (Sample Y) to 12000 µg/m³ (Sample X) with an arithmetic average of 529 µg/m³ and a median of 198 µg/m³. The lowest Xylene Concentration of a vented mask was found at Sample AJ (25 µg/m³), as shown in Figure 2(Fig. 2).

### Exponential decline of VOC levels

**Figure 3:**
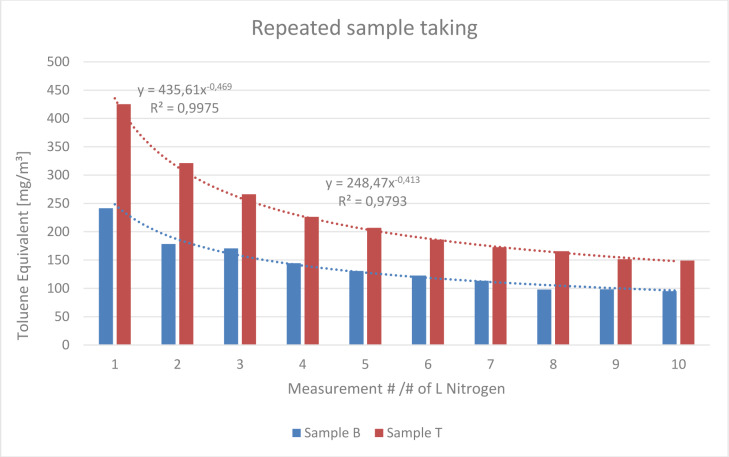
Toluene Equivalent calculated for the repeated sampling of Sample B and T. Both opened immediately before the examination. Sample B was chosen to represent masks that emit a lower proportion of aromatic compounds while Sample T was chosen to represent masks that emit a higher proportion of aromatic compounds. Both show an exponential decline of the Toluene Equivalent. The measurement # equals the liter of nitrogen that was sampled. The first measurement equals the first liter of nitrogen, the second measurement equals the second liter of nitrogen, and so on.

When a freshly opened sample was sampled ten times with one liter of nitrogen, an exponential decline in VOC concentration was observed. This decline was observed both in a sample containing mainly alkanes and, in a sample, containing a higher proportion of aromatic compounds, as can be seen in Figure 3(Fig. 3). 

**Figure 4:**
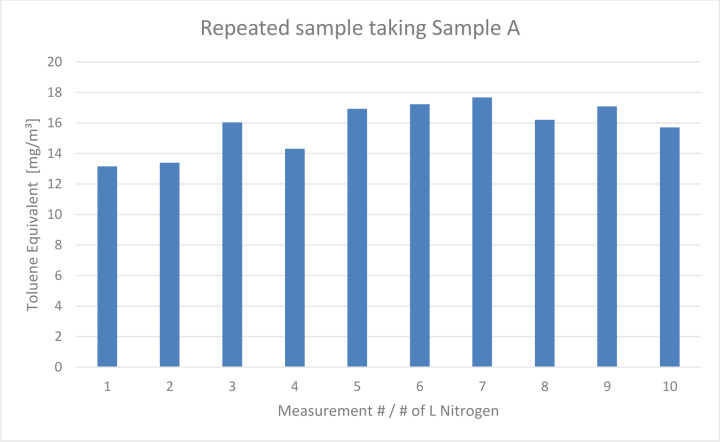
Toluene Equivalent calculated from the repeated sampling of vented Sample A. It showed continuous but steady emission of VOCs. The measurement # equals the liter of nitrogen that was sampled. The first measurement equals the first liter of nitrogen, the second measurement equals the second liter of nitrogen, and so on.

When ten repetitive measurements of a vented sample were conducted, no trend in emissions was observed, as shown in Figure 4(Fig. 4). The averaged sample emissions were 15.77 mg/m³ with a standard deviation of 1.62 mg/m³.

### VOC concentration dependence on sampling temperature and detection mode

**Figure 5:**
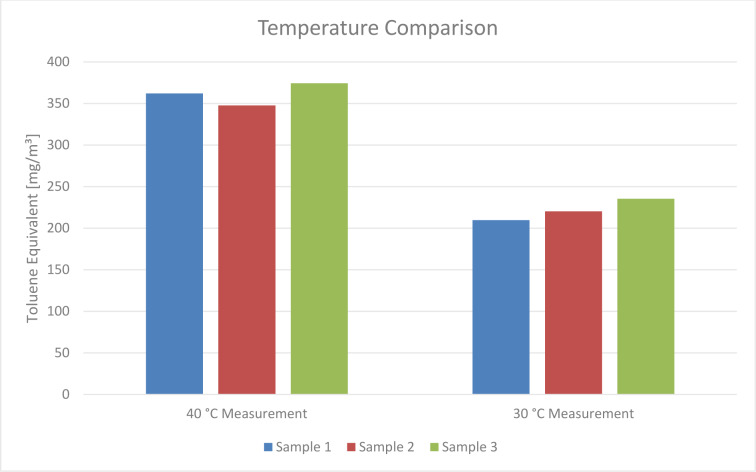
Toluene Equivalents measured at 40 °C and 30 °C. Sample taking duration and nitrogen flow rate were kept constant. Emissions were lowered by 40 %.

The emission level comparison at 30 °C and 40 °C revealed lower results at 30 °C, as can be seen in Figure 5(Fig. 5). The Toluene Equivalent decreased by 40 % when the sampling temperature was reduced. Nevertheless, the hygienic guidance value of TVOC for this sample is exceeded by a factor of 22.

**Figure 6:**
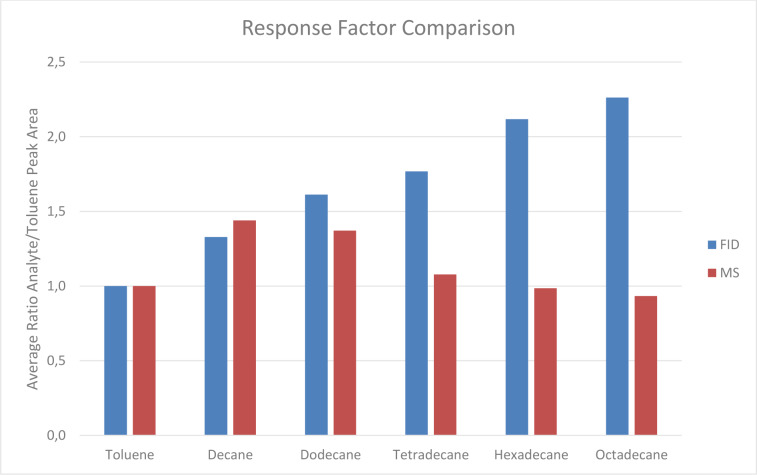
Ratio of Analyte to Toluene Response for hydrocarbons with increasing carbon numbers. The ratio of Analyte to Toluene Response grows with increased carbon number for FID measurements but for MS only grows for decane and dodecane and remains at a steady level for higher hydrocarbons.

Measurements conducted to evaluate the difference in detector response factors between an MS and an FID showed that the response for analytes with higher numbers of carbon atoms per molecule increased linearly on the FID. The MS detection led to an increased response factor for decane and dodecane then slowly declined to a constant level, as shown in Figure 6(Fig. 6).

### Differences in VOC levels in dependence on Notified Bodies

**Figure 7:**
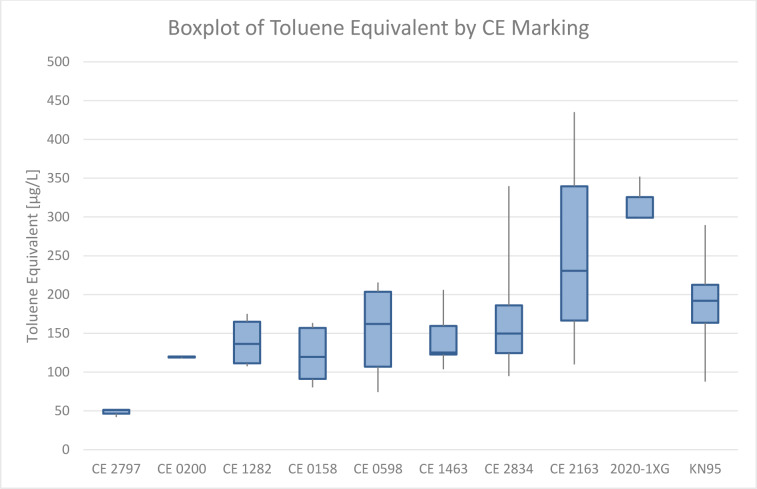
Boxplot of Toluene Equivalents measured per CE marking. There was a total of one mask with CE 2797, one mask for CE 0200, two masks for CE 1282, two masks for CE 0158, three masks for CE 0598, three masks for CE 1463, four masks for CE 2834, sixteen masks for CE 2163, one mask with a non-recognized certificate called 2020-1XG, and fourteen KN95 masks. Based on a boxplot of Toluene Equivalents across the respective Notified Body an uneven statistical distribution of emission values was suspected.

In the course of the investigations, deviations between different Notified Bodies were observed. Based on a boxplot of Toluene Equivalents across the respective Notified Body an uneven statistical distribution of emission values was suspected. All individual values from each mask measurement series were used to create the boxplot seen in Figure 7(Fig. 7). Due to different numbers of samples in the respective classes, the representation in the boxplot diagram may be misleading, therefore further statistical analysis was conducted.

Using a Kruskal-Wallis-Test after removing vented mask measurements from the dataset, significant stochastic dominance of at least one class was identified (df 9; 99.5 % probability). After removing CE 2163 and 2020-1XG no stochastic dominance was observed (df 7; 99.5 % probability).

### Risk evaluation of VOC

Samples B and T were taken from the dataset for further in-depth evaluation. Sample B was selected as a sample with relatively low emissions (TVOC: 91 mg/m³), while Sample T was one of the samples with the highest VOC emissions (TVOC: 361 mg/m³). Apart from the Xylene Concentration, all other concentrations are given as Toluene Equivalents. The identified VOCs, matched against their respective LCI-values, are available in the Supplementary material.

With a TVOC of about 90 mg/m³ Toluene Equivalents, Sample B exceeded the threshold of level 5 (“non-acceptable") ninefold. 87 out of 102 compounds emitted from Sample B were identified via the mass spectra database and matched against the 45 available LCI values (Supplementary material). Several peaks were assigned to a single compound, for example, “eicosane”, which were assumed to be high molecular alkanes.

The main emissions from Sample B were alkanes, with a total of 62 different hydrocarbons identified. The sum of C9-C16 alkanes added up to approximately 63 mg/m³ and exceeded the LCI for this class of compounds tenfold. 

A total amount of 1 mg/m³ of dimethylsiloxanes were found; for these, LCI are not available, but they are below the LCI for octamethylcyclotetrasiloxane of 1.4 mg/m³. 

Further, the measured Xylene Concentration was 0.18 mg/m³, which is below the LCI of 0.5 mg/m³. The caprolactam Toluene Equivalent was above its LCI, whereas decanal was approximately tenfold below its LCI. Additionally, seven alkenes were emitted from the mask fleece, 11 mg/m³ in total. The sum of the emitted alcohols was 9 mg/m³. For these compounds, LCI have not been published.

With a TVOC of about 361 mg/m³, Sample T exceeded the hygienic TVOC level 5 (“non-acceptable”) 36-fold. Details are listed in the Supplementary material. LCI are available in 40 out of the 87 identified compounds. The main emissions from Sample T were again alkanes, 181 mg/m³ in total, and the LCI for C9-C16 alkanes was exceeded more than 20-fold (162 mg/m³ against 6 mg/m³). The second-largest proportion consisted of alkylated benzenes with 93 mg/m³, and the LCI for tetramethyl benzene was exceeded by more than 100 times. Furthermore, six alcohols, five alkenes, and four dimethylsiloxanes were found. The Xylene Concentration was 1.5 mg/m³ and three times higher than the LCI. 

## Discussion

### Qualitative

Our results show a large variety of VOCs emitted from filter materials. Emissions that were qualified using the mass spectral database were, in most cases, aromatic compounds such as Toluene and other alkylated benzenes and a variety of different alkanes. Xylene was found in every sample. To classify VOC compounds, the qualitative results were searched for substrings as shown in Table 1(Tab. 1).

Alkanes and alcohols were found in every sample. In 83 % of samples, at least one aromatic compound was found in addition to Xylene and Toluene. In 98 % of samples, siloxanes were found. 17 % of the samples contained at least one compound from the class of terpenes. In addition, caprolactam was found in 75 % of the samples and aldehydes in 55 % of the samples. Esters were found in 98 % of the samples. Phthalates were only found in Sample A.

Qualitative analysis of VOC was based on mass spectra comparison with the NIST mass spectral library. In the case of potentially harmful substances, the comparison results were evaluated and confirmed by the analyst. In general, qualification of VOC can be challenging; therefore, qualitative results are considered as indications of substance classes such as alkanes, alkenes, aromatic compounds, and esters rather than a safe identification of a particular substance.

### VOC emission behavior

#### Exponential decline of VOC levels

Our repetitive measurements of Sample A indicated that emissions reach a constant level after an initial decrease. Even after two weeks of storage in ambient air at 20 °C, VOC levels stabilized at about 16 mg/m³.

**Figure 8:**
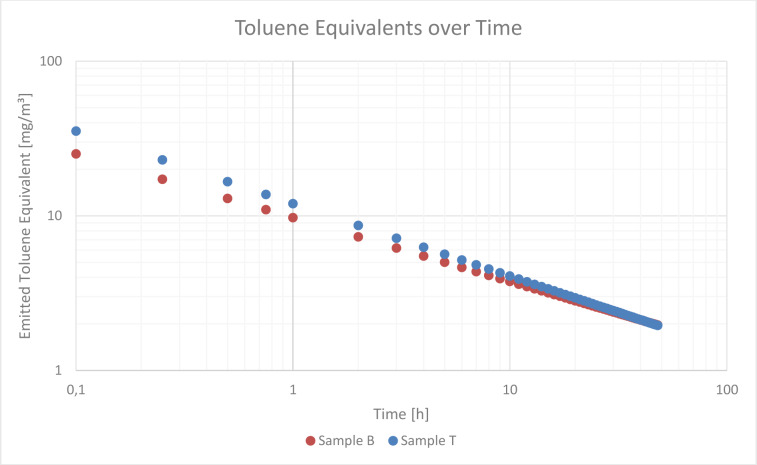
Extrapolated Emitted Toluene Equivalent over Time for Sample B and Sample T. Sample B was chosen to represent masks that emit a lower proportion of aromatic compounds while Sample T was chosen to represent masks that emit a higher proportion of aromatic compounds. Based on the before mentioned assumptions the TVOC reach level 4 after one hour.

An exponential decline was assumed in order to calculate the time needed for emissions to drop below the hygienic guidance level values. It can also be assumed that at a breathing volume of 480 L/hour, half of the measured emissions are inhaled while the other half is exhaled. Based on these assumptions, TVOC levels are at level 5 for one hour before they reach level 4, as shown in Figure 8(Fig. 8). After 18 hours, TVOC levels reach level 3. It would then take 247 hours for emissions to reach level 2, but emissions seem to stabilize at 16 mg/m³. Therefore, it is unknown if level 3 can be reached. 

The experimental setup could be considered a best-case scenario when it comes to emissions decline because of the continuous removal of VOC from the gas phase around the fleece. 

#### Detector dependence

TVOC emission values are often determined as Toluene Equivalent (as proposed in the German Indoor Guide Values) (Ausschuss fuer Innenraumrichtwerte, 2021[3]). The TVOC values in Toluene Equivalents are semi-quantitative because individual compounds in the mixture may have response factors that differ greatly from those of Toluene.

Further, the response factors of individual compounds may be detector-dependent. This detector dependence is not addressed in the formulation of limits. Therefore, whenever regulated values are given without a specified quantification method, they should be defined for different detector types.

In this project, the researching group used an MS instead of an FID to quantify TVOC. TVOC limits are commonly defined as Toluene Equivalents measured with GC-FID. Our comparison of response factors measured with MS and FID for n-Alkanes and Toluene shows that Toluene Equivalents calculated by our group will always be below levels calculated from GC-FID measurements. TVOC measured close to regulated limits by MS are likely to exceed limits when measured with FID by a factor of up to two and potentially higher.

### Toxicological evaluation

The German hygienic Indoor Guide Value for TVOC was used for an initial, tentative orientation for sample evaluation. The TVOC is split up into five levels, with level 1 (< 0.3 mg/m³) indicating a generally acceptable value, whereas level 5 (> 10 mg/m³) is rated as unacceptable from a hygienic perspective (Ausschuss fuer Innenraumrichtwerte, 2021[3]). It has to be noted that - on the one side - an exceedance of the respective TVOC thresholds does not necessarily define a toxicological risk; on the other side, a TVOC threshold may not be violated while an individual compound exceeds its Lowest Concentration of Interest (LCI). For individual VOCs, the Lowest Concentrations of Interest (LCI) for construction products were used as the benchmark. It should be noted that the LCI is not meant as indoor air guidance values (EU-LCI, 2021[6]). Nevertheless, we regard these values as an acceptable benchmark for the following reasons: 1) if Indoor Guide Values are published by the German Panel for Indoor Air or by the French ANSES (ANSES, 2018[2]), then these values are taken up as LCI; 2) in the case of available toxicological data, LCI are derived by an independent expert panel based on the REACh Guidance Document R.8 (EChA, 2012[5]) for the calculation of “Derived No Effect Levels” (DNELs); 3) in case of lacking or incomplete toxicological data, the LCI working group performs a read-across or extrapolation from/to similar substances with a satisfactory data set. Most of the LCI are based on No Observed Adverse Effect Concentrations (NOAEC) from animal experiments and extrapolated to human beings by inter- and intra-species extrapolation factors. With the NOAEC as the Point of Departure, exceeding the LCI slightly does not necessarily mean a toxicological risk. However, in absence of substance specific data, the default factor between the NOAEC and the LOAEC is 3 (EU-LCI, 2021[6]); exceeding the LCI by this factor results in a concentration that should be regarded as harmful. This is the case for sample B, where 46.17 mg/m³ total tetramethyl benzenes (LCI: 0.25 mg/m³) and total 63.2 mg/m³ C9-C16 alkanes were analyzed (LCI: 6 mg/m³). For both substance groups, the LCI is exceeded more than tenfold. The LCIs address lifetime exposure at 24 hours per day and 7 days per week. Therefore, their applicability for the evaluation of the exposure against VOC from particle filtering face masks may be questionable as filtering face masks are typically worn only a few hours per day. Additionally, emission behaviors measured at 40 °C in this study are certainly the worst-case scenario: exhaled air's temperature is at approximately 36 °C while inhaled air will have an ambient temperature. Finally, the emission rate declines rapidly over the first few hours. Taking these points into account, the measurements shown here do not necessarily indicate a risk for users of the masks. To address these points, some implications according to the practical use of the filtering face masks need to be borne in mind. First, if the toxic effects are triggered by the VOC concentration rather than by the total daily dose (p. e. irritation or narcosis), then the LCI should be adhered to throughout. Second, as the user might already have been exposed to the individual VOCs at LCI level in indoor air, emissions from filtering face pieces worn outdoors should not exceed the respective LCI. Concerning the temperature used for VOC emission tests, samples taken at 30 °C were up to a factor of about two lower when compared to 40 °C. Nevertheless, in our opinion, situations of carrying a filtering face piece at elevated temperature should not be excluded *a priori*. Additionally, the measurements at 40 °C can be regarded as a safeguard when using a mass spectrometer. Unfortunately, the data present is too limited for this approach. Therefore, uniform regulation on how to address detector differences is needed. Concerning declining emissions, following producer instructions, the consumer shall use a fresh mask each day for up to several hours per day. Given this exposure scenario, the VOC emissions measured from freshly unpacked masks are regarded as more relevant for exposure estimation than those from vented masks. 

LCI data are not available for most of the detected VOCs. For several of these compounds, specific toxicological data may not be available or only available at a limited level. This is a general problem of trace impurities and was addressed with the “Threshold of Toxicological Concern” (TTC); see, for example Hartung (2017[7]) and literature cited therein. Depending on the Cramer Class, the TTC for repeated inhalation exposure is 0.07-23 µg per kg body weight per day, which is 0.21-69 µg/m³ for a 60 kg person, inhaling 20 m³ air per day. At this point, some responsibility needs to be addressed to the producers of filtering face pieces. First, post-production ventilation of the masks will result in significantly reduced exposure of users. For the remaining VOCs exceeding the TTC, tentative LCI needs to be derived. Currently, due to the lack of emission regulations for filtering face pieces, masks with high VOC emission rates might remain on the market because they pass the established tests such as those for filtering efficiency. Such a regulation concerning emissions needs to find the balance between acceptable consumer protection and avoidance of barriers to trade. In that respect, the TVOC threshold is to be regarded as a hygienic benchmark; its exceedance may probably not justify market restriction, but information of the customer / consumer may be required. Concerning individual LCI, producers may be requested to ensure these values are met for materials placed on the market. How to address a potential combinatorial action of the VOCs emitted is subject to ongoing debate; (see, p. e. SCHER, 2011[9]; OECD, 2018[8]; Bopp et al., 2019[4]). On the concepts currently discussed, the Hazard Index (HI) in general is regarded as conservative, but other methods, like an organ-specific HI might address the risk in a more appropriate way (OECD, 2018[8]). For construction products placed on the German market, the AgBB scheme sets limits for TVOC as well as for Total Semi-Volatile Organic Compounds (TSVOC) and the total concentration of substances without an LCI value; further, the HI shall not exceed a level of 1 (AgBB, 2020[1]). It is beyond of the scope of this paper to conclude on the best way for how to address mixture toxicity, and the reader is referred to the literature cited. 

Masks with high emission levels of VOC seem to concentrate on CE 2163, indicating different modes of operation in the associated laboratories. Application of a Kruskal-Wallis test showed significant stochastic dominance of at least one Notified Body within our dataset. After the removal of CE 2163 and the not-recognized 2020-1XG, no stochastic dominance was observed. Hence, a significant difference between CE 2163, the non-recognized certificate, and the rest of the dataset was statistically shown. Issuing standards for VOC emission tests for filtering face pieces and respiratory filters may help to remove such discrepancies between Notified Bodies. 

## Acknowledgements

Thanks to Mr. Triphaus for the provision of masks. Thanks to Sierra Pitman for proofreading and editing this manuscript. Furthermore, we want to thank Johanna Strotbaum and Colin Machill for their support during sample preparation.

## Supplementary Material

Supplementary information

## Figures and Tables

**Table 1:**
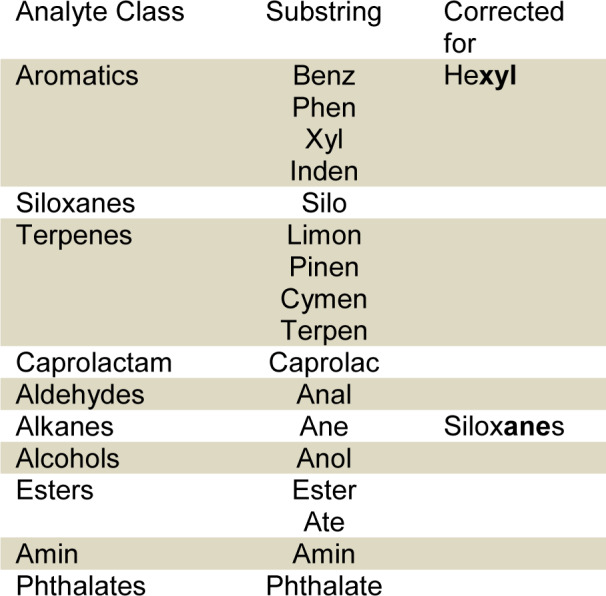
List of substrings and corrections for qualitative results' classification
