# Supplementary material for: VOC emissions from particle filtering half masks – methods, risks and need for further action
Source: EXCLI J. 2021 Jun 1;20:995–1008. doi: 10.17179/excli2021-3734 (PMC8278222; doi:10.17179/excli2021-3734)
Supplement: Supplementary information [file EXCLI-20-995-s-001.pdf]

EXCLI Journal 2021;20:995-1008 – ISSN 1611-2156
Received: April 08, 2021, accepted: May 26, 2021, published: June 01, 2021

Supplementary material to:

Original article:

VOC EMISSIONS FROM PARTICLE FILTERING HALF MASKS – METHODS, RISKS AND NEED FOR FURTHER ACTION

Saskia Kerkeling*, Christian Sandten, Thomas Schupp, Martin Kreyenschmidt

University of Applied Sciences Muenster, Stegerwaldstraße 39, 48565 Steinfurt

* **Corresponding author:** Saskia Kerkeling, University of Applied Sciences Muenster, Stegerwaldstraße 39, 48565 Steinfurt, E-mail: saskia.kerkeling@fh-muenster.de

http://dx.doi.org/10.17179/excli2021-3734

This is an Open Access article distributed under the terms of the Creative Commons Attribution License (http://creativecommons.org/licenses/by/4.0/).

EXCLI Journal 2021;20:995-1008 – ISSN 1611-2156
Received: April 08, 2021, accepted: May 26, 2021, published: June 01, 2021

Table S1 Hygienic Guidance Values for TVOC in indoor air (Ausschuss fuer Innenraumrichtwerte, 2021)

**Level**	**Concentration [mg TVOC/m³]**	**Hygienic evaluation**
**1**	≤ 0.3	'hygienically harmless'
**2**	0.3–1.0	'hygienically still harmless'
**3**	1.0–3.0	'hygienically conspicuous'
**4**	3.0–10	'hygienically critical'
**5**	≥ 10	'hygienically unacceptable'

Compound identification was conducted using NIST 05, NIST 05s, NIST 08 and NIST 08s database on Shimadzu's LabSolutions GCMS solution Version 4.45.
All compounds with a similarity index above 80 are listed as identified.
Database identification is not necessarily correct, due to different compounds having similar mass spectra and overlapping retention times can lead to overlapping mass spectra. Overlapping mass spectra of different compounds can in sum lead to high similarity indices with non-related compounds. Further, similar compounds can produce highly similar mass spectra. Hence, similar compounds can be identified by the same database mass spectra and hits might occur as duplicates in a table.
Due to the high reproducibility of the triplicate measurements, one random measurement for each sample was chosen for qualitative analysis.
Peak area gives the percentage of a peak's ratio to the sum of all peak ratios. Therefore, it also gives the ratio to the compound's Toluene Equivalent to the whole Toluene Equivalent.

Reference: Ausschuss fuer Innenraumrichtwerte. (vormals Ad-hoc-Arbeitsgruppe). Umweltbundesamt. [Online] 03 15, 2021. https://www.umweltbundesamt.de/themen/gesundheit/kommissionen-arbeitsgruppen/ausschuss-fuer-innenraumrichtwerte-vormals-ad-hoc#ausschuss-fur-innenraumrichtwerte.

EXCLI Journal 2021;20:995-1008 – ISSN 1611-2156
Received: April 08, 2021, accepted: May 26, 2021, published: June 01, 2021

To assess the VOCs, the substances identified by means of the database were searched qualitatively with regard to specific compound classes. The substrings ‘phen’, ‘benz’, ‘xyl’ and ‘inden’ were summarized as aromatic compounds and values were corrected for ‘hexyl’. Siloxanes were searched by the fragment ‘silo’. The substrings ‘limon’, ‘pinen’, ‘cymen’ and ‘terpen’ were recorded as terpenes. Caprolactam was searched using the word fragment ‘caprolac’ and aldehydes using the fragment ‘anal’. ‘Anol’ for alcohols and ‘ate’ and ‘ester’ for esters. ‘Phthal’ was used to search for phthalates. ‘Ane’ was used to look for alkanes and values were corrected for siloxanes. The number of respective hits for the samples are listed in the table below according to the substance classes.

Table S2 Number of hits for the respective compound class for Sample A to Sample AU

	Aroma-tics	Silox-anes	Ter-penes	Capro-lactam	Phtha-lates	Alde-hydes	Al-kanes	Alco-hols	Esters
Sample A	**3**	**1**	**0**	**0**	**1**	**0**	**42**	**6**	**2**
Sample B	**1**	**4**	**0**	**1**	**0**	**1**	**64**	**5**	**2**
Sample C	**1**	**3**	**0**	**1**	**0**	**1**	**50**	**6**	**1**
Sample D	**0**	**4**	**1**	**1**	**0**	**0**	**60**	**9**	**2**
Sample E	**2**	**0**	**2**	**0**	**0**	**0**	**55**	**8**	**4**
Sample F	**1**	**6**	**0**	**0**	**0**	**1**	**55**	**7**	**3**
Sample G	**2**	**4**	**1**	**0**	**0**	**1**	**55**	**8**	**2**
Sample H	**0**	**4**	**0**	**1**	**0**	**0**	**64**	**6**	**8**
Sample I	**1**	**5**	**0**	**1**	**0**	**0**	**45**	**8**	**10**
Sample J	**1**	**4**	**0**	**1**	**0**	**0**	**48**	**7**	**7**
Sample K	**2**	**6**	**0**	**1**	**0**	**1**	**58**	**5**	**8**
Sample L	**2**	**4**	**0**	**1**	**0**	**1**	**51**	**9**	**4**
Sample M	**2**	**1**	**1**	**0**	**0**	**0**	**62**	**4**	**8**
Sample N	**8**	**6**	**0**	**1**	**0**	**0**	**52**	**7**	**5**
Sample O	**0**	**7**	**0**	**1**	**0**	**2**	**51**	**7**	**4**
Sample P	**2**	**4**	**0**	**1**	**0**	**0**	**48**	**14**	**3**

EXCLI Journal 2021;20:995-1008 – ISSN 1611-2156
Received: April 08, 2021, accepted: May 26, 2021, published: June 01, 2021

Sample Q	**2**	**2**	**1**	**0**	**0**	**2**	**49**	**5**	**3**
Sample R	**2**	**3**	**1**	**1**	**0**	**1**	**54**	**5**	**7**
Sample S	**1**	**2**	**0**	**1**	**0**	**2**	**67**	**2**	**6**
Sample T	**24**	**4**	**1**	**0**	**0**	**0**	**45**	**7**	**3**
Sample U	**2**	**4**	**0**	**1**	**0**	**0**	**61**	**7**	**3**
Sample V	**2**	**4**	**0**	**1**	**0**	**0**	**61**	**7**	**4**
Sample W	**1**	**5**	**0**	**1**	**0**	**1**	**55**	**5**	**5**
Sample X	**3**	**4**	**0**	**1**	**0**	**1**	**53**	**6**	**4**
Sample Y	**0**	**2**	**0**	**1**	**0**	**1**	**50**	**9**	**5**
Sample Z	**1**	**5**	**0**	**0**	**0**	**2**	**61**	**8**	**4**
Sample AA	**0**	**4**	**0**	**1**	**0**	**2**	**47**	**5**	**2**
Sample AB	**1**	**4**	**0**	**1**	**0**	**2**	**44**	**6**	**6**
Sample AC	**3**	**4**	**0**	**1**	**0**	**2**	**47**	**7**	**7**
Sample AD	**2**	**6**	**0**	**1**	**0**	**1**	**42**	**8**	**7**
Sample AE	**2**	**4**	**0**	**1**	**0**	**2**	**57**	**5**	**5**
Sample AF	**2**	**2**	**0**	**1**	**0**	**2**	**65**	**4**	**7**
Sample AG	**8**	**4**	**0**	**1**	**0**	**1**	**54**	**6**	**4**
Sample AH	**1**	**3**	**0**	**1**	**0**	**2**	**19**	**4**	**0**
Sample AI	**0**	**4**	**1**	**1**	**0**	**1**	**50**	**5**	**3**
Sample AJ	**1**	**4**	**0**	**0**	**0**	**0**	**31**	**3**	**1**
Sample AK	**1**	**3**	**0**	**1**	**0**	**0**	**53**	**8**	**2**
Sample AL	**1**	**2**	**0**	**0**	**0**	**0**	**57**	**12**	**7**
Sample AM	**2**	**2**	**0**	**0**	**0**	**0**	**58**	**12**	**9**

EXCLI Journal 2021;20:995-1008 – ISSN 1611-2156
Received: April 08, 2021, accepted: May 26, 2021, published: June 01, 2021

Sample AN	**2**	**2**	**0**	**1**	**0**	**0**	**52**	**6**	**4**
Sample AO	**4**	**3**	**0**	**1**	**0**	**0**	**51**	**12**	**7**
Sample AP	**1**	**3**	**0**	**0**	**0**	**2**	**52**	**8**	**3**
Sample AQ	**3**	**4**	**0**	**1**	**0**	**2**	**47**	**7**	**7**
Sample AR	**0**	**4**	**0**	**1**	**0**	**1**	**38**	**4**	**1**
Sample AS	**2**	**4**	**0**	**1**	**0**	**0**	**45**	**6**	**5**
Sample AT	**1**	**3**	**0**	**1**	**0**	**0**	**46**	**8**	**5**
Sample AU	**0**	**3**	**0**	**1**	**0**	**0**	**54**	**8**	**4**

EXCLI Journal 2021;20:995-1008 – ISSN 1611-2156
Received: April 08, 2021, accepted: May 26, 2021, published: June 01, 2021

Sample A
Number of peaks: 116
Number of identified compounds: 72
Ratio of identified peak area: 86.88%

Peak Area [%]	Database comparison
**0.06**	7-Tetradecene
**0.06**	Cyclotetrasiloxane, octamethyl-
**0.06**	Decane, 1-iodo-
**0.06**	Oxygen
**0.08**	Undecane, 3-methyl-
**0.1**	Nonane, 5-methyl-5-propyl-
**0.11**	Heptane, 2,5,5-trimethyl-
**0.12**	Isotridecanol-
**0.13**	Octacosyl trifluoroacetate
**0.14**	1,3-Cyclopentadiene, 1,3-bis(1-methylethyl)-
**0.14**	Dodecane, 4,6-dimethyl-
**0.16**	Dodecane, 4,6-dimethyl-
**0.17**	3-Eicosene, (E)-
**0.18**	Heptane, 2,5,5-trimethyl-
**0.19**	Decane, 1,1'-oxybis-
**0.19**	Dodecane, 2,6,10-trimethyl-
**0.19**	Dodecane, 4,6-dimethyl-
**0.2**	Zinc, bis[2-(1,1-dimethyl-2-propenyl)-3,3-dimethylcyclopropyl]-, [1.alpha.(1R*,2R*),2.alpha.]-
**0.22**	p-Xylene
**0.25**	Hexadecane, 1-iodo-
**0.26**	Eicosane
**0.27**	Eicosane
**0.29**	3-Hexen-1-ol, 6-(2,6,6-trimethyl-1-cyclohexenyl)-4-methyl-, (E)-
**0.3**	Decane
**0.31**	6,10-Dodecadien-3-ol, 3,7,11-trimethyl-
**0.31**	Dodecane, 4,6-dimethyl-
**0.34**	Dodecane, 4,6-dimethyl-
**0.36**	Disulfide, di-tert-dodecyl
**0.36**	Octane, 2,6,6-trimethyl-
**0.37**	.alpha.-Farnesene
**0.37**	Eicosane
**0.47**	Dodecane, 4,6-dimethyl-
**0.53**	Dodecane, 4,6-dimethyl-
**0.58**	Octane, 5-ethyl-2-methyl-
**0.6**	1H-Cycloprop[e]azulene, 1a,2,3,4,4a,5,6,7b-octahydro-1,1,4,7-tetramethyl-, [1aR-(1a.alpha.,4.alpha.,4a.beta.,7b.alpha.)]-
**0.61**	1,3-Cyclopentadiene, 1,3-bis(1-methylethyl)-

EXCLI Journal 2021;20:995-1008 – ISSN 1611-2156
Received: April 08, 2021, accepted: May 26, 2021, published: June 01, 2021

**0.62**	Squalene
**0.67**	2,6,10-Dodecatrien-1-ol, 3,7,11-trimethyl-
**0.69**	1-Octanol, 2,7-dimethyl-
**0.76**	Tetradecane
**0.78**	Isolongifolene, 9,10-dehydro-
**0.79**	2-Undecene, 4,5-dimethyl-, [R*,R*-(E)]-
**0.79**	Dodecane, 4-methyl-
**0.8**	Eicosane
**0.81**	Dodecane, 4,6-dimethyl-
**0.85**	Tricyclo[3.1.0.0(2,4)]hexane, 3,6-diethyl-3,6-dimethyl-, trans-
**0.93**	1-Hexadecanesulfonyl chloride
**0.94**	Nonane, 3-methyl-5-propyl-
**0.99**	Isotridecanol-
**1.06**	Dodecane, 4,6-dimethyl-
**1.16**	Dodecane, 4,6-dimethyl-
**1.23**	Undecane
**1.25**	Dodecane, 4,6-dimethyl-
**1.37**	Phosphinous chloride, phenyl(1,7,7-trimethylbicyclo[2.2.1]hept-2-yl)-
**1.39**	Dodecane, 4,6-dimethyl-
**1.45**	Undecane, 5-methyl-
**1.46**	Octane, 5-ethyl-2-methyl-
**1.53**	1H-Cyclopropa[a]naphthalene, 1a,2,3,3a,4,5,6,7b-octahydro-1,1,3a,7-tetramethyl-, [1aR-(1a.alpha.,3a.alpha.,7b.alpha.)]-
**1.63**	Eicosane
**1.68**	1H-3a,7-Methanoazulene, 2,3,6,7,8,8a-hexahydro-1,4,9,9-tetramethyl-, (1.alpha.,3a.alpha.,7.alpha.,8a.beta.)-
**1.79**	(2,6,6-Trimethylcyclohex-1-enylmethanesulfonyl)benzene
**1.97**	Eicosane
**1.98**	5,9-Undecadien-1-yne, 6,10-dimethyl-
**2.19**	1,5,9-Cyclododecatriene, 1,5,9-trimethyl-
**2.25**	Dodecane
**2.92**	Eicosane
**3.55**	Dodecane, 4,6-dimethyl-
**4.06**	1-Heptanol, 2,4-diethyl-
**4.54**	1-Heptanol, 2,4-diethyl-
**4.59**	Isotridecanol-
**8.88**	Dodecane, 4,6-dimethyl-
**13.39**	Butylated Hydroxytoluene

EXCLI Journal 2021;20:995-1008 – ISSN 1611-2156
Received: April 08, 2021, accepted: May 26, 2021, published: June 01, 2021

Sample B
Number of peaks: 102
Number of identified compounds: 85
Ratio of identified peak area: 98.86%

Peak Area [%]	Database comparison
**0.04**	Decane, 5,6-dimethyl-
**0.06**	Cyclopentasiloxane, decamethyl-
**0.06**	Eicosane
**0.06**	Heptadecane
**0.06**	Nonane, 5-(2-methylpropyl)-
**0.06**	Nonane, 5-(2-methylpropyl)-
**0.07**	2-Bromo dodecane
**0.07**	Dodecane, 4,6-dimethyl-
**0.07**	Nonane, 5-butyl-
**0.07**	Tridecane
**0.08**	2,6-Dimethyldecane
**0.08**	Decane
**0.08**	Decane, 1-iodo-
**0.08**	o-Xylene
**0.08**	Tetradecane
**0.08**	Undecyl trifluoroacetate
**0.09**	Eicosane
**0.1**	Decanal
**0.1**	Hexadecane
**0.12**	Decane, 3,3,8-trimethyl-
**0.12**	Eicosane
**0.13**	Oxalic acid, 2-ethylhexyl hexyl ester
**0.14**	1-Undecene, 4-methyl-
**0.14**	Cyclooctane, ethyl-
**0.15**	Eicosane
**0.15**	Tetradecane
**0.16**	Heneicosane
**0.16**	Hexasiloxane, tetradecamethyl-
**0.17**	2-Undecene, 4,5-dimethyl-, [R*,R*-(E)]-
**0.17**	3-Decene, 2,2-dimethyl-, (E)-
**0.18**	Dodecane, 2,7,10-trimethyl-
**0.18**	Dodecane, 4,6-dimethyl-
**0.19**	Undecane
**0.2**	Tetradecane
**0.22**	Cyclooctane, (1-methylpropyl)-
**0.23**	Octadecane
**0.24**	Nonane, 2,5-dimethyl-
**0.25**	Heptasiloxane, hexadecamethyl-
**0.27**	Cyclooctane, butyl-
**0.28**	1-Decanol, 2-hexyl-

EXCLI Journal 2021;20:995-1008 – ISSN 1611-2156
Received: April 08, 2021, accepted: May 26, 2021, published: June 01, 2021

**0.31**	Dodecane, 4,6-dimethyl-
**0.36**	Tetradecane
**0.37**	Dodecane, 4,6-dimethyl-
**0.37**	Dodecane, 4,6-dimethyl-
**0.37**	Heneicosane
**0.37**	Isotridecanol-
**0.39**	Dodecane, 4,6-dimethyl-
**0.4**	1-Undecene, 7-methyl-
**0.41**	Dodecane
**0.43**	Tetradecane
**0.52**	Octane, 2,4,6-trimethyl-
**0.57**	Octane, 2,3,6,7-tetramethyl-
**0.58**	Caprolactam
**0.63**	Heptasiloxane, hexadecamethyl-
**0.66**	Dodecane, 4,6-dimethyl-
**0.66**	Eicosane
**0.7**	Undecane
**0.88**	Dodecane, 4,6-dimethyl-
**0.88**	Eicosane
**0.91**	Dodecane, 4-methyl-
**0.92**	Nonane, 2,5-dimethyl-
**0.99**	Dodecane, 4,6-dimethyl-
**1.1**	Dodecane, 4,6-dimethyl-
**1.15**	Hexadecane
**1.19**	Eicosane
**1.25**	Dodecane, 4,6-dimethyl-
**1.42**	Dodecane, 4,6-dimethyl-
**1.43**	4-Decene, 7-methyl-, (E)-
**1.61**	Dodecane, 4,6-dimethyl-
**1.73**	Dodecane, 4,6-dimethyl-
**2.17**	Dodecane, 4,6-dimethyl-
**2.76**	Octane, 5-ethyl-2-methyl-
**2.79**	Isotridecanol-
**3.01**	Isotridecanol-
**3.93**	Octane, 2,3,6,7-tetramethyl-
**4.03**	Isotridecanol-
**4.36**	Dodecane, 4,6-dimethyl-
**4.47**	Octane, 6-ethyl-2-methyl-
**4.6**	1-Undecene, 7-methyl-
**5.2**	Dodecane, 4,6-dimethyl-
**5.24**	1-Undecene, 7-methyl-
**5.38**	Heptane, 2,5,5-trimethyl-
**5.64**	Heptane, 2,5,5-trimethyl-
**7.43**	Octane, 5-ethyl-2-methyl-
**9.05**	Octane, 6-ethyl-2-methyl-

EXCLI Journal 2021;20:995-1008 – ISSN 1611-2156
Received: April 08, 2021, accepted: May 26, 2021, published: June 01, 2021

Sample C
Number of peaks: 81
Number of identified compounds: 68
Ratio of identified peak area: 97.51%

Peak Area [%]	Database comparison
0.04	Heptane, 3,3,4-trimethyl-
0.08	2-Isopropyl-5-methyl-1-heptanol
0.08	Heneicosane
0.09	5,5-Dibutylnonane
0.1	Hexadecane, 2,6,11,15-tetramethyl-
0.11	Tetradecane
0.12	Cyclopentasiloxane, decamethyl-
0.12	Heneicosane
0.16	Dodecane, 4-methyl-
0.16	Nonane, 3-methyl-
0.16	Triacontane, 1-bromo-
0.18	Tetradecane
0.2	Eicosane
0.2	Nonane, 5-methyl-5-propyl-
0.2	Tetradecanal
0.21	1-Tridecene
0.23	Decane, 2,8,8-trimethyl-
0.24	p-Xylene
0.3	Dodecane, 4,6-dimethyl-
0.31	Hexasiloxane, tetradecamethyl-
0.33	1-Heptanol, 2,4-diethyl-
0.33	Sulfurous acid, butyl dodecyl ester
0.37	Eicosane
0.4	Eicosane
0.41	1-Undecene, 7-methyl-
0.41	Undecane
0.42	1-Tridecene
0.45	Cyclopentane, 1-pentyl-2-propyl-
0.45	Eicosane
0.47	1-Undecene, 7-methyl-
0.47	Heneicosane
0.49	Dodecane, 4,6-dimethyl-
0.5	Dodecane, 4,6-dimethyl-
0.53	Dodecane, 4,6-dimethyl-
0.54	1-Butanol, 3-methoxy-
0.59	Dodecane, 4,6-dimethyl-
0.59	Heneicosane
0.7	Undecane, 4-methyl-
0.75	Octane, 5-ethyl-2-methyl-
0.86	Dodecane, 4,6-dimethyl-

EXCLI Journal 2021;20:995-1008 – ISSN 1611-2156
Received: April 08, 2021, accepted: May 26, 2021, published: June 01, 2021

**0.89**	Hexadecane, 2,6,11,15-tetramethyl-
**0.9**	Decane, 3,3,6-trimethyl-
**1**	Eicosane
**1.05**	Dodecane, 4-methyl-
**1.05**	Heptasiloxane, hexadecamethyl-
**1.21**	Cyclopentanone, 2-methyl-
**1.21**	Tetradecane
**1.29**	Dodecane, 4,6-dimethyl-
**1.46**	Eicosane
**1.46**	Octane, 5-ethyl-2-methyl-
**1.58**	Eicosane
**1.81**	Dodecane, 4,6-dimethyl-
**1.82**	Octane, 5-ethyl-2-methyl-
**1.93**	Dodecane
**2.17**	Caprolactam
**2.32**	Dodecane, 4,6-dimethyl-
**2.37**	Undecane, 5-methyl-
**2.45**	Isotridecanol-
**2.82**	Eicosane
**2.97**	Dodecane, 4,6-dimethyl-
**3.11**	Isotridecanol-
**3.74**	Isotridecanol-
**3.76**	Dodecane, 4,6-dimethyl-
**3.89**	Dodecane, 4,6-dimethyl-
**4.3**	Dodecane, 4,6-dimethyl-
**5.42**	Eicosane
**9.18**	Dodecane, 4,6-dimethyl-
**17**	Dodecane, 4,6-dimethyl-

EXCLI Journal 2021;20:995-1008 – ISSN 1611-2156
Received: April 08, 2021, accepted: May 26, 2021, published: June 01, 2021

Sample D
Number of peaks: 120
Number of identified compounds: 83
Ratio of identified peak area: 95.33%

Peak Area [%]	Database comparison
**0.05**	2,3-Dimethyldecane
**0.05**	Decane, 2,8,8-trimethyl-
**0.06**	Heptadecane, 2-methyl-
**0.07**	1-Butanol, 3-methoxy-, acetate
**0.09**	1-Octanol, 2-butyl-
**0.09**	Octane, 2,6-dimethyl-
**0.1**	Hexadecane, 2-methyl-
**0.1**	Octane, 2,6,6-trimethyl-
**0.11**	Decane, 3-ethyl-3-methyl-
**0.11**	Hexadecane
**0.12**	1-Dodecene
**0.12**	1-Pentadecene
**0.12**	Dodecane, 2,6,10-trimethyl-
**0.13**	Cyclotetrasiloxane, octamethyl-
**0.13**	Tetradecane, 4-methyl-
**0.15**	Dodecane, 4,6-dimethyl-
**0.15**	Hexadecane, 1-iodo-
**0.16**	4-Decene, 7-methyl-, (E)-
**0.17**	Octadecane, 1-(ethenyloxy)-
**0.18**	2-Isopropyl-5-methyl-1-heptanol
**0.18**	Cyclopentasiloxane, decamethyl-
**0.18**	Decane, 5-propyl-
**0.19**	Decane, 2,8,8-trimethyl-
**0.19**	Tetracontane
**0.2**	Pentadecane, 2,6,10,14-tetramethyl-
**0.21**	Hexadecane
**0.22**	Acetamide, N,N-dimethyl-
**0.24**	Heneicosane
**0.24**	Heptane, 2,5,5-trimethyl-
**0.24**	Hexadecane
**0.24**	Octadecane
**0.25**	Eicosane
**0.26**	Cyclopentane, undecyl-
**0.26**	Dodecane, 4,6-dimethyl-
**0.27**	Isotridecanol-
**0.27**	Pentadecane, 2,6,10,14-tetramethyl-
**0.28**	Heptadecane
**0.29**	6,10,13-Trimethyltetradecanol
**0.32**	Eicosane
**0.35**	Eicosane

EXCLI Journal 2021;20:995-1008 – ISSN 1611-2156
Received: April 08, 2021, accepted: May 26, 2021, published: June 01, 2021

**0.36**	Hexasiloxane, tetradecamethyl-
**0.37**	Hexadecane, 2,6,11,15-tetramethyl-
**0.42**	Cyclopropane, 1,1-dimethyl-2-(3-methyl-1,3-butadienyl)-
**0.42**	Hexadecane
**0.43**	Hexadecane
**0.46**	Dodecane, 4,6-dimethyl-
**0.48**	Dodecane, 4-methyl-
**0.59**	1-Tetradecene
**0.62**	Eicosane
**0.66**	Eicosane
**0.68**	Heptasiloxane, hexadecamethyl-
**0.73**	Dodecane, 4,6-dimethyl-
**0.73**	Hexadecane, 2,6,11,15-tetramethyl-
**0.73**	Undecane
**0.9**	Eicosane
**0.91**	Hexadecane
**1**	Dodecane, 4,6-dimethyl-
**1.06**	D-Limonene
**1.18**	Dodecane, 4-methyl-
**1.21**	Sulfurous acid, pentyl tridecyl ester
**1.22**	Heptane, 2,5,5-trimethyl-
**1.34**	Eicosane
**1.45**	Nonane, 2,6-dimethyl-
**1.56**	Dodecane, 4,6-dimethyl-
**1.73**	1-Heptanol, 2,4-diethyl-
**1.73**	Isotridecanol-
**1.84**	1-Butanol, 3-methoxy-
**1.94**	Dodecane, 4,6-dimethyl-
**1.97**	2-Undecene, 2,5-dimethyl-
**1.99**	Octane, 2,3,6,7-tetramethyl-
**2.14**	Dodecane, 4,6-dimethyl-
**2.15**	Caprolactam
**2.27**	Dodecane
**2.31**	1-Heptanol, 2,4-diethyl-
**2.36**	1-Undecene, 7-methyl-
**2.49**	Dodecane, 4,6-dimethyl-
**2.68**	Eicosane
**3.59**	Undecane, 5-methyl-
**3.62**	Undecane, 5-methyl-
**5.11**	Dodecane, 4,6-dimethyl-
**6.85**	Octane, 5-ethyl-2-methyl-
**10.31**	Octane, 5-ethyl-2-methyl-
**11.6**	Dodecane, 4,6-dimethyl-

EXCLI Journal 2021;20:995-1008 – ISSN 1611-2156
Received: April 08, 2021, accepted: May 26, 2021, published: June 01, 2021

Sample E
Number of peaks: 120
Number of identified compounds: 85
Ratio of identified peak area: 97.69%

Peak Area [%]	Database comparison
**0.03**	Nonane, 5-methyl-5-propyl-
**0.03**	Eicosane
**0.04**	Decane, 3,3,8-trimethyl-
**0.05**	Tetracosane
**0.06**	2-Undecene, 3-methyl-, (Z)-
**0.06**	1-Undecene, 8-methyl-
**0.06**	1-Nonanol, 4,8-dimethyl-
**0.07**	Undecane, 2-methyl-
**0.07**	Ethanone, 1-[4-(1-methylethenyl)phenyl]-
**0.07**	3-Octadecene, (E)-
**0.07**	1-Hexadecanesulfonyl chloride
**0.08**	Cyclopentane, decyl-
**0.08**	Cyclohexane, 1,2-diethyl-3-methyl-
**0.09**	Dodecane, 2-methyl-
**0.09**	Decane, 1-iodo-
**0.1**	Dodecane, 2,6,11-trimethyl-
**0.1**	2-Isopropyl-5-methyl-1-heptanol
**0.11**	Dodecane, 4,6-dimethyl-
**0.11**	2-Undecene, 9-methyl-, (E)-
**0.12**	Disulfide, di-tert-dodecyl
**0.13**	Heneicosane
**0.13**	Dodecane, 2,6,10-trimethyl-
**0.14**	Isotridecanol-
**0.14**	Dodecane, 4,6-dimethyl-
**0.14**	Decane, 2,8,8-trimethyl-
**0.15**	Dodecane, 4,6-dimethyl-
**0.15**	Bicyclo[3.1.1]heptane, 6,6-dimethyl-2-methylene-, (1S)-
**0.16**	3-Octanol, 6-ethyl-
**0.17**	Oxalic acid, 6-ethyloct-3-yl heptyl ester
**0.17**	Nonane, 5-methyl-5-propyl-
**0.17**	2-Bromo dodecane
**0.17**	1-Tetradecene
**0.17**	1-Pentadecene
**0.18**	Nonane, 5-methyl-5-propyl-
**0.18**	5,8-Diethyl-6-dodecanol
**0.2**	Tridecane, 7-methylene-
**0.21**	Nonane, 5-methyl-5-propyl-
**0.22**	2-Undecene, 9-methyl-, (Z)-
**0.24**	Sulfurous acid, dodecyl pentyl ester
**0.24**	Eicosane

EXCLI Journal 2021;20:995-1008 – ISSN 1611-2156
Received: April 08, 2021, accepted: May 26, 2021, published: June 01, 2021

**0.24**	Dodecane, 4-methyl-
**0.25**	Decane, 1,1'-oxybis-
**0.28**	D-Limonene
**0.31**	Octane, 2,3,6,7-tetramethyl-
**0.32**	Tetradecane
**0.32**	Eicosane
**0.32**	Cyclododecane
**0.32**	1R-.alpha.-Pinene
**0.35**	Dodecane, 4,6-dimethyl-
**0.36**	Cyclopentane, pentyl-
**0.36**	4-Decene, 7-methyl-, (E)-
**0.37**	Dodecane, 4,6-dimethyl-
**0.4**	Eicosane
**0.42**	Nonane, 5-methyl-5-propyl-
**0.46**	Decane, 2,8,8-trimethyl-
**0.52**	1-Tridecene
**0.53**	Cyclohexane, undecyl-
**0.54**	Eicosane
**0.55**	Dodecane, 4,6-dimethyl-
**0.57**	Undecane, 3-methyl-
**0.67**	Dodecane, 4,6-dimethyl-
**0.73**	Dodecane, 4,6-dimethyl-
**0.81**	Sulfurous acid, octadecyl pentyl ester
**0.82**	3-Carene
**0.9**	Sulfurous acid, pentyl tridecyl ester
**0.91**	Dodecane, 4,6-dimethyl-
**1.04**	2-Undecene, 2,5-dimethyl-
**1.26**	Heptane, 2,5,5-trimethyl-
**1.43**	1-Heptanol, 2,4-diethyl-
**1.44**	Octane, 5-ethyl-2-methyl-
**1.56**	Isotridecanol-
**1.58**	Octane, 2,6,6-trimethyl-
**1.6**	Dodecane, 4,6-dimethyl-
**2.01**	1-Heptanol, 2,4-diethyl-
**2.19**	2-Undecene, 2,5-dimethyl-
**2.43**	o-Xylene
**2.52**	Dodecane, 4,6-dimethyl-
**2.66**	Octane, 5-ethyl-2-methyl-
**2.81**	1-Undecene, 7-methyl-
**3.6**	Decane
**3.92**	Octane, 5-ethyl-2-methyl-
**5.56**	Dodecane, 4-methyl-
**10.03**	Tridecane
**14.41**	Undecane
**18.06**	Dodecane

EXCLI Journal 2021;20:995-1008 – ISSN 1611-2156
Received: April 08, 2021, accepted: May 26, 2021, published: June 01, 2021

Sample F
Number of peaks: 104
Number of identified compounds: 80
Ratio of identified peak area: 97.81%

Peak Area [%]	Database comparison
0.06	Heneicosane
0.06	Heneicosane
0.08	Cyclopentane, decyl-
0.08	Heneicosane
0.09	Nonacosane
0.09	Tetradecane
0.09	Undecane, 3-methyl-
0.1	Heptadecane
0.11	Eicosane
0.11	Undecane
0.12	Tetracosane
0.13	Decane, 3,3,8-trimethyl-
0.14	1-Undecene, 4-methyl-
0.14	2-Isopropyl-5-methyl-1-heptanol
0.14	Dodecane, 2,6,10-trimethyl-
0.15	Tetradecane
0.16	2-Undecene, 2,5-dimethyl-
0.17	2,5-Cyclohexadiene-1,4-dione, 2,6-bis(1,1-dimethylethyl)-
0.17	4-Decene, 7-methyl-, (E)-
0.17	Dodecane, 4,6-dimethyl-
0.17	Eicosane
0.17	Hexadecane, 1-iodo-
0.18	1-Heptanol, 2,4-diethyl-
0.18	2-Propenoic acid, 2-methyl-, octyl ester
0.18	Eicosane
0.19	Pentadecane
0.2	2-Bromo dodecane
0.21	Octane, 2,3,6,7-tetramethyl-
0.22	Dodecane, 4,6-dimethyl-
0.23	Heptasiloxane, hexadecamethyl-
0.25	Decanal
0.28	Tetradecane, 4-methyl-
0.29	Cyclooctasiloxane, hexadecamethyl-
0.34	p-Xylene
0.36	Dodecane, 4,6-dimethyl-
0.37	Tetradecane
0.43	Octane, 1,1'-oxybis-
0.45	Octacosyl trifluoroacetate
0.47	1-Methoxy-2-propyl acetate
0.47	Dodecane, 4,6-dimethyl-

EXCLI Journal 2021;20:995-1008 – ISSN 1611-2156
Received: April 08, 2021, accepted: May 26, 2021, published: June 01, 2021

**0.48**	Dodecane, 4,6-dimethyl-
**0.48**	Undecane
**0.49**	2,2,4,4-Tetramethyloctane
**0.5**	Dodecane, 4,6-dimethyl-
**0.55**	Isotridecanol-
**0.61**	Octane, 5-ethyl-2-methyl-
**0.63**	Octane, 3,3-dimethyl-
**0.66**	Dodecane, 4,6-dimethyl-
**0.67**	Dodecane, 4,6-dimethyl-
**0.78**	Dodecane, 4,6-dimethyl-
**0.85**	Cyclopentanone, 2-methyl-
**0.88**	1-Butanol, 3-methoxy-
**0.89**	Dodecane, 4,6-dimethyl-
**0.94**	Cycloheptasiloxane, tetradecamethyl-
**0.99**	Octacosane
**1**	Dodecane, 4-methyl-
**1.06**	Dodecane, 4,6-dimethyl-
**1.14**	Dodecane, 4,6-dimethyl-
**1.21**	Eicosane
**1.22**	Dodecane, 4,6-dimethyl-
**1.24**	Eicosane
**1.27**	Octane, 5-ethyl-2-methyl-
**1.33**	Dodecane
**1.37**	Eicosane
**1.48**	Octane, 2,3,6,7-tetramethyl-
**1.67**	Heptasiloxane, hexadecamethyl-
**1.7**	1-Undecene, 7-methyl-
**1.73**	Dodecane, 4-methyl-
**1.82**	Octane, 5-ethyl-2-methyl-
**1.87**	Dodecane, 4,6-dimethyl-
**2**	1-Undecene, 7-methyl-
**2.41**	Octane, 5-ethyl-2-methyl-
**3.68**	Isotridecanol-
**3.7**	Decane, 2,2-dimethyl-
**3.75**	Dodecane, 4,6-dimethyl-
**3.81**	1-Heptanol, 2,4-diethyl-
**4.53**	Dodecane, 4,6-dimethyl-
**5.91**	1-Heptanol, 2,4-diethyl-
**11.68**	Heptasiloxane, hexadecamethyl-
**16.83**	Hexasiloxane, tetradecamethyl-

EXCLI Journal 2021;20:995-1008 – ISSN 1611-2156
Received: April 08, 2021, accepted: May 26, 2021, published: June 01, 2021

Sample G
Number of peaks: 103
Number of identified compounds: 76
Ratio of identified peak area: 96.90%

Peak Area [%]	Database comparison
**0.07**	Dodecane, 2-methyl-
**0.07**	Hexadecane, 2,6,11,15-tetramethyl-
**0.12**	Tetradecane, 4-methyl-
**0.13**	Hexadecane, 2-methyl-
**0.13**	Pentatriacontane
**0.14**	Hexadecane, 2,6,11,15-tetramethyl-
**0.14**	Nonane, 3-methyl-5-propyl-
**0.15**	Tetradecane, 4-methyl-
**0.16**	Heneicosane
**0.17**	Hexatriacontane
**0.17**	Pentadecane, 2,6,10-trimethyl-
**0.18**	Benzenecarboxylic acid
**0.18**	Nonane, 5-butyl-
**0.18**	Sulfurous acid, pentyl undecyl ester
**0.2**	1-Hexanol, 5-methyl-2-(1-methylethyl)-
**0.22**	5,5-Dibutylnonane
**0.23**	Heneicosane
**0.24**	Decane
**0.24**	Eicosane
**0.25**	Cyclopentasiloxane, decamethyl-
**0.26**	1-Decanol, 2-hexyl-
**0.27**	Cyclotetrasiloxane, octamethyl-
**0.28**	Decanal
**0.28**	Dodecane, 4,6-dimethyl-
**0.28**	Nonane, 3-methyl-5-propyl-
**0.29**	1-Butanol, 3-methoxy-
**0.32**	D-Limonene
**0.32**	Hexadecane
**0.33**	Undecyl heptafluorobutyrate
**0.34**	Heptadecane, 8-methyl-
**0.34**	Octane, 2,6,6-trimethyl-
**0.35**	2-Dodecene, (Z)-
**0.35**	Undecane
**0.37**	4-Decene, 7-methyl-, (E)-
**0.37**	Dodecane, 4,6-dimethyl-
**0.4**	Isotridecanol-
**0.4**	Octane, 2,6,6-trimethyl-
**0.41**	Octacosane
**0.45**	Hexadecane, 2,6,11,15-tetramethyl-
**0.46**	Decane, 5-propyl-

EXCLI Journal 2021;20:995-1008 – ISSN 1611-2156
Received: April 08, 2021, accepted: May 26, 2021, published: June 01, 2021

**0.57**	Dodecane, 4,6-dimethyl-
**0.63**	Dodecane, 4,6-dimethyl-
**0.64**	Dodecane, 4,6-dimethyl-
**0.66**	Isotridecanol-
**0.8**	Dodecane, 4,6-dimethyl-
**0.85**	Dodecane, 4,6-dimethyl-
**0.86**	Dodecane, 4,6-dimethyl-
**0.87**	Tetradecane
**1.05**	Dodecane, 4,6-dimethyl-
**1.14**	Dodecane, 2,6,11-trimethyl-
**1.16**	Heptasiloxane, hexadecamethyl-
**1.17**	Octacosane
**1.18**	Dodecane, 4,6-dimethyl-
**1.22**	Dodecane, 4,6-dimethyl-
**1.24**	Dodecane, 4,6-dimethyl-
**1.4**	Dodecane, 4,6-dimethyl-
**1.56**	Octacosane
**1.66**	Dodecane
**1.85**	Octane, 2,3,6,7-tetramethyl-
**1.92**	Dodecane, 4,6-dimethyl-
**1.98**	Eicosane
**2**	Eicosane
**2.09**	Heptane, 2,5,5-trimethyl-
**2.26**	Hexasiloxane, tetradecamethyl-
**2.26**	Octane, 5-ethyl-2-methyl-
**2.41**	Heptane, 2,5,5-trimethyl-
**3.49**	1-Undecene, 7-methyl-
**3.58**	2-Isopropyl-5-methyl-1-heptanol
**3.76**	Isotridecanol-
**3.8**	Octane, 5-ethyl-2-methyl-
**4**	1-Undecene, 7-methyl-
**4.98**	Dodecane, 4,6-dimethyl-
**5.2**	Isotridecanol-
**6.91**	Octane, 5-ethyl-2-methyl-
**7.15**	Hexadecane
**8.36**	Octane, 2,3,6,7-tetramethyl-

EXCLI Journal 2021;20:995-1008 – ISSN 1611-2156
Received: April 08, 2021, accepted: May 26, 2021, published: June 01, 2021

Sample H
Number of peaks: 142
Number of identified compounds: 92
Ratio of identified peak area: 94.01%

Peak Area [%]	Database comparison
**0.05**	Octane, 2,6,6-trimethyl-
**0.05**	Undecane, 2-methyl-
**0.07**	1-Butanol, 3-methoxy-, acetate
**0.07**	Tetradecane, 5-methyl-
**0.08**	Decane, 4-ethyl-
**0.08**	Dodecane, 3-methyl-
**0.09**	Dodecane
**0.09**	Tetradecane, 4-methyl-
**0.09**	Undecane, 5-methyl-
**0.11**	Undecane, 3-methyl-
**0.12**	Pentatriacontane
**0.13**	Dodecane, 4,6-dimethyl-
**0.13**	Dodecane, 4-methyl-
**0.14**	2-Isopropyl-5-methyl-1-heptanol
**0.14**	Isodecyl methacrylate
**0.14**	Pentadecane, 8-heptyl-
**0.15**	Dichloroacetic acid, 6-ethyl-3-octyl ester
**0.16**	3-Ethyl-3-methylheptane
**0.17**	Silane, cyclohexyldimethoxymethyl-
**0.18**	Dodecane, 4,6-dimethyl-
**0.18**	Ethanol, 2-(dodecyloxy)-
**0.21**	Cyclotetrasiloxane, octamethyl-
**0.21**	Propanoic acid, 2-methyl-, 1-(1,1-dimethylethyl)-2-methyl-1,3-propanediyl ester
**0.23**	1-Tridecene
**0.23**	Hexadecane
**0.23**	Hexadecane, 1-iodo-
**0.23**	Pentadecane, 8-hexyl-
**0.24**	10-Methylnonadecane
**0.24**	Tetradecane, 4-methyl-
**0.25**	Decane, 3,3,6-trimethyl-
**0.25**	Decane, 5-propyl-
**0.25**	Heneicosane
**0.26**	Heptadecane, 8-methyl-
**0.27**	4-Decene, 7-methyl-, (E)-
**0.3**	Decane, 2,8,8-trimethyl-
**0.3**	Hexadecane, 2,6,10,14-tetramethyl-
**0.32**	Eicosane
**0.32**	Hexadecane
**0.36**	n-Heptadecanol-1

EXCLI Journal 2021;20:995-1008 – ISSN 1611-2156
Received: April 08, 2021, accepted: May 26, 2021, published: June 01, 2021

**0.4**	Cyclopentanone, 2-methyl-
**0.4**	Cyclopentasiloxane, decamethyl-
**0.41**	1,3-Dioxolane-2-acetic acid, 2,4-dimethyl-, ethyl ester
**0.43**	Isotridecanol-
**0.44**	Hexadecane
**0.44**	Undecane
**0.46**	Tetrapentacontane, 1,54-dibromo-
**0.47**	Eicosane
**0.51**	Caprolactam
**0.53**	Hexadecane, 2,6,11,15-tetramethyl-
**0.55**	1-Methoxy-2-propyl acetate
**0.56**	Dodecane, 4,6-dimethyl-
**0.58**	Dodecane, 4,6-dimethyl-
**0.58**	Hexadecane, 2,6,10,14-tetramethyl-
**0.61**	Isotridecanol-
**0.63**	Heneicosane
**0.63**	Undecane, 3-methyl-
**0.68**	Dodecane, 4,6-dimethyl-
**0.73**	Hexadecane
**0.83**	Dodecane, 4,6-dimethyl-
**0.86**	Propanoic acid, 2-hydroxy-2-methyl-, methyl ester
**1.02**	Hexadecane
**1.06**	Heptane, 2,5,5-trimethyl-
**1.16**	Dodecane, 4,6-dimethyl-
**1.2**	Dodecane, 4,6-dimethyl-
**1.21**	Octane, 2,3,6,7-tetramethyl-
**1.24**	Pentadecane, 8-hexyl-
**1.27**	Tetradecane
**1.29**	Sulfurous acid, pentyl tridecyl ester
**1.39**	Dodecane, 4,6-dimethyl-
**1.42**	Nonane, 5-methyl-5-propyl-
**1.45**	Nonane, 2,6-dimethyl-
**1.61**	Dodecane, 4,6-dimethyl-
**1.62**	Octane, 5-ethyl-2-methyl-
**1.69**	Eicosane
**1.75**	Hexasiloxane, tetradecamethyl-
**1.77**	Eicosane
**1.88**	Decane
**1.9**	Dodecane, 4,6-dimethyl-
**1.91**	Dodecane, 4,6-dimethyl-
**1.92**	Undecane, 5-methyl-
**1.95**	Heptasiloxane, hexadecamethyl-
**2.25**	1-Decene, 2,4-dimethyl-
**2.35**	1-Undecene, 7-methyl-
**2.53**	Eicosane

EXCLI Journal 2021;20:995-1008 – ISSN 1611-2156
Received: April 08, 2021, accepted: May 26, 2021, published: June 01, 2021

**2.96**	Dodecane
**3.38**	Isotridecanol-
**3.61**	1-Heptanol, 2,4-diethyl-
**3.71**	Octane, 5-ethyl-2-methyl-
**4.33**	Dodecane, 4,6-dimethyl-
**4.48**	Undecane, 5-methyl-
**5.27**	1-Heptanol, 2,4-diethyl-
**8.58**	Dodecane, 4,6-dimethyl-

EXCLI Journal 2021;20:995-1008 – ISSN 1611-2156
Received: April 08, 2021, accepted: May 26, 2021, published: June 01, 2021

Sample I
Number of peaks: 95
Number of identified compounds: 72
Ratio of identified peak area: 96.46%

Peak Area [%]	Database comparison
0.14	Nonacosane
0.16	1-Butanol, 3-methoxy-, acetate
0.16	Undecane, 3,8-dimethyl-
0.17	Sulfurous acid, dodecyl hexyl ester
0.19	Dichloroacetic acid, 6-ethyl-3-octyl ester
0.2	Cyclopropane, nonyl-
0.21	Cyclotetrasiloxane, octamethyl-
0.22	Eicosane
0.26	Heptane, 2,5,5-trimethyl-
0.28	Eicosane
0.28	p-Xylene
0.3	Tetradecane, 4-methyl-
0.32	2-Undecene, 2,5-dimethyl-
0.39	Hexadecane, 2,6,10,14-tetramethyl-
0.39	Undecyl trifluoroacetate
0.4	Dodecane, 4-methyl-
0.44	Dodecane, 4,6-dimethyl-
0.44	Pentadecane, 7-methyl-
0.45	2-Isopropyl-5-methyl-1-heptanol
0.45	Dodecane, 2-methyl-
0.46	Caprolactam
0.47	Sulfurous acid, pentyl undecyl ester
0.48	Dodecane, 4,6-dimethyl-
0.49	Hexadecane, 1,1-bis(dodecyloxy)-
0.5	Decane, 5-propyl-
0.51	Isotridecanol-
0.54	2,2,4-Trimethyl-1,3-pentanediol diisobutyrate
0.57	Cyclopentane, decyl-
0.62	Acetic acid, trichloro-, heptyl ester
0.62	Heptasiloxane, hexadecamethyl-
0.64	Undecane, 2,6-dimethyl-
0.65	2-Bromo dodecane
0.73	Octane, 5-ethyl-2-methyl-
0.76	1-Methoxy-2-propyl acetate
0.76	Dodecane, 4,6-dimethyl-
0.78	1-Tridecene
0.85	Decane, 2,8,8-trimethyl-
0.87	Dodecane, 4,6-dimethyl-
0.87	Undecane, 4-methyl-

EXCLI Journal 2021;20:995-1008 – ISSN 1611-2156
Received: April 08, 2021, accepted: May 26, 2021, published: June 01, 2021

**0.89**	Propanoic acid, 2-methyl-, 3-hydroxy-2,4,4-trimethylpentyl ester
**0.95**	Cyclopentasiloxane, decamethyl-
**1.02**	Isotridecanol-
**1.04**	Nonane, 5-methyl-5-propyl-
**1.1**	Nonane, 5-(2-methylpropyl)-
**1.15**	Nonane, 5-methyl-5-propyl-
**1.24**	Dodecane, 4,6-dimethyl-
**1.24**	Tetradecane
**1.29**	Dodecane, 4-methyl-
**1.29**	Octane, 5-ethyl-2-methyl-
**1.31**	1-Undecene, 7-methyl-
**1.34**	Dodecane, 4,6-dimethyl-
**1.35**	Dodecane, 4,6-dimethyl-
**1.37**	1-Undecene, 7-methyl-
**1.56**	Sulfurous acid, pentyl tridecyl ester
**1.6**	Undecane
**1.62**	Octane, 5-ethyl-2-methyl-
**1.63**	Octacosane
**1.66**	Hexasiloxane, tetradecamethyl-
**1.87**	Dodecane, 4,6-dimethyl-
**1.89**	Heptasiloxane, hexadecamethyl-
**1.98**	Heneicosane
**2.05**	Octacosane
**2.23**	Hexadecane, 2,6,11,15-tetramethyl-
**2.29**	Octane, 5-ethyl-2-methyl-
**2.44**	Dodecane, 4,6-dimethyl-
**2.8**	1-Butanol, 3-methoxy-
**4.42**	Dodecane, 4,6-dimethyl-
**4.55**	Dodecane
**5.46**	2-Isopropyl-5-methyl-1-heptanol
**5.47**	Isotridecanol-
**8.01**	1-Heptanol, 2,4-diethyl-
**8.33**	Hexadecane

EXCLI Journal 2021;20:995-1008 – ISSN 1611-2156
Received: April 08, 2021, accepted: May 26, 2021, published: June 01, 2021

Sample J
Number of peaks: 110
Number of identified compounds: 74
Ratio of identified peak area: 95.75%

Peak Area [%]	Database comparison
0.06	Undecane, 3-methyl-
0.09	Nonane, 5-methyl-5-propyl-
0.1	Nonane, 5-methyl-5-propyl-
0.12	1-Dodecene
0.12	Pentatriacontane
0.12	Tetracosane
0.15	1-Butanol, 3-methoxy-, acetate
0.15	Decane, 3,3,8-trimethyl-
0.16	3-Eicosene, (E)-
0.17	p-Xylene
0.18	10-Methylnonadecane
0.19	Pentadecane, 8-hexyl-
0.23	Acetamide, N,N-dimethyl-
0.23	Propanoic acid, 2-methyl-, 1-(1,1-dimethylethyl)-2-methyl-1,3-propanediyl ester
0.24	Tetradecane, 4-methyl-
0.25	Dodecane, 4,6-dimethyl-
0.25	Tridecane
0.26	Cyclopentasiloxane, decamethyl-
0.26	Eicosane
0.26	Eicosane
0.3	1-Tetradecene
0.3	Octane, 2,6,6-trimethyl-
0.32	Dodecane, 4,6-dimethyl-
0.32	Ethanol, 2-butoxy-
0.32	Tetradecane, 3-methyl-
0.34	2-Bromo dodecane
0.39	4-Decene, 7-methyl-, (E)-
0.42	Dodecane, 4,6-dimethyl-
0.42	Trichloroacetic acid, hexadecyl ester
0.42	Tridecane, 4-methyl-
0.43	1-Decanol, 2-hexyl-
0.43	Isotridecanol-
0.44	Octadecane
0.46	Hexadecane, 2,6,10,14-tetramethyl-
0.49	Cyclotetrasiloxane, octamethyl-
0.52	Heptasiloxane, hexadecamethyl-
0.67	Undecyl trifluoroacetate
0.69	1-Methoxy-2-propyl acetate
0.7	Dodecane, 4,6-dimethyl-

EXCLI Journal 2021;20:995-1008 – ISSN 1611-2156
Received: April 08, 2021, accepted: May 26, 2021, published: June 01, 2021

**0.71**	Caprolactam
**0.73**	Dodecane, 4,6-dimethyl-
**0.74**	Octacosane
**0.78**	Undecane
**0.82**	2-Bromo dodecane
**0.83**	Cyclopentanone, 2-methyl-
**0.89**	Dodecane, 4,6-dimethyl-
**1.06**	Hexadecane
**1.08**	Tetradecane
**1.12**	Eicosane
**1.19**	Nonane, 5-methyl-5-propyl-
**1.27**	Eicosane
**1.31**	Heptane, 2,5,5-trimethyl-
**1.31**	Hexasiloxane, tetradecamethyl-
**1.37**	Dodecane, 4,6-dimethyl-
**1.39**	Heneicosane
**1.47**	Nonane, 2,6-dimethyl-
**1.5**	Dodecane, 4,6-dimethyl-
**1.53**	Sulfurous acid, 2-pentyl tridecyl ester
**1.54**	Dodecane, 4,6-dimethyl-
**1.54**	Tridecane, 5-methyl-
**2.18**	Octane, 2,3,6,7-tetramethyl-
**2.26**	1-Decene, 2,4-dimethyl-
**2.36**	Isotridecanol-
**2.43**	Dichloroacetic acid, 6-ethyl-3-octyl ester
**2.67**	Octane, 5-ethyl-2-methyl-
**2.88**	1-Undecene, 7-methyl-
**3.08**	Dodecane
**3.2**	1-Butanol, 3-methoxy-
**3.48**	1-Heptanol, 2,4-diethyl-
**4.53**	Nonane, 2,6-dimethyl-
**5**	Dodecane, 4,6-dimethyl-
**7.63**	Hexadecane
**8.2**	Octane, 5-ethyl-2-methyl-
**9.7**	Octane, 5-ethyl-2-methyl-

Sample K
Number of peaks: 128
Number of identified compounds: 95
Ratio of identified peak area: 96.44%

Peak Area [%]	Database comparison
**0.04**	Decane, 2,3,7-trimethyl-
**0.04**	Octacosyl trifluoroacetate
**0.04**	Tetradecane, 4-methyl-
**0.05**	Decane, 2,3,5-trimethyl-
**0.06**	Octane, 2,6-dimethyl-
**0.07**	3-(Hydroxy-phenyl-methyl)-2,3-dimethyl-octan-4-one
**0.07**	Pentadecane, 8-heptyl-
**0.08**	3-Tetradecene, (E)-
**0.08**	Pentadecane, 2,6,10,14-tetramethyl-
**0.08**	Pentadecane, 8-hexyl-
**0.1**	Nonadecyl trifluoroacetate
**0.11**	Eicosane
**0.11**	Undecane, 3,6-dimethyl-
**0.12**	Dodecane, 4,6-dimethyl-
**0.12**	Heptadecane
**0.12**	Heptasiloxane, hexadecamethyl-
**0.13**	1-Octanol, 2,7-dimethyl-
**0.13**	2-Undecene, 2,5-dimethyl-
**0.13**	Decane, 3,3,6-trimethyl-
**0.13**	Dichloroacetic acid, 6-ethyl-3-octyl ester
**0.13**	Hexadecane, 2,6,11,15-tetramethyl-
**0.15**	Heptadecane, 8-methyl-
**0.16**	Eicosane
**0.18**	Cyclotetrasiloxane, octamethyl-
**0.18**	Decanal
**0.18**	Heptadecane
**0.18**	Heptadecane, 8-methyl-
**0.19**	Tetradecane, 5-methyl-
**0.22**	Decane, 5-propyl-
**0.22**	Tetradecane, 4-methyl-
**0.23**	Octane, 2,6,6-trimethyl-
**0.25**	1-Heptanol, 2,4-diethyl-
**0.25**	Hexadecane
**0.25**	Nonadecane
**0.26**	Heptadecane, 8-methyl-
**0.26**	Tetradecane
**0.27**	1-Butanol, 3-methoxy-, acetate
**0.28**	Nonacosane
**0.28**	Octane, 2,3,6,7-tetramethyl-
**0.3**	Decane, 5-propyl-

EXCLI Journal 2021;20:995-1008 – ISSN 1611-2156
Received: April 08, 2021, accepted: May 26, 2021, published: June 01, 2021

**0.3**	Undecane, 3-methyl-
**0.34**	Dodecane, 4,6-dimethyl-
**0.34**	Eicosane
**0.34**	Pentadecane, 2,6,10,14-tetramethyl-
**0.35**	1-Octene, 3,7-dimethyl-
**0.35**	Hexadecane, 2,6,11,15-tetramethyl-
**0.36**	dl-2-Ethylhexyl chloroformate
**0.39**	2,2,4,4-Tetramethyloctane
**0.43**	p-Xylene
**0.44**	Tetradecane
**0.45**	Undecane
**0.46**	Cyclopentasiloxane, decamethyl-
**0.46**	Dodecane, 4-methyl-
**0.57**	Eicosane
**0.61**	Isotridecanol-
**0.63**	1-Hexadecanesulfonyl chloride
**0.63**	Dodecane, 4,6-dimethyl-
**0.67**	Dodecane, 4,6-dimethyl-
**0.71**	Dodecane, 4,6-dimethyl-
**0.81**	Dodecane, 4,6-dimethyl-
**0.91**	Dodecane, 4,6-dimethyl-
**0.96**	Octacosane
**0.97**	Sulfurous acid, pentyl tridecyl ester
**0.98**	Dodecane, 4,6-dimethyl-
**1.06**	Caprolactam
**1.11**	Dodecane, 4,6-dimethyl-
**1.14**	Heptasiloxane, hexadecamethyl-
**1.14**	Nonane, 5-methyl-5-propyl-
**1.16**	Heptane, 2,5,5-trimethyl-
**1.19**	Dodecane, 4,6-dimethyl-
**1.24**	1-Hexanol, 2-ethyl-
**1.32**	Octane, 2,3,6,7-tetramethyl-
**1.37**	1-Methoxy-2-propyl acetate
**1.39**	Hexasiloxane, tetradecamethyl-
**1.43**	Eicosane
**1.44**	Dodecane, 4,6-dimethyl-
**1.51**	Octane, 5-ethyl-2-methyl-
**1.69**	Dodecane, 4,6-dimethyl-
**1.78**	Eicosane
**2.03**	2-Propenoic acid, 2-ethylhexyl ester
**2.07**	Dodecane
**2.27**	2-Undecene, 2,5-dimethyl-
**2.46**	Heptasiloxane, hexadecamethyl-
**2.47**	Octane, 5-ethyl-2-methyl-
**2.5**	1-Undecene, 7-methyl-

EXCLI Journal 2021;20:995-1008 – ISSN 1611-2156
Received: April 08, 2021, accepted: May 26, 2021, published: June 01, 2021

**2.88**	Eicosane
**3.25**	Isotridecanol-
**3.33**	1-Heptanol, 2,4-diethyl-
**3.36**	Undecane, 2,5-dimethyl-
**3.92**	Dodecane, 4,6-dimethyl-
**4.37**	Octane, 5-ethyl-2-methyl-
**4.89**	1-Heptanol, 2,4-diethyl-
**4.91**	1-Butanol, 3-methoxy-
**5.02**	Octane, 5-ethyl-2-methyl-
**7.05**	Dodecane, 4,6-dimethyl-

EXCLI Journal 2021;20:995-1008 – ISSN 1611-2156
Received: April 08, 2021, accepted: May 26, 2021, published: June 01, 2021

Sample L
Number of peaks: 101
Number of identified compounds: 76
Ratio of identified peak area: 97.43%

Peak Area [%]	Database comparison
0.07	Tetradecanal
0.07	Undecane, 2-methyl-
0.09	Nonane, 5-(2-methylpropyl)-
0.12	Cyclopentane, nonyl-
0.13	Eicosane
0.15	Heneicosane
0.15	Undecane, 4,6-dimethyl-
0.16	1-Tetradecene
0.16	Cyclohexanol, 3,3,5-trimethyl-, acetate, cis-
0.16	Hexadecane
0.17	Eicosane
0.17	Heptadecane
0.17	Hexyl octyl ether
0.17	Nonane, 5-methyl-5-propyl-
0.18	Propanoic acid, 2-methyl-, 1-(1,1-dimethylethyl)-2-methyl-1,3-propanediyl ester
0.21	Cyclopentane, 1-pentyl-2-propyl-
0.21	Heptane, 2,5,5-trimethyl-
0.22	p-Xylene
0.24	2-Undecene, 4,5-dimethyl-, [R*,R*-(E)]-
0.24	Heptadecane
0.25	Dodecane, 4,6-dimethyl-
0.25	Eicosane
0.25	Tetracosane
0.26	Cyclopentasiloxane, decamethyl-
0.29	Eicosane
0.31	Decane, 2,8,8-trimethyl-
0.32	1-Undecene, 7-methyl-
0.33	Caprolactam
0.36	1-Dodecanol
0.38	Octane, 2,3,7-trimethyl-
0.4	1-Hexadecanesulfonyl chloride
0.4	Undecane, 3-methyl-
0.54	2-Bromo dodecane
0.55	Eicosane
0.61	Hexadecane, 2,6,11,15-tetramethyl-
0.63	Dodecane, 4,6-dimethyl-
0.63	Heptasiloxane, hexadecamethyl-
0.65	Phenol, 2,4-bis(1,1-dimethylethyl)-
0.66	Dodecane, 4,6-dimethyl-

EXCLI Journal 2021;20:995-1008 – ISSN 1611-2156
Received: April 08, 2021, accepted: May 26, 2021, published: June 01, 2021

**0.68**	1-Butanol, 3-methoxy-, acetate
**0.72**	Isotridecanol-
**0.77**	Octane, 5-ethyl-2-methyl-
**0.8**	Undecane
**0.81**	Dodecane, 4,6-dimethyl-
**0.81**	Dodecane, 4,6-dimethyl-
**0.88**	Hexasiloxane, tetradecamethyl-
**0.91**	Octane, 6-ethyl-2-methyl-
**0.92**	1-Decene, 2,4-dimethyl-
**0.92**	Cyclopentanone, 2-methyl-
**0.95**	Dodecane, 4,6-dimethyl-
**1.02**	1-Undecene, 7-methyl-
**1.02**	2-Isopropyl-5-methyl-1-heptanol
**1.02**	Tetradecane
**1.06**	Heptasiloxane, hexadecamethyl-
**1.26**	Dodecane, 4,6-dimethyl-
**1.34**	Dodecane, 4,6-dimethyl-
**1.45**	Octane, 6-ethyl-2-methyl-
**1.46**	Dodecane, 4,6-dimethyl-
**1.55**	Eicosane
**1.64**	Dodecane, 4,6-dimethyl-
**1.65**	Octane, 5-ethyl-2-methyl-
**1.66**	Dodecane, 4,6-dimethyl-
**1.83**	Dodecane, 4,6-dimethyl-
**1.9**	Dodecane, 4,6-dimethyl-
**1.93**	Dodecane, 4,6-dimethyl-
**1.98**	Eicosane
**2.14**	Eicosane
**2.63**	Undecane, 5-methyl-
**3.79**	1-Heptanol, 2,4-diethyl-
**4.06**	Dodecane
**4.22**	1-Heptanol, 2,4-diethyl-
**5.64**	Dodecane, 4,6-dimethyl-
**5.65**	1-Methoxy-2-propyl acetate
**5.87**	1-Heptanol, 2,4-diethyl-
**8.12**	Dodecane, 4,6-dimethyl-
**11.06**	1-Butanol, 3-methoxy-

EXCLI Journal 2021;20:995-1008 – ISSN 1611-2156
Received: April 08, 2021, accepted: May 26, 2021, published: June 01, 2021

Sample M
Number of peaks: 113
Number of identified compounds: 85
Ratio of identified peak area: 96.56%

Peak Area [%]	Database comparison
**0.07**	Octane, 3,6-dimethyl-
**0.07**	Pentadecane
**0.09**	Heptadecane
**0.11**	Dodecane, 4,6-dimethyl-
**0.12**	2,5-Cyclohexadiene-1,4-dione, 2,6-bis(1,1-dimethylethyl)-
**0.12**	Pentadecane, 2,6,10,14-tetramethyl-
**0.14**	Sulfurous acid, dodecyl pentyl ester
**0.15**	Decane, 1-iodo-
**0.16**	Dodecane, 3-methyl-
**0.17**	2-Undecene, 2,5-dimethyl-
**0.17**	Benzene, 3-cyclohexen-1-yl-
**0.17**	Eicosane
**0.18**	p-Xylene
**0.2**	Dodecane, 4,6-dimethyl-
**0.21**	16-Hexadecanoyl hydrazide
**0.21**	Decane, 4-ethyl-
**0.21**	Dodecane, 4,6-dimethyl-
**0.21**	Sulfurous acid, hexyl pentadecyl ester
**0.22**	2-Propenoic acid, 2-methyl-, octyl ester
**0.22**	Heptadecane, 8-methyl-
**0.24**	1,2,4-Methenoazulene, decahydro-1,5,5,8a-tetramethyl-, [1S-(1.alpha.,2.alpha.,3a.beta.,4.alpha.,8a.beta.,9R*)]-
**0.24**	1-Dodecene
**0.25**	Propanoic acid, 2-methyl-, 1-(1,1-dimethylethyl)-2-methyl-1,3-propanediyl ester
**0.26**	Hexadecane, 1-iodo-
**0.27**	3-Hexadecene, (Z)-
**0.27**	Heptadecane
**0.27**	Heptane, 2,5,5-trimethyl-
**0.28**	Undecane, 2-methyl-
**0.29**	Decane, 5-methyl-
**0.3**	Heptasiloxane, hexadecamethyl-
**0.31**	1-Hexadecanesulfonyl chloride
**0.33**	Pentadecane, 2,6,10-trimethyl-
**0.35**	Octane, 3,3-dimethyl-
**0.36**	Decane
**0.39**	1,4-Methanoazulene, decahydro-4,8,8-trimethyl-9-methylene-, [1S-(1.alpha.,3a.beta.,4.alpha.,8a.beta.)]-
**0.43**	D-Limonene
**0.43**	Eicosane

EXCLI Journal 2021;20:995-1008 – ISSN 1611-2156
Received: April 08, 2021, accepted: May 26, 2021, published: June 01, 2021

**0.46**	2-Bromo dodecane
**0.46**	Hexadecane, 2,6,11,15-tetramethyl-
**0.48**	Octane, 2,3-dimethyl-
**0.54**	Octane, 2,3,6,7-tetramethyl-
**0.55**	Heptane, 2,5,5-trimethyl-
**0.55**	Oxalic acid, propyl undecyl ester
**0.58**	Dodecane, 4,6-dimethyl-
**0.58**	Isotridecanol-
**0.6**	Dodecane, 4-methyl-
**0.62**	Dodecane, 4,6-dimethyl-
**0.68**	Tetradecane
**0.69**	Dodecane, 4,6-dimethyl-
**0.74**	Undecane, 5-methyl-
**0.78**	Undecane
**0.79**	Undecane, 3-methyl-
**0.81**	Undecane, 5-methyl-
**0.84**	Undecane, 4-methyl-
**0.88**	Dodecane, 4-methyl-
**0.97**	Octacosane
**1.1**	Dodecane, 4,6-dimethyl-
**1.11**	Dodecane, 4,6-dimethyl-
**1.18**	2-Propenoic acid, 2-ethylhexyl ester
**1.19**	Dodecane, 2,6,11-trimethyl-
**1.28**	Eicosane
**1.29**	Dodecane, 4,6-dimethyl-
**1.32**	Octane, 5-ethyl-2-methyl-
**1.38**	Dodecane, 4,6-dimethyl-
**1.4**	Dodecane, 4,6-dimethyl-
**1.6**	1-Decene, 2,4-dimethyl-
**1.63**	Eicosane
**1.69**	Nonane, 5-methyl-5-propyl-
**1.7**	Eicosane
**1.71**	2,2,4-Trimethyl-1,3-pentanediol diisobutyrate
**1.92**	1-Undecene, 7-methyl-
**2.01**	Dodecane, 4,6-dimethyl-
**2.02**	Dodecane, 4,6-dimethyl-
**2.58**	Dodecane, 4,6-dimethyl-
**2.64**	Dodecane
**2.82**	Octane, 2,3,6,7-tetramethyl-
**2.82**	Octane, 6-ethyl-2-methyl-
**3.2**	Octane, 5-ethyl-2-methyl-
**3.33**	Propanoic acid, 2-methyl-, 3-hydroxy-2,4,4-trimethylpentyl ester
**4.2**	Isotridecanol-
**4.23**	1-Heptanol, 2,4-diethyl-

EXCLI Journal 2021;20:995-1008 – ISSN 1611-2156
Received: April 08, 2021, accepted: May 26, 2021, published: June 01, 2021

**4.82**	Octane, 2,3,6,7-tetramethyl-
**5.07**	Dodecane, 4,6-dimethyl-
**5.91**	1-Heptanol, 2,4-diethyl-
**8.34**	Dodecane, 4,6-dimethyl-

EXCLI Journal 2021;20:995-1008 – ISSN 1611-2156
Received: April 08, 2021, accepted: May 26, 2021, published: June 01, 2021

Sample N
Number of peaks: 116
Number of identified compounds: 90
Ratio of identified peak area: 97.63%

Peak Area [%]	Database comparison
0.04	2,3-Dimethyldecane
0.05	Acetamide, N,N-dimethyl-
0.05	Ethanone, 1,2-diphenyl-
0.06	Benzophenone
0.07	1-Dodecene
0.07	2-Undecene, 7-methyl-
0.07	Pentatriacontane
0.08	Octane, 3,3-dimethyl-
0.09	Cyclotetrasiloxane, octamethyl-
0.1	2,5-Cyclohexadiene-1,4-dione, 2,6-bis(1,1-dimethylethyl)-
0.1	Heptadecane
0.1	Tetradecane, 5-methyl-
0.11	[1,1':3',1''-Terphenyl]-2'-ol
0.11	2-Isopropyl-5-methyl-1-heptanol
0.11	Eicosane
0.11	Tetradecane, 4-methyl-
0.12	Dichloroacetic acid, 6-ethyl-3-octyl ester
0.12	Myristic acid vinyl ester
0.13	Cyclopentasiloxane, decamethyl-
0.13	Decane, 2,3,5-trimethyl-
0.13	Heptadecane
0.13	Nonane, 5-methyl-5-propyl-
0.14	Pentadecane, 2,6,10,14-tetramethyl-
0.15	10-Methylnonadecane
0.15	1-Ethyl-2,2,6-trimethylcyclohexane
0.15	2,2,4,4-Tetramethyloctane
0.16	Phenol
0.17	Decane, 5-propyl-
0.17	Dichloroacetic acid, 6-ethyl-3-octyl ester
0.17	Eicosane
0.18	Heptadecane
0.18	p-Xylene
0.19	Decane, 2,8,8-trimethyl-
0.2	Benzaldehyde
0.2	Hexasiloxane, tetradecamethyl-
0.21	Nonacosane
0.23	Octane, 2,6,6-trimethyl-
0.26	Cycloheptasiloxane, tetradecamethyl-
0.26	Hexadecane

EXCLI Journal 2021;20:995-1008 – ISSN 1611-2156
Received: April 08, 2021, accepted: May 26, 2021, published: June 01, 2021

**0.26**	Undecane, 3-methyl-
**0.27**	Heneicosane
**0.28**	Dodecane, 4,6-dimethyl-
**0.29**	Heptasiloxane, hexadecamethyl-
**0.32**	Tetradecane
**0.37**	1-Methoxy-2-propyl acetate
**0.37**	Undecane
**0.4**	Isotridecanol-
**0.42**	Heptasiloxane, hexadecamethyl-
**0.46**	1-Octene, 3,7-dimethyl-
**0.48**	Dodecane, 4,6-dimethyl-
**0.5**	Hexadecane
**0.51**	Octane, 2,6,6-trimethyl-
**0.54**	Dodecane, 4,6-dimethyl-
**0.57**	Isotridecanol-
**0.58**	Dodecane, 4,6-dimethyl-
**0.6**	Dodecane, 4-methyl-
**0.6**	Eicosane
**0.64**	Phenol, 2,4-bis(1,1-dimethylethyl)-
**0.67**	Dodecane, 4,6-dimethyl-
**0.82**	Cyclopentanone, 2-methyl-
**0.83**	1-Hexadecanesulfonyl chloride
**0.88**	1-Butanol, 3-methoxy-, acetate
**1.03**	Dodecane, 4,6-dimethyl-
**1.08**	Dodecane, 4,6-dimethyl-
**1.22**	Dodecane, 4,6-dimethyl-
**1.26**	1-Pentadecene
**1.27**	Heptane, 2,5,5-trimethyl-
**1.32**	Eicosane
**1.34**	Dodecane, 4,6-dimethyl-
**1.37**	Caprolactam
**1.38**	Dodecane
**1.51**	Dodecane, 4,6-dimethyl-
**1.59**	Eicosane
**1.81**	Dodecane, 4,6-dimethyl-
**1.82**	Dodecane, 4,6-dimethyl-
**1.85**	Dodecane, 4,6-dimethyl-
**1.97**	Eicosane
**1.97**	Octane, 2,3,6,7-tetramethyl-
**2**	Dodecane, 4,6-dimethyl-
**2.83**	1-Octanol, 2,7-dimethyl-
**3.22**	Octane, 5-ethyl-2-methyl-
**3.27**	1-Undecene, 7-methyl-
**3.47**	Isotridecanol-
**3.62**	1-Heptanol, 2,4-diethyl-

EXCLI Journal 2021;20:995-1008 – ISSN 1611-2156
Received: April 08, 2021, accepted: May 26, 2021, published: June 01, 2021

**3.9**	Octane, 5-ethyl-2-methyl-
**4.74**	1-Heptanol, 2,4-diethyl-
**4.97**	Dodecane, 4,6-dimethyl-
**7.47**	Undecane, 5-methyl-
**8.67**	Dodecane, 4,6-dimethyl-
**8.77**	Octane, 5-ethyl-2-methyl-

EXCLI Journal 2021;20:995-1008 – ISSN 1611-2156
Received: April 08, 2021, accepted: May 26, 2021, published: June 01, 2021

Sample O
Number of peaks: 111
Number of identified compounds: 79
Ratio of identified peak area: 97.93%

Peak Area [%]	Database comparison
**0.02**	Pentadecane, 2,6,10,14-tetramethyl-
**0.03**	Pentadecane
**0.04**	1-Nonadecanol
**0.04**	2-Isopropyl-5-methyl-1-heptanol
**0.04**	Octanal
**0.05**	Heptane, 2,3,5-trimethyl-
**0.05**	Tetradecane
**0.07**	1-Dodecene
**0.07**	2,5-Cyclohexadiene-1,4-dione, 2,6-bis(1,1-dimethylethyl)-
**0.07**	Dodecanal
**0.07**	Pentadecane
**0.08**	1,6-Dioxacyclododecane-7,12-dione
**0.08**	2-Undecene, 2,5-dimethyl-
**0.09**	5,5-Dibutylnonane
**0.1**	Acetic acid, 3,7,11,15-tetramethyl-hexadecyl ester
**0.1**	Dichloroacetic acid, 6-ethyl-3-octyl ester
**0.1**	Dodecane, 4,6-dimethyl-
**0.1**	Eicosane
**0.1**	Hexadecane, 2,6,11,15-tetramethyl-
**0.11**	Decane, 3,3,8-trimethyl-
**0.11**	Dodecane, 4-methyl-
**0.12**	Eicosane
**0.13**	Octane, 2,3,6,7-tetramethyl-
**0.16**	Eicosane
**0.16**	Heneicosane
**0.17**	Caprolactam
**0.17**	Decane, 2,8,8-trimethyl-
**0.19**	1-Hexadecanesulfonyl chloride
**0.19**	Dodecane, 4,6-dimethyl-
**0.19**	Hexasiloxane, tetradecamethyl-
**0.2**	4-Decene, 7-methyl-, (E)-
**0.2**	Octane, 2,6,6-trimethyl-
**0.23**	Heptasiloxane, hexadecamethyl-
**0.24**	Tetradecane
**0.26**	1-Tridecene
**0.27**	1-Methoxy-2-propyl acetate
**0.3**	1-Butoxy-2-ethylhexane
**0.3**	Dodecane, 4,6-dimethyl-
**0.31**	Isotridecanol-
**0.32**	Dodecane, 4,6-dimethyl-

EXCLI Journal 2021;20:995-1008 – ISSN 1611-2156
Received: April 08, 2021, accepted: May 26, 2021, published: June 01, 2021

**0.35**	Dodecane, 4,6-dimethyl-
**0.36**	Dodecane, 4,6-dimethyl-
**0.38**	Heptadecane
**0.44**	Dodecane, 4-methyl-
**0.48**	1-Butanol, 3-methoxy-
**0.48**	Dodecane, 4,6-dimethyl-
**0.53**	Octacosane
**0.6**	Undecane
**0.69**	Nonane, 2,6-dimethyl-
**0.77**	Cyclooctasiloxane, hexadecamethyl-
**0.77**	Heptane, 2,5,5-trimethyl-
**0.78**	Dodecane, 4,6-dimethyl-
**0.83**	Dodecane, 4,6-dimethyl-
**0.87**	Dodecane, 4,6-dimethyl-
**0.89**	Eicosane
**0.9**	Undecane, 4-methyl-
**0.99**	Dodecane, 4,6-dimethyl-
**1.06**	Hexadecane, 2,6,11,15-tetramethyl-
**1.07**	Dodecane, 4,6-dimethyl-
**1.15**	2-Propyl-1-pentanol
**1.19**	Dodecane
**1.25**	Octane, 6-ethyl-2-methyl-
**1.27**	Dodecane, 4,6-dimethyl-
**1.39**	Cyclotetrasiloxane, octamethyl-
**1.42**	Heptadecane
**1.55**	Dodecane, 4,6-dimethyl-
**1.61**	1-Undecene, 7-methyl-
**1.74**	1-Undecene, 7-methyl-
**1.78**	Octane, 6-ethyl-2-methyl-
**2**	Isotridecanol-
**2.14**	Isotridecanol-
**3.38**	Octane, 6-ethyl-2-methyl-
**3.43**	Dodecane, 4,6-dimethyl-
**3.51**	2-Propenoic acid, 2-ethylhexyl ester
**3.61**	Octane, 6-ethyl-2-methyl-
**5.14**	Cycloheptasiloxane, tetradecamethyl-
**6.67**	Dodecane, 4,6-dimethyl-
**14.94**	Cyclopentasiloxane, decamethyl-
**19.89**	Cyclohexasiloxane, dodecamethyl-

EXCLI Journal 2021;20:995-1008 – ISSN 1611-2156
Received: April 08, 2021, accepted: May 26, 2021, published: June 01, 2021

Sample P
Number of peaks: 102
Number of identified compounds: 75
Ratio of identified peak area: 96.49%

Peak Area [%]	Database comparison
**0.09**	Octane, 2,3,6,7-tetramethyl-
**0.1**	1-Dodecene
**0.12**	Hexadecane, 2,6,10,14-tetramethyl-
**0.13**	Heptane, 2,5,5-trimethyl-
**0.14**	1-Hexadecanesulfonyl chloride
**0.16**	Nonane, 5-butyl-
**0.18**	Tetradecane, 4-methyl-
**0.2**	Dodecane, 4,6-dimethyl-
**0.22**	2-Isopropyl-5-methyl-1-heptanol
**0.23**	Tetracosane
**0.24**	2-Isopropyl-5-methyl-1-heptanol
**0.24**	2-Undecene, 2,5-dimethyl-
**0.24**	Cyclopentasiloxane, decamethyl-
**0.26**	Eicosane
**0.26**	Nonadecyl pentafluoropropionate
**0.28**	Caprolactam
**0.29**	Eicosane
**0.3**	2-Isopropyl-5-methyl-1-heptanol
**0.31**	2-Isopropyl-5-methyl-1-heptanol
**0.32**	Tetradecane, 5-methyl-
**0.33**	Octane, 5-ethyl-2-methyl-
**0.34**	Heptadecane, 8-methyl-
**0.34**	Tetradecane, 4-methyl-
**0.36**	Octane, 6-ethyl-2-methyl-
**0.38**	1-Octanol, 2,7-dimethyl-
**0.38**	Nonane, 2-methyl-5-propyl-
**0.41**	p-Xylene
**0.43**	Hexadecane, 2,6,10,14-tetramethyl-
**0.45**	Hexadecane, 1,1-bis(dodecyloxy)-
**0.46**	1-Octanol, 2-butyl-
**0.46**	Isotridecanol-
**0.48**	Hexadecane, 2,6,10,14-tetramethyl-
**0.55**	Sulfurous acid, dodecyl pentyl ester
**0.56**	Decane, 2,2-dimethyl-
**0.57**	1-Heptanol, 2,4-diethyl-
**0.6**	Heptadecane, 8-methyl-
**0.65**	Pentadecane, 2,6,10-trimethyl-
**0.66**	Octane, 5-ethyl-2-methyl-
**0.69**	Octane, 5-ethyl-2-methyl-
**0.81**	1-Methoxy-2-propyl acetate

EXCLI Journal 2021;20:995-1008 – ISSN 1611-2156
Received: April 08, 2021, accepted: May 26, 2021, published: June 01, 2021

**0.81**	Dodecane, 4,6-dimethyl-
**0.9**	Undecane
**0.92**	Dodecane, 4,6-dimethyl-
**1.04**	Heptasiloxane, hexadecamethyl-
**1.05**	Dodecane, 4,6-dimethyl-
**1.07**	Hexasiloxane, tetradecamethyl-
**1.08**	Dodecane, 4,6-dimethyl-
**1.09**	Tetradecane
**1.1**	Dodecane, 4,6-dimethyl-
**1.16**	Cyclohexanone
**1.2**	Dodecane, 4-methyl-
**1.29**	Isotridecanol-
**1.35**	Dodecane, 4,6-dimethyl-
**1.36**	Dodecane, 4,6-dimethyl-
**1.43**	Dodecane, 4,6-dimethyl-
**1.48**	Dodecane, 4,6-dimethyl-
**1.62**	Dodecane, 4,6-dimethyl-
**1.65**	1-Butanol, 3-methoxy-
**1.66**	Dodecane, 4,6-dimethyl-
**1.67**	Heptasiloxane, hexadecamethyl-
**1.71**	Isotridecanol-
**1.87**	Dodecane, 4,6-dimethyl-
**1.91**	Dodecane, 4,6-dimethyl-
**1.94**	Dodecane, 4,6-dimethyl-
**2.41**	1-Hexadecanesulfonyl chloride
**2.49**	Phenol, 2,4-bis(1,1-dimethylethyl)-
**2.58**	Dodecane
**2.77**	Eicosane
**2.83**	Eicosane
**2.96**	Eicosane
**4.8**	Dodecane, 4,6-dimethyl-
**6.6**	1-Heptanol, 2,4-diethyl-
**6.71**	Hexadecane
**7.01**	2-Isopropyl-5-methyl-1-heptanol
**8.75**	2-Isopropyl-5-methyl-1-heptanol

EXCLI Journal 2021;20:995-1008 – ISSN 1611-2156
Received: April 08, 2021, accepted: May 26, 2021, published: June 01, 2021

Sample Q
Number of peaks: 94
Number of identified compounds: 69
Ratio of identified peak area: 96.76%

Peak Area [%]	Database comparison
**0.08**	Eicosane
**0.09**	Dodecane, 4-methyl-
**0.1**	Nonane, 5-methyl-5-propyl-
**0.11**	Eicosane
**0.11**	Undecane, 2-methyl-
**0.12**	Nonadecane
**0.13**	Octanal
**0.14**	Cyclododecane
**0.16**	Dodecane, 4,6-dimethyl-
**0.16**	Dodecane, 4,6-dimethyl-
**0.17**	Dodecane, 4,6-dimethyl-
**0.17**	Octadecane
**0.18**	Acetic acid, 3,7,11,15-tetramethyl-hexadecyl ester
**0.18**	Dodecane, 2,7,10-trimethyl-
**0.19**	Dodecane, 4,6-dimethyl-
**0.2**	Nonane, 5-methyl-5-propyl-
**0.22**	Cyclotetrasiloxane, octamethyl-
**0.22**	Dodecane, 2-methyl-
**0.24**	Nonane, 5-(2-methylpropyl)-
**0.25**	Octane, 2,3,6,7-tetramethyl-
**0.27**	Cyclohexane, undecyl-
**0.29**	Decanal
**0.31**	Eicosane
**0.32**	D-Limonene
**0.32**	Tetradecane
**0.36**	2-Isopropyl-5-methyl-1-heptanol
**0.38**	Heptane, 2,5,5-trimethyl-
**0.44**	Cyclopentasiloxane, decamethyl-
**0.48**	Heptane, 2,5,5-trimethyl-
**0.49**	Dodecane, 4,6-dimethyl-
**0.49**	Ethylparaben
**0.51**	Dodecane, 4,6-dimethyl-
**0.51**	Dodecane, 4,6-dimethyl-
**0.54**	Disulfide, di-tert-dodecyl
**0.64**	2-Undecene, 2,5-dimethyl-
**0.67**	Isotridecanol-
**0.78**	2-Propenoic acid, 2-ethylhexyl ester
**0.81**	Octane, 6-ethyl-2-methyl-
**0.91**	Dodecane, 4,6-dimethyl-
**0.96**	Dodecane, 4,6-dimethyl-

**0.98**	Undecane, 4-methyl-
**1.06**	Dodecane, 4-methyl-
**1.1**	1-Hexadecanesulfonyl chloride
**1.1**	Nonane, 5-methyl-5-propyl-
**1.16**	p-Xylene
**1.17**	2-Undecene, 2,5-dimethyl-
**1.2**	Eicosane
**1.25**	Dodecane, 4,6-dimethyl-
**1.32**	Phenol, 2,4-bis(1,1-dimethylethyl)-
**1.38**	Decane
**1.42**	Dodecane, 4,6-dimethyl-
**1.47**	Eicosane
**1.56**	1-Undecene, 7-methyl-
**1.57**	Dodecane, 4,6-dimethyl-
**1.58**	Octane, 6-ethyl-2-methyl-
**1.65**	Dodecane, 4,6-dimethyl-
**1.91**	Dodecane, 4,6-dimethyl-
**2.14**	Dodecane, 4-methyl-
**2.36**	Eicosane
**2.46**	Octane, 5-ethyl-2-methyl-
**3.28**	Octane, 6-ethyl-2-methyl-
**3.93**	Isotridecanol-
**4.06**	Isotridecanol-
**5.21**	Isotridecanol-
**5.81**	Tridecane
**5.91**	Sulfurous acid, octadecyl pentyl ester
**6.87**	Dodecane, 4,6-dimethyl-
**7.04**	Undecane
**11.11**	Dodecane

Sample R
Number of peaks: 131
Number of identified compounds: 97
Ratio of identified peak area: 97.59%

Peak Area [%]	Database comparison
**0.02**	4-Octene, 2,3,6-trimethyl-
**0.03**	2-Undecene, 3-methyl-, (Z)-
**0.03**	Nonane, 2,2,4,4,6,8,8-heptamethyl-
**0.03**	Undecane, 2,5-dimethyl-
**0.04**	3-Undecene, 8-methyl-
**0.04**	Decane, 1,1'-oxybis-
**0.04**	Octacosane
**0.05**	Decane, 5,6-dimethyl-
**0.06**	Decane, 1-iodo-
**0.06**	D-Limonene
**0.06**	Nonane, 5-methyl-5-propyl-
**0.06**	Undecane, 3-methyl-
**0.07**	4-Undecene, 9-methyl-, (Z)-
**0.07**	Dodecane, 2,5-dimethyl-
**0.07**	Nonane, 5-(2-methylpropyl)-
**0.07**	Sulfurous acid, hexyl pentadecyl ester
**0.08**	Nonane, 5-methyl-5-propyl-
**0.08**	Propanoic acid, 2-methyl-, 1-(1,1-dimethylethyl)-2-methyl-1,3-propanediyl ester
**0.08**	Tetradecane, 4-methyl-
**0.09**	3-Heptene, 2,2,3,5,6-pentamethyl-
**0.09**	Dichloroacetic acid, 6-ethyl-3-octyl ester
**0.1**	Dichloroacetic acid, 6-ethyl-3-octyl ester
**0.1**	Hexadecane, 2,6,11,15-tetramethyl-
**0.1**	p-Dioxane, 2,3-bis(tert-butyloxy)-
**0.12**	1,4-Methanoazulene, decahydro-4,8,8-trimethyl-9-methylene-, [1S-(1.alpha.,3a.beta.,4.alpha.,8a.beta.)]-
**0.12**	2-Undecene, 2,5-dimethyl-
**0.12**	Benzene, 1,4-bis(1-methylethyl)-
**0.12**	Eicosane
**0.12**	Octadecane
**0.12**	p-Xylene
**0.13**	1-Decene, 3,4-dimethyl-
**0.13**	Decanal
**0.13**	n-Amylcyclohexane
**0.15**	1,4-Methanoazulene, decahydro-4,8,8-trimethyl-9-methylene-, [1S-(1.alpha.,3a.beta.,4.alpha.,8a.beta.)]-
**0.16**	Dodecane, 2,6,10-trimethyl-
**0.18**	Eicosane
**0.23**	Decane, 5-propyl-

**0.23**	Tetradecane, 4-methyl-
**0.23**	Tetradecane, 5-methyl-
**0.25**	Decyl trifluoroacetate
**0.25**	Heneicosane
**0.25**	Tetradecane, 2,5-dimethyl-
**0.29**	3-Undecene, (E)-
**0.32**	Hexadecane, 2,6,10,14-tetramethyl-
**0.35**	Dodecane, 4,6-dimethyl-
**0.35**	Tetradecane
**0.36**	1-Octanol, 2-butyl-
**0.36**	Octane, 2,3,6,7-tetramethyl-
**0.37**	Decane, 5-propyl-
**0.38**	Isotridecanol-
**0.4**	1-Hexadecanesulfonyl chloride
**0.41**	Dodecane, 4,6-dimethyl-
**0.43**	Heneicosane
**0.44**	2,2,4,4-Tetramethyloctane
**0.44**	2-Undecene, 2,5-dimethyl-
**0.45**	4-Decene, 7-methyl-, (E)-
**0.48**	Tetradecane, 2-methyl-
**0.49**	Dodecane, 4,6-dimethyl-
**0.53**	Caprolactam
**0.54**	Cyclopentasiloxane, decamethyl-
**0.55**	1-Hexanol, 2-ethyl-
**0.61**	Dodecane, 4,6-dimethyl-
**0.63**	Dodecane, 4,6-dimethyl-
**0.63**	Dodecane, 4,6-dimethyl-
**0.68**	Heptasiloxane, hexadecamethyl-
**0.69**	Dodecane, 4,6-dimethyl-
**0.71**	Hexasiloxane, tetradecamethyl-
**0.85**	Octacosane
**0.87**	Dodecane, 4,6-dimethyl-
**0.93**	Decane
**0.93**	Dodecane, 4,6-dimethyl-
**0.94**	Dodecane, 4,6-dimethyl-
**1.05**	Eicosane
**1.11**	Sulfurous acid, pentyl tridecyl ester
**1.2**	Dodecane, 4,6-dimethyl-
**1.33**	Dodecane, 4,6-dimethyl-
**1.33**	Dodecane, 4,6-dimethyl-
**1.46**	Eicosane
**1.46**	Eicosane
**1.54**	Heptane, 2,5,5-trimethyl-
**1.85**	Octane, 5-ethyl-2-methyl-
**1.86**	2-Propenoic acid, 2-ethylhexyl ester

EXCLI Journal 2021;20:995-1008 – ISSN 1611-2156
Received: April 08, 2021, accepted: May 26, 2021, published: June 01, 2021

**1.92**	Octane, 2,3,6,7-tetramethyl-
**2.48**	Isotridecanol-
**2.53**	1-Heptanol, 2,4-diethyl-
**2.83**	1-Octanol, 2,7-dimethyl-
**3.28**	1-Undecene, 7-methyl-
**3.39**	1-Heptanol, 2,4-diethyl-
**3.41**	Undecane, 5-methyl-
**3.86**	Dodecane, 4,6-dimethyl-
**4.15**	Decane, 3,3,8-trimethyl-
**4.56**	Tridecane
**4.84**	Undecane
**5.36**	Decane, 2,2-dimethyl-
**5.51**	Octane, 5-ethyl-2-methyl-
**6.83**	Octane, 5-ethyl-2-methyl-
**7.48**	Dodecane

EXCLI Journal 2021;20:995-1008 – ISSN 1611-2156
Received: April 08, 2021, accepted: May 26, 2021, published: June 01, 2021

Sample S
Number of peaks: 155
Number of identified compounds: 111
Ratio of identified peak area: 96.42%

Peak Area [%]	Database comparison
0.02	Eicosane
0.03	1-Hexadecanesulfonyl chloride
0.03	Cyclooctane, 1-methyl-3-propyl-
0.04	Dodecane, 4-methyl-
0.05	Dodecane, 4-methyl-
0.05	Pentadecane, 2,6,10,14-tetramethyl-
0.05	Tetradecanal
0.05	Tetratetracontane
0.06	10-Methylnonadecane
0.06	Dodecane, 4,6-dimethyl-
0.06	Heneicosane
0.06	Octacosyl trifluoroacetate
0.06	Pentatriacontane
0.06	Tridecane, 5-methyl-
0.06	Tridecane, 5-methyl-
0.06	Undecane, 3,7-dimethyl-
0.07	Hexadecane, 2-methyl-
0.08	Cyclooctane, (1-methylpropyl)-
0.08	Decanal
0.08	Tetradecane, 4-methyl-
0.09	Heneicosane
0.09	Octadecane
0.1	Oxalic acid, 2-ethylhexyl tetradecyl ester
0.11	Decane
0.11	Heptadecane
0.11	Hexadecane, 2,6,10,14-tetramethyl-
0.13	Eicosane
0.14	Hexadecane
0.14	Undecane, 2-methyl-
0.14	Undecane, 4,6-dimethyl-
0.15	Dodecane, 4,6-dimethyl-
0.16	p-Xylene
0.17	Hexyl octyl ether
0.17	Silane, cyclohexyldimethoxymethyl-
0.18	1,3-Dioxolane-2-acetic acid, 2,4-dimethyl-, ethyl ester
0.18	Cycloheptasiloxane, tetradecamethyl-
0.19	Heneicosane
0.19	Heptasiloxane, hexadecamethyl-
0.2	Nonane, 5-propyl-
0.21	Nonane, 2,5-dimethyl-

**0.23**	Decane, 2,8,8-trimethyl-
**0.23**	Dodecane, 2-methyl-
**0.23**	Dodecane, 4,6-dimethyl-
**0.24**	Heneicosane
**0.24**	Tetradecane
**0.25**	Decane, 4-methyl-
**0.25**	Heneicosane
**0.25**	Octane, 3,3-dimethyl-
**0.26**	1-Hexadecanesulfonyl chloride
**0.26**	1-Tridecene
**0.26**	Dodecane, 4,6-dimethyl-
**0.26**	Undecane, 2-methyl-
**0.28**	1-Hexanol, 3,5,5-trimethyl-
**0.28**	Undecane, 5-methyl-
**0.31**	Nonane, 5-(2-methylpropyl)-
**0.33**	Decane, 3,4-dimethyl-
**0.33**	Nonane, 5-butyl-
**0.34**	1-Undecene, 7-methyl-
**0.34**	Nonane, 4,5-dimethyl-
**0.35**	Dodecane, 4-methyl-
**0.35**	Dodecane, 4-methyl-
**0.36**	2-Undecene, 2,5-dimethyl-
**0.37**	Octane, 2,5,6-trimethyl-
**0.38**	2,3-Dimethyldodecane
**0.38**	2-Undecene, 4,5-dimethyl-, [R*,R*-(E)]-
**0.38**	Propanoic acid, 2-hydroxy-2-methyl-, methyl ester
**0.42**	Caprolactam
**0.42**	Heptadecane
**0.42**	Isotridecanol-
**0.43**	Dodecane, 4,6-dimethyl-
**0.43**	Hexadecane, 2,6,11,15-tetramethyl-
**0.45**	Dodecane
**0.47**	Dodecane, 4,6-dimethyl-
**0.49**	Sulfurous acid, nonyl pentyl ester
**0.5**	Nonane, 5-(2-methylpropyl)-
**0.51**	Dodecane, 4,6-dimethyl-
**0.57**	Nonane, 5-(2-methylpropyl)-
**0.59**	Dodecane, 4,6-dimethyl-
**0.61**	1-Hexadecanesulfonyl chloride
**0.68**	Octane, 2,3,6,7-tetramethyl-
**0.78**	Dodecane, 4,6-dimethyl-
**0.78**	Octacosane
**0.79**	Dodecane, 4,6-dimethyl-
**0.81**	4-Decene, 7-methyl-, (E)-
**0.86**	Eicosane

EXCLI Journal 2021;20:995-1008 – ISSN 1611-2156
Received: April 08, 2021, accepted: May 26, 2021, published: June 01, 2021

**0.88**	Dodecane, 4,6-dimethyl-
**0.9**	Dodecane, 4,6-dimethyl-
**0.91**	Dodecane, 4,6-dimethyl-
**0.91**	Heneicosane
**0.95**	Dodecane, 4,6-dimethyl-
**1.17**	Heptasiloxane, hexadecamethyl-
**1.36**	Dodecane, 4,6-dimethyl-
**1.79**	Dodecane, 4,6-dimethyl-
**1.95**	Hexasiloxane, tetradecamethyl-
**2.07**	1-Methoxy-2-propyl acetate
**2.1**	Acetic acid, butyl ester
**2.32**	Dodecane, 4,6-dimethyl-
**2.34**	Octane, 5-ethyl-2-methyl-
**2.59**	Dodecane, 4,6-dimethyl-
**2.72**	Octane, 2,6,6-trimethyl-
**3.08**	2-Isopropyl-5-methyl-1-heptanol
**3.14**	Octane, 6-ethyl-2-methyl-
**3.25**	1-Heptanol, 2,4-diethyl-
**3.67**	Heptane, 2,5,5-trimethyl-
**3.95**	Dodecane, 4,6-dimethyl-
**4.03**	Heptane, 2,5,5-trimethyl-
**4.63**	Isotridecanol-
**5.51**	Octane, 5-ethyl-2-methyl-
**5.57**	1-Undecene, 7-methyl-
**5.62**	Octane, 5-ethyl-2-methyl-
**6.19**	1-Undecene, 7-methyl-

EXCLI Journal 2021;20:995-1008 – ISSN 1611-2156
Received: April 08, 2021, accepted: May 26, 2021, published: June 01, 2021

Sample T
Number of peaks: 129
Number of identified compounds: 88
Ratio of identified peak area: 95.04%

Peak Area [%]	Database comparison
**0.02**	Benzene, butyl-
**0.04**	.beta.-Pinene
**0.04**	2-Bromotetradecane
**0.04**	2-Bromotetradecane
**0.08**	4-Decene, 7-methyl-, (E)-
**0.08**	Heptadecane
**0.08**	Pentadecane, 2,6,10,14-tetramethyl-
**0.08**	Tetradecane, 5-methyl-
**0.09**	Tetradecane, 4-methyl-
**0.1**	1-Heptanol, 6-methyl-
**0.1**	Eicosane
**0.1**	Isotridecanol-
**0.11**	Dodecane
**0.12**	Benzene, 1-ethyl-3-(1-methylethyl)-
**0.12**	Decane, 5-propyl-
**0.12**	Hexadecane, 1-(ethenyloxy)-
**0.14**	Heptadecane, 2,6,10,15-tetramethyl-
**0.15**	1-Butanol, 3-methoxy-
**0.15**	Dodecane, 4,6-dimethyl-
**0.15**	Hexadecane, 2,6,10,14-tetramethyl-
**0.16**	Benzene, 1-methyl-4-(1-methylpropyl)-
**0.17**	Benzene, 1-methyl-4-(1-methylpropyl)-
**0.17**	Benzene, pentamethyl-
**0.18**	1-Hexadecanesulfonyl chloride
**0.18**	Benzene, 1-ethyl-2,4-dimethyl-
**0.19**	Decane, 2,8,8-trimethyl-
**0.19**	Eicosane
**0.2**	1-Methoxy-2-propyl acetate
**0.2**	Isotridecanol-
**0.22**	Heneicosane
**0.22**	Pentanedioic acid, dimethyl ester
**0.23**	Benzene, 1,4-diethyl-2-methyl-
**0.23**	Benzene, 1-ethyl-4-(1-methylethyl)-
**0.24**	Dodecane, 4,6-dimethyl-
**0.25**	Heptane, 2,5,5-trimethyl-
**0.25**	Hexanedioic acid, dimethyl ester
**0.27**	Dodecane, 4,6-dimethyl-
**0.27**	Heptane, 2,5,5-trimethyl-
**0.28**	Benzene, 1-methyl-4-propyl-
**0.29**	Dodecane, 4,6-dimethyl-

EXCLI Journal 2021;20:995-1008 – ISSN 1611-2156
Received: April 08, 2021, accepted: May 26, 2021, published: June 01, 2021

**0.31**	Dodecane, 4,6-dimethyl-
**0.31**	Nonane, 5-(1-methylpropyl)-
**0.35**	Tetradecane, 4-methyl-
**0.36**	Dodecane, 4,6-dimethyl-
**0.37**	Nonane, 5-methyl-5-propyl-
**0.42**	p-Xylene
**0.46**	Hexadecane, 2,6,11,15-tetramethyl-
**0.48**	Hexasiloxane, tetradecamethyl-
**0.49**	Cyclotetrasiloxane, octamethyl-
**0.52**	Dodecane, 4,6-dimethyl-
**0.52**	Heptasiloxane, hexadecamethyl-
**0.53**	Eicosane
**0.54**	1-Decene, 2,4-dimethyl-
**0.54**	Hexadecane
**0.55**	Benzene, 1-ethyl-2,4,5-trimethyl-
**0.58**	Dodecane, 4,6-dimethyl-
**0.67**	Benzene, 1,2,3-trimethyl-
**0.69**	1H-Indene, 2,3-dihydro-5-methyl-
**0.69**	Cyclopentasiloxane, decamethyl-
**0.74**	Benzene, 1-ethyl-2,4,5-trimethyl-
**0.77**	1-Tetradecene
**0.77**	Benzene, 1-ethyl-2,3-dimethyl-
**0.78**	1-Undecene, 7-methyl-
**0.8**	Dodecane, 4,6-dimethyl-
**0.82**	Benzene, 1-methyl-2-propyl-
**0.94**	Isotridecanol-
**1.07**	Eicosane
**1.08**	Dodecane, 4,6-dimethyl-
**1.1**	Isotridecanol-
**1.19**	Benzene, 1-ethyl-2,4,5-trimethyl-
**1.19**	Dodecane, 4,6-dimethyl-
**1.5**	1H-Indene, 2,3-dihydro-5-methyl-
**1.62**	Isotridecanol-
**1.69**	Dodecane, 4,6-dimethyl-
**1.95**	Dodecane, 4,6-dimethyl-
**1.98**	Benzene, 1-ethyl-3,5-dimethyl-
**2.06**	Heneicosane
**2.21**	Benzene, 1-ethyl-3,5-dimethyl-
**2.21**	Octane, 6-ethyl-2-methyl-
**2.89**	Benzene, 1,2,3,5-tetramethyl-
**3.03**	Benzene, 1,2,3,4-tetramethyl-
**3.14**	Octane, 6-ethyl-2-methyl-
**4.46**	Octane, 6-ethyl-2-methyl-
**4.76**	Nonane, 5-methyl-5-propyl-
**6.86**	Benzene, 1,2,4,5-tetramethyl-

EXCLI Journal 2021;20:995-1008 – ISSN 1611-2156
Received: April 08, 2021, accepted: May 26, 2021, published: June 01, 2021

**8.42**	Hexadecane
**9.45**	2-Cyclohexen-1-one, 3,5,5-trimethyl-
**9.88**	Octane, 5-ethyl-2-methyl-

EXCLI Journal 2021;20:995-1008 – ISSN 1611-2156
Received: April 08, 2021, accepted: May 26, 2021, published: June 01, 2021

Sample U
Number of peaks: 117
Number of identified compounds: 83
Ratio of identified peak area: 96,4%

Peak Area [%]	Database comparison
**0.07**	Undecane, 3,8-dimethyl-
**0.08**	Eicosane
**0.1**	Octadecane
**0.1**	Tetracosane
**0.1**	Undecane, 5-methyl-
**0.11**	Dodecane, 4,6-dimethyl-
**0.12**	Nonane, 5-methyl-5-propyl-
**0.13**	Decane, 3-ethyl-3-methyl-
**0.14**	Octacosane
**0.14**	Undecane, 3-methyl-
**0.16**	Undecane, 3,9-dimethyl-
**0.18**	4-Decene, 7-methyl-, (E)-
**0.18**	Caprolactam
**0.2**	Tetracosane
**0.21**	Dodecane, 2,6,10-trimethyl-
**0.22**	Heptacosane
**0.22**	Hexadecane
**0.24**	2-Undecene, 4,5-dimethyl-, [R*,R*-(E)]-
**0.25**	Benzene, 1,3-bis(1-methylethyl)-
**0.26**	Dodecane, 4,6-dimethyl-
**0.26**	Nonane, 3-methyl-5-propyl-
**0.27**	Cyclopentasiloxane, decamethyl-
**0.27**	Hexadecane, 2,6,10,14-tetramethyl-
**0.28**	Octane, 2,3,6,7-tetramethyl-
**0.28**	Pentafluoropropionic acid, undecyl ester
**0.29**	Hexadecane
**0.31**	2-Isopropyl-5-methyl-1-heptanol
**0.31**	Decane, 2,8,8-trimethyl-
**0.31**	Heneicosane
**0.33**	Cyclopentane, decyl-
**0.33**	Octadecane
**0.35**	2-Isopropyl-5-methyl-1-heptanol
**0.37**	1-Hexadecanesulfonyl chloride
**0.39**	Hexadecane
**0.41**	Eicosane
**0.43**	2-Bromo dodecane
**0.44**	Dodecane, 4,6-dimethyl-
**0.46**	p-Xylene
**0.5**	Hexadecane, 1,1-bis(dodecyloxy)-
**0.57**	1-Methoxy-2-propyl acetate

EXCLI Journal 2021;20:995-1008 – ISSN 1611-2156
Received: April 08, 2021, accepted: May 26, 2021, published: June 01, 2021

**0.57**	Disulfide, di-tert-dodecyl
**0.57**	Hexasiloxane, tetradecamethyl-
**0.58**	Heptane, 2,5,5-trimethyl-
**0.63**	Isotridecanol-
**0.64**	2,2,4,4-Tetramethyloctane
**0.65**	Eicosane
**0.68**	Undecane
**0.71**	Octane, 5-ethyl-2-methyl-
**0.77**	Dodecane, 4,6-dimethyl-
**0.78**	Dodecane, 4,6-dimethyl-
**0.83**	Heptasiloxane, hexadecamethyl-
**0.88**	1-Butanol, 3-methoxy-, acetate
**0.91**	Dodecane, 4,6-dimethyl-
**1**	Dodecane, 4,6-dimethyl-
**1.01**	Dodecane, 4,6-dimethyl-
**1.05**	Cyclopentanone, 2-methyl-
**1.06**	Octacosane
**1.12**	Dodecane, 4,6-dimethyl-
**1.26**	Heptasiloxane, hexadecamethyl-
**1.32**	Dodecane, 4,6-dimethyl-
**1.36**	Dodecane, 4,6-dimethyl-
**1.47**	Eicosane
**1.48**	Dodecane, 4,6-dimethyl-
**1.51**	Undecane, 4-methyl-
**1.67**	Octane, 5-ethyl-2-methyl-
**1.67**	Tetradecane
**1.84**	Eicosane
**1.94**	2-Undecene, 2,5-dimethyl-
**1.95**	Dodecane, 4,6-dimethyl-
**1.97**	Dodecane, 4,6-dimethyl-
**2.01**	Dodecane, 4,6-dimethyl-
**2.04**	Octane, 5-ethyl-2-methyl-
**2.07**	1-Undecene, 7-methyl-
**2.32**	Dodecane, 4,6-dimethyl-
**2.57**	Eicosane
**3.24**	Octane, 5-ethyl-2-methyl-
**3.39**	Decane, 2,2-dimethyl-
**3.94**	Dodecane
**4.83**	Isotridecanol-
**5.19**	1-Heptanol, 2,4-diethyl-
**5.76**	Dodecane, 4,6-dimethyl-
**6.88**	1-Heptanol, 2,4-diethyl-
**7.91**	Dodecane, 4,6-dimethyl-

EXCLI Journal 2021;20:995-1008 – ISSN 1611-2156
Received: April 08, 2021, accepted: May 26, 2021, published: June 01, 2021

Sample V
Number of peaks: 131
Number of identified compounds: 88
Ratio of identified peak area: 95.40%

Peak Area [%]	Database comparison
**0.05**	Undecane, 2-methyl-
**0.06**	Cyclododecane
**0.07**	Undecane, 2,9-dimethyl-
**0.09**	Hexadecane, 1-iodo-
**0.11**	1-Undecene, 4-methyl-
**0.11**	Cyclopentasiloxane, decamethyl-
**0.11**	Decane, 4-methyl-
**0.11**	Dodecane
**0.11**	Heptane, 2,5,5-trimethyl-
**0.11**	Oxalic acid, 2-ethylhexyl hexyl ester
**0.12**	Hexadecane, 2,6,10,14-tetramethyl-
**0.13**	1-Heptanol, 2,4-diethyl-
**0.14**	Tetradecane, 5-methyl-
**0.15**	2-Isopropyl-5-methyl-1-heptanol
**0.15**	Silane, cyclohexyldimethoxymethyl-
**0.16**	Decane, 3,3,5-trimethyl-
**0.16**	Hexadecane, 2-methyl-
**0.16**	Nonacosane
**0.17**	2,2,4,4-Tetramethyloctane
**0.17**	2,3-Dimethyldodecane
**0.18**	Hexadecane, 2,6,10,14-tetramethyl-
**0.19**	Dichloroacetic acid, 6-ethyl-3-octyl ester
**0.19**	Hexadecane
**0.19**	p-Xylene
**0.2**	Pentadecane, 4-methyl-
**0.2**	Sulfurous acid, dodecyl pentyl ester
**0.21**	Acetamide, N,N-dimethyl-
**0.24**	Dodecane, 4,6-dimethyl-
**0.26**	2-Isopropyl-5-methyl-1-heptanol
**0.26**	Tetradecane, 2,5-dimethyl-
**0.26**	Tetradecane, 5-methyl-
**0.27**	Heptadecane
**0.27**	Nonane, 5-(2-methylpropyl)-
**0.28**	Tetradecane, 4-methyl-
**0.29**	Heneicosane
**0.29**	Tetradecane, 2-methyl-
**0.31**	Sulfurous acid, pentyl undecyl ester
**0.34**	Hexadecane, 2,6,10,14-tetramethyl-
**0.34**	Phenol, 2,5-bis(1,1-dimethylethyl)-
**0.35**	Dodecane, 4,6-dimethyl-

EXCLI Journal 2021;20:995-1008 – ISSN 1611-2156
Received: April 08, 2021, accepted: May 26, 2021, published: June 01, 2021

**0.37**	3-Hexadecene, (Z)-
**0.38**	1-Heptanol, 2,4-diethyl-
**0.38**	Pentadecane, 4-methyl-
**0.39**	Isotridecanol-
**0.4**	Nonane, 3-methyl-5-propyl-
**0.4**	Pentadecane, 2,6,10,14-tetramethyl-
**0.44**	Octane, 2,6,6-trimethyl-
**0.45**	Hexadecane, 2,6,10,14-tetramethyl-
**0.46**	Dodecane, 4,6-dimethyl-
**0.46**	Heptasiloxane, hexadecamethyl-
**0.46**	Nonane, 5-methyl-5-propyl-
**0.47**	Decane, 2,8,8-trimethyl-
**0.5**	2-Decene, 8-methyl-, (Z)-
**0.54**	Decane, 5-propyl-
**0.56**	Tetradecane
**0.58**	Dodecane, 4,6-dimethyl-
**0.76**	Dodecane, 4-methyl-
**0.78**	Dodecane, 4,6-dimethyl-
**0.79**	Heptadecane
**0.8**	Pentadecane, 8-hexyl-
**0.81**	4-Decene, 7-methyl-, (E)-
**0.94**	Decane
**0.97**	Dodecane, 4,6-dimethyl-
**1.01**	Octacosane
**1.03**	Hexasiloxane, tetradecamethyl-
**1.06**	Dodecane, 4,6-dimethyl-
**1.08**	Dodecane
**1.11**	Heptasiloxane, hexadecamethyl-
**1.21**	Eicosane
**1.36**	Octane, 5-ethyl-2-methyl-
**1.74**	Dodecane, 4,6-dimethyl-
**2.11**	Octane, 2,6,6-trimethyl-
**2.2**	Dodecane, 4,6-dimethyl-
**2.24**	Caprolactam
**2.33**	Dodecane, 4,6-dimethyl-
**2.84**	Heptane, 2,5,5-trimethyl-
**2.92**	Hexadecane
**3.22**	Undecane
**3.32**	Dodecane, 4,6-dimethyl-
**3.39**	Undecane, 5-methyl-
**3.86**	Octane, 5-ethyl-2-methyl-
**4.11**	Isotridecanol-
**4.22**	Isotridecanol-
**4.43**	Dodecane, 4,6-dimethyl-
**5.06**	1-Decene, 2,4-dimethyl-

EXCLI Journal 2021;20:995-1008 – ISSN 1611-2156
Received: April 08, 2021, accepted: May 26, 2021, published: June 01, 2021

**5.71**	1-Undecene, 7-methyl-
**5.94**	2-Isopropyl-5-methyl-1-heptanol
**7.25**	Octane, 5-ethyl-2-methyl-

Sample W
Number of peaks: 130
Number of identified compounds: 92
Ratio of identified peak area: 97.63%

Peak Area [%]	Database comparison
**0.02**	Cycloheptasiloxane, tetradecamethyl-
**0.02**	Hexadecane, 1-iodo-
**0.03**	Decane, 5,6-dimethyl-
**0.03**	Eicosane
**0.03**	Nonane, 5-(2-methylpropyl)-
**0.04**	1-Undecene, 9-methyl-
**0.04**	Cyclohexane, (1,3-dimethylbutyl)-
**0.05**	Cyclopentane, 1-butyl-2-propyl-
**0.05**	Dodecyl trifluoroacetate
**0.05**	Nonane, 5-(2-methylpropyl)-
**0.05**	Nonane, 5-butyl-
**0.05**	Undecane, 3-methyl-
**0.07**	1-Decanol, 2-hexyl-
**0.07**	1-Decene, 3,4-dimethyl-
**0.07**	1-Undecene, 7-methyl-
**0.07**	Decanal
**0.07**	Eicosane
**0.08**	2-Undecene, 2,5-dimethyl-
**0.08**	Hexadecane, 2,6,11,15-tetramethyl-
**0.08**	Hexadecane, 2,6,11,15-tetramethyl-
**0.08**	Pentafluoropropionic acid, pentadecyl ester
**0.09**	Hexadecane
**0.1**	Tetracosane
**0.11**	2-Propenoic acid, 2-methyl-, octyl ester
**0.11**	2-Undecene, 2,5-dimethyl-
**0.11**	Eicosane
**0.12**	p-Xylene
**0.13**	2,5-Cyclohexadiene-1,4-dione, 2,6-bis(1,1-dimethylethyl)-
**0.13**	2-Isopropyl-5-methyl-1-heptanol
**0.13**	Cyclotetrasiloxane, octamethyl-
**0.13**	Eicosane
**0.15**	p-Dioxane, 2,3-bis(tert-butyloxy)-
**0.15**	Tetradecane
**0.15**	Undecane, 3,6-dimethyl-
**0.16**	Heneicosane
**0.16**	Hexadecane
**0.19**	Heneicosane
**0.2**	Decane, 2,8,8-trimethyl-
**0.21**	Eicosane
**0.22**	1-Decene, 3,3,4-trimethyl-

EXCLI Journal 2021;20:995-1008 – ISSN 1611-2156
Received: April 08, 2021, accepted: May 26, 2021, published: June 01, 2021

**0.23**	Dodecane, 4,6-dimethyl-
**0.24**	Nonane, 5-methyl-5-propyl-
**0.25**	Octane, 2,6,6-trimethyl-
**0.27**	Cyclopentasiloxane, decamethyl-
**0.3**	Dodecane, 2,6,10-trimethyl-
**0.31**	1-Hexadecanesulfonyl chloride
**0.31**	Caprolactam
**0.33**	4-Decene, 7-methyl-, (E)-
**0.34**	Eicosane
**0.38**	Hexadecane, 2,6,11,15-tetramethyl-
**0.42**	Dodecane, 4,6-dimethyl-
**0.46**	Cyclohexanone
**0.46**	Dodecane, 4,6-dimethyl-
**0.46**	Dodecane, 4,6-dimethyl-
**0.49**	1-Methoxy-2-propyl acetate
**0.49**	Isotridecanol-
**0.55**	2-Propenoic acid, 2-ethylhexyl ester
**0.59**	Dodecane, 4-methyl-
**0.64**	Eicosane
**0.67**	Dodecane, 4,6-dimethyl-
**0.78**	1-Hexadecanesulfonyl chloride
**0.82**	Dodecane, 4,6-dimethyl-
**0.89**	Heptasiloxane, hexadecamethyl-
**1.01**	Dodecane, 4,6-dimethyl-
**1.09**	Dodecane, 4,6-dimethyl-
**1.11**	Dodecane, 4,6-dimethyl-
**1.15**	2,2,4,4-Tetramethyloctane
**1.15**	Octane, 3,3-dimethyl-
**1.29**	Eicosane
**1.31**	Dodecane, 4,6-dimethyl-
**1.34**	Dodecane, 4,6-dimethyl-
**1.46**	1,3-Dioxan-5-ol, 4,4,5-trimethyl-
**1.53**	Octane, 5-ethyl-2-methyl-
**1.54**	Dodecane, 4,6-dimethyl-
**1.71**	Undecane, 4-methyl-
**1.75**	Dodecane, 4,6-dimethyl-
**1.8**	Hexasiloxane, tetradecamethyl-
**1.84**	Eicosane
**2.44**	Octane, 5-ethyl-2-methyl-
**2.52**	Eicosane
**2.58**	1-Undecene, 7-methyl-
**2.92**	1-Undecene, 7-methyl-
**2.92**	Undecane
**3.53**	1-Heptanol, 2,4-diethyl-
**3.53**	Isotridecanol-

EXCLI Journal 2021;20:995-1008 – ISSN 1611-2156
Received: April 08, 2021, accepted: May 26, 2021, published: June 01, 2021

**4.06**	Dodecane
**4.22**	Undecane, 5-methyl-
**4.49**	1-Heptanol, 2,4-diethyl-
**4.67**	Dodecane, 4,6-dimethyl-
**4.89**	Octane, 5-ethyl-2-methyl-
**8.44**	Dodecane, 4,6-dimethyl-
**10.78**	Decane, 2,2-dimethyl-

EXCLI Journal 2021;20:995-1008 – ISSN 1611-2156
Received: April 08, 2021, accepted: May 26, 2021, published: June 01, 2021

Sample X
Number of peaks: 111
Number of identified compounds: 81
Ratio of identified peak area: 98.11%

Peak Area [%]	Database comparison
**0.05**	Cyclooctane, (1-methylpropyl)-
**0.05**	Dodecane, 2,6,11-trimethyl-
**0.07**	1-Undecene, 7-methyl-
**0.07**	Cyclopentasiloxane, decamethyl-
**0.07**	Eicosane
**0.07**	Isodecyl methacrylate
**0.08**	Decanal
**0.09**	2,2,4,4-Tetramethyloctane
**0.09**	Decane
**0.09**	p-Dioxane, 2,3-bis(tert-butyloxy)-
**0.09**	Tetradecane, 5-methyl-
**0.09**	Undecane
**0.1**	Heneicosane
**0.1**	Heptadecane
**0.1**	Hexadecane
**0.1**	Tetradecane
**0.1**	Tridecane, 5-propyl-
**0.11**	Ethanol, 2-(1,1-dimethylethoxy)-
**0.12**	1-Methoxy-2-propyl acetate
**0.12**	1-Undecene, 4-methyl-
**0.12**	Acetic acid, butyl ester
**0.12**	Tetradecane, 4-methyl-
**0.13**	Octane, 2,6,6-trimethyl-
**0.14**	4-Butoxy-2-butanone
**0.14**	Decane, 2,8,8-trimethyl-
**0.14**	Pentadecane, 2,6,10,14-tetramethyl-
**0.15**	Dodecane, 4,6-dimethyl-
**0.15**	Pentadecane
**0.17**	Hexadecane, 1-iodo-
**0.18**	Benzene, (1-methylethyl)-
**0.21**	2-Undecene, 2,5-dimethyl-
**0.21**	Tetradecane
**0.22**	Tridecane, 6-methyl-
**0.23**	Octane, 2,4,6-trimethyl-
**0.25**	1,1,4-Trimethylcyclohexane
**0.26**	Cycloheptasiloxane, tetradecamethyl-
**0.29**	Octane, 2,6,6-trimethyl-
**0.3**	Dodecane, 4,6-dimethyl-
**0.3**	Dodecane, 4,6-dimethyl-
**0.3**	Dodecane, 4,6-dimethyl-

EXCLI Journal 2021;20:995-1008 – ISSN 1611-2156
Received: April 08, 2021, accepted: May 26, 2021, published: June 01, 2021

**0.31**	Phenol, 2,4-bis(1,1-dimethylethyl)-
**0.32**	Eicosane
**0.37**	Isotridecanol-
**0.42**	Heptadecane
**0.43**	Cyclopentanone, 2-methyl-
**0.46**	Dodecane
**0.54**	Hexasiloxane, tetradecamethyl-
**0.55**	Heptasiloxane, hexadecamethyl-
**0.57**	Dodecane, 4-methyl-
**0.62**	Tetradecyl trifluoroacetate
**0.64**	Dodecane, 4,6-dimethyl-
**0.73**	4-Decene, 7-methyl-, (E)-
**0.73**	Dodecane, 4,6-dimethyl-
**0.78**	2-Isopropyl-5-methyl-1-heptanol
**0.8**	Eicosane
**0.86**	Dodecane, 4,6-dimethyl-
**0.9**	Eicosane
**0.96**	Heneicosane
**0.98**	Dodecane, 4,6-dimethyl-
**1.22**	Dodecane, 4,6-dimethyl-
**1.28**	Dodecane, 4,6-dimethyl-
**1.36**	Dodecane, 4,6-dimethyl-
**1.37**	Dodecane, 4,6-dimethyl-
**1.59**	Dodecane, 4,6-dimethyl-
**2.08**	Acetamide, N,N-dimethyl-
**2.15**	Octane, 2,3,6,7-tetramethyl-
**2.45**	Heptane, 2,5,5-trimethyl-
**2.53**	Isotridecanol-
**2.64**	1-Heptanol, 2,4-diethyl-
**3.28**	Dodecane, 4,6-dimethyl-
**3.39**	Nonane, 2,6-dimethyl-
**3.56**	1-Undecene, 7-methyl-
**3.79**	1-Heptanol, 2,4-diethyl-
**3.98**	Octane, 5-ethyl-2-methyl-
**4.19**	1-Undecene, 7-methyl-
**4.39**	Caprolactam
**4.53**	Nonane, 2,6-dimethyl-
**5.49**	p-Xylene
**5.78**	Dodecane, 4,6-dimethyl-
**8.11**	Octane, 5-ethyl-2-methyl-
**11.21**	Octane, 5-ethyl-2-methyl-

Sample Y
Number of peaks: 105
Number of identified compounds: 73
Ratio of identified peak area: 94.53%

Peak Area [%]	Database comparison
**0.08**	Decane, 2,3,8-trimethyl-
**0.09**	Dodecane, 2,6,10-trimethyl-
**0.09**	Oxygen
**0.14**	2-Undecene, 2,5-dimethyl-
**0.14**	Tetratetracontane
**0.15**	2-Undecene, 7-methyl-
**0.16**	Hexadecane, 2-methyl-
**0.16**	Tetradecane, 4-methyl-
**0.2**	Nonane, 5-butyl-
**0.2**	Tetradecane, 4-methyl-
**0.21**	2-Isopropyl-5-methyl-1-heptanol
**0.21**	2-Undecene, 4,5-dimethyl-, [R*,R*-(E)]-
**0.23**	2,5-Cyclohexadiene-1,4-dione, 2,6-bis(1,1-dimethylethyl)-
**0.23**	Nonane, 5-(2-methylpropyl)-
**0.24**	Trichloroacetic acid, hexadecyl ester
**0.25**	1-Hexanol, 2-ethyl-
**0.25**	Pentadecane, 2,6,10,14-tetramethyl-
**0.26**	Decane, 2,2-dimethyl-
**0.26**	Decane, 5-propyl-
**0.26**	Hexadecane, 2,6,11,15-tetramethyl-
**0.28**	Caprolactam
**0.28**	Hexadecane
**0.3**	Undecane, 4,7-dimethyl-
**0.35**	1-Octanol, 2-butyl-
**0.38**	Eicosane
**0.4**	Decanal
**0.4**	Pentadecane, 2,6,10,14-tetramethyl-
**0.42**	1-Tricosene
**0.42**	Cyclopentasiloxane, decamethyl-
**0.43**	Octane, 6-ethyl-2-methyl-
**0.44**	Dodecane, 4,6-dimethyl-
**0.47**	Decane, 2,8,8-trimethyl-
**0.49**	Undecane
**0.5**	Decane, 3,3,8-trimethyl-
**0.5**	Dodecane, 4,6-dimethyl-
**0.53**	Dodecane, 4,6-dimethyl-
**0.6**	Octane, 2,3,6,7-tetramethyl-
**0.61**	Pentadecane, 2,6,10-trimethyl-
**0.63**	1-Methoxy-2-propyl acetate
**0.76**	Dodecane, 4,6-dimethyl-

EXCLI Journal 2021;20:995-1008 – ISSN 1611-2156
Received: April 08, 2021, accepted: May 26, 2021, published: June 01, 2021

**0.76**	Eicosane
**0.77**	Eicosane
**0.8**	Sulfurous acid, dodecyl pentyl ester
**0.82**	Eicosane
**0.89**	Dodecane, 4-methyl-
**0.9**	Dodecane, 4,6-dimethyl-
**0.92**	Dodecane, 4,6-dimethyl-
**0.92**	Dodecane, 4,6-dimethyl-
**0.98**	Isotridecanol-
**1**	1-Hexadecanesulfonyl chloride
**1.03**	Tetradecane
**1.11**	1-Butanol, 3-methoxy-
**1.12**	Octane, 5-ethyl-2-methyl-
**1.13**	Hexadecane
**1.19**	Dodecane, 4,6-dimethyl-
**1.21**	Heptasiloxane, hexadecamethyl-
**1.44**	Sulfurous acid, pentyl tridecyl ester
**1.71**	Dodecane, 4,6-dimethyl-
**1.8**	2-Isopropyl-5-methyl-1-heptanol
**1.83**	Eicosane
**1.86**	Dodecane, 4,6-dimethyl-
**1.87**	Dodecane
**2.27**	Eicosane
**2.5**	Dodecane, 4,6-dimethyl-
**2.67**	Dodecane, 4,6-dimethyl-
**2.72**	Dodecane, 4,6-dimethyl-
**3.43**	Isotridecanol-
**3.89**	Isotridecanol-
**3.96**	Eicosane
**4.62**	Isotridecanol-
**7.21**	Dodecane, 4,6-dimethyl-
**7.73**	Eicosane
**14.47**	Dodecane, 4,6-dimethyl-

Sample Z
Number of peaks: 146
Number of identified compounds: 98
Ratio of identified peak area: 96.61%

Peak Area [%]	Database comparison
**0.04**	Dodecane, 4-methyl-
**0.04**	Tridecane, 4-methyl-
**0.06**	Octanal
**0.06**	Undecane
**0.07**	1-Heptanol, 2,4-diethyl-
**0.07**	1-Undecene, 7-methyl-
**0.07**	Octacosane
**0.07**	Octane, 2,4,6-trimethyl-
**0.08**	Decane, 4-ethyl-
**0.08**	Undecane, 2-methyl-
**0.09**	Cyclododecane
**0.09**	Cyclotetrasiloxane, octamethyl-
**0.09**	Decane, 5,6-dimethyl-
**0.1**	Decane, 4-methyl-
**0.11**	Hexadecane, 2,6,11,15-tetramethyl-
**0.11**	Pentadecane, 7-methyl-
**0.12**	Dichloroacetic acid, 6-ethyl-3-octyl ester
**0.13**	Dodecane, 4,6-dimethyl-
**0.13**	Octacosane
**0.13**	Pentadecane
**0.13**	Undecane, 2,3-dimethyl-
**0.14**	Hexadecane, 2,6,11,15-tetramethyl-
**0.14**	Tetradecanal
**0.15**	Hexadecane
**0.15**	Silane, cyclohexyldimethoxymethyl-
**0.16**	Decane, 4-methyl-
**0.16**	Hexadecane
**0.16**	Hexadecane, 1-iodo-
**0.16**	Sulfurous acid, hexyl pentadecyl ester
**0.17**	Tetratriacontane
**0.18**	Cycloheptasiloxane, tetradecamethyl-
**0.18**	Tetradecane, 4-methyl-
**0.21**	1-Heptanol, 2,4-diethyl-
**0.21**	Pentadecane, 2,6,10,14-tetramethyl-
**0.23**	Decane, 5-propyl-
**0.23**	Eicosane
**0.23**	Eicosane
**0.26**	Octane, 2,6,6-trimethyl-
**0.27**	3-Tridecene, (Z)-
**0.27**	Decane, 2,8,8-trimethyl-

**0.3**	3-Octanol, 3,7-dimethyl-
**0.3**	Eicosane
**0.3**	Undecane
**0.31**	Acetamide, N,N-dimethyl-
**0.31**	Tetradecane, 2-methyl-
**0.32**	Heptadecane
**0.33**	1-Butanol, 3-methoxy-, acetate
**0.35**	1-Octene, 3,7-dimethyl-
**0.35**	Nonane, 5-(2-methylpropyl)-
**0.4**	Decane, 2,8,8-trimethyl-
**0.41**	Undecane, 3-methyl-
**0.44**	Hexadecane
**0.44**	Hexadecane
**0.5**	Isotridecanol-
**0.51**	Phenol, 2,4-bis(1,1-dimethylethyl)-
**0.53**	1-Tetradecanol
**0.54**	Dodecane, 4,6-dimethyl-
**0.55**	Cyclopentasiloxane, decamethyl-
**0.57**	Dodecane, 4,6-dimethyl-
**0.57**	Isotridecanol-
**0.61**	Dodecane, 4,6-dimethyl-
**0.61**	Eicosane
**0.62**	Dodecane, 4,6-dimethyl-
**0.86**	Dodecane, 4,6-dimethyl-
**0.87**	Acetic acid, butyl ester
**0.91**	Tetradecane
**1**	1-Decanol, 2-hexyl-
**1.07**	Dodecane, 4,6-dimethyl-
**1.08**	Dodecane, 4,6-dimethyl-
**1.08**	Heptasiloxane, hexadecamethyl-
**1.1**	Dodecane, 4,6-dimethyl-
**1.12**	Dodecane, 4,6-dimethyl-
**1.34**	Undecane, 4-methyl-
**1.4**	Heptane, 2,5,5-trimethyl-
**1.4**	Nonane, 2,6-dimethyl-
**1.45**	Dodecane, 4,6-dimethyl-
**1.47**	Decane
**1.47**	Dodecane, 4,6-dimethyl-
**1.53**	Dodecane, 4,6-dimethyl-
**1.54**	Eicosane
**1.54**	Hexasiloxane, tetradecamethyl-
**1.7**	Dodecane, 4,6-dimethyl-
**1.75**	Eicosane
**2.05**	1-Undecene, 7-methyl-
**2.05**	Octane, 6-ethyl-2-methyl-

EXCLI Journal 2021;20:995-1008 – ISSN 1611-2156
Received: April 08, 2021, accepted: May 26, 2021, published: June 01, 2021

**2.15**	Eicosane
**2.4**	1-Undecene, 7-methyl-
**2.49**	Dodecane
**2.52**	Octane, 6-ethyl-2-methyl-
**3.16**	Isotridecanol-
**3.18**	1-Undecene, 7-methyl-
**3.25**	Caprolactam
**4.03**	1-Methoxy-2-propyl acetate
**4.36**	1-Undecene, 7-methyl-
**4.43**	Dodecane, 4,6-dimethyl-
**5.19**	Octane, 6-ethyl-2-methyl-
**5.79**	Octane, 5-ethyl-2-methyl-
**8.18**	Hexadecane

EXCLI Journal 2021;20:995-1008 – ISSN 1611-2156
Received: April 08, 2021, accepted: May 26, 2021, published: June 01, 2021

Sample AA
Number of peaks: 94
Number of identified compounds: 65
Ratio of identified peak area: 90.98%

Peak Area [%]	Database comparison
0.1	Octane, 6-ethyl-2-methyl-
0.11	Triacontane, 1-bromo-
0.14	Cyclotetrasiloxane, octamethyl-
0.14	Decane, 3,3,8-trimethyl-
0.14	Octanal
0.15	Pentadecane, 2,6,10,14-tetramethyl-
0.16	Octadecane, 1-iodo-
0.19	1-Pentadecene
0.21	Hexadecane
0.21	Octane, 2,3,6,7-tetramethyl-
0.24	Tetradecane, 4-methyl-
0.26	Hexadecane, 2,6,11,15-tetramethyl-
0.27	2,5-Cyclohexadiene-1,4-dione, 2,6-bis(1,1-dimethylethyl)-
0.27	Octane, 1,1'-oxybis-
0.28	Octane, 6-ethyl-2-methyl-
0.29	Decane, 2,2-dimethyl-
0.29	Nonane, 5-butyl-
0.31	5,5-Dibutylnonane
0.36	Hexasiloxane, tetradecamethyl-
0.36	Nonane, 5-butyl-
0.38	1-Methoxy-2-propyl acetate
0.39	Dodecane, 2,6,10-trimethyl-
0.4	Cyclopentane, undecyl-
0.4	Dodecane, 4,6-dimethyl-
0.4	Octane, 2,3,6,7-tetramethyl-
0.41	1-Decanol, 2-hexyl-
0.41	Dodecane, 4,6-dimethyl-
0.42	Decane, 2,8,8-trimethyl-
0.49	Hexadecane, 1,1-bis(dodecyloxy)-
0.52	Dodecane, 4,6-dimethyl-
0.63	Dodecane, 4,6-dimethyl-
0.63	Octadecane
0.68	Eicosane
0.72	Eicosane
0.82	Decanal
0.86	1-Tridecene
0.87	Eicosane
0.88	Cyclopentasiloxane, decamethyl-
0.93	Dodecane, 4,6-dimethyl-
0.96	Caprolactam

EXCLI Journal 2021;20:995-1008 – ISSN 1611-2156
Received: April 08, 2021, accepted: May 26, 2021, published: June 01, 2021

**1.1**	Cyclohexane, 1-ethyl-2-propyl-
**1.15**	Undecane, 2,5-dimethyl-
**1.23**	Nonane, 5-(2-methylpropyl)-
**1.31**	1-Hexadecanol, 3,7,11,15-tetramethyl-
**1.44**	Dodecane, 4,6-dimethyl-
**1.51**	Undecane
**1.52**	Dodecane, 4,6-dimethyl-
**1.54**	Eicosane
**1.66**	Tetradecane
**1.67**	Pentadecane
**1.83**	1-Butanol, 3-methoxy-
**2.02**	Sulfurous acid, pentyl tridecyl ester
**2.12**	Heptasiloxane, hexadecamethyl-
**2.21**	Isotridecanol-
**2.48**	Dodecane, 4,6-dimethyl-
**2.73**	Eicosane
**2.82**	Dodecane, 4,6-dimethyl-
**2.94**	1-Undecene, 7-methyl-
**3.06**	2-Isopropyl-5-methyl-1-heptanol
**4.07**	Eicosane
**4.37**	Dodecane
**5.68**	Heptadecane
**5.8**	Eicosane
**6.94**	Dodecane, 4,6-dimethyl-
**11.1**	Dodecane, 2,6,11-trimethyl-

EXCLI Journal 2021;20:995-1008 – ISSN 1611-2156
Received: April 08, 2021, accepted: May 26, 2021, published: June 01, 2021

Sample AB
Number of peaks: 108
Number of identified compounds: 66
Ratio of identified peak area: 91.16%

Peak Area [%]	Database comparison
0.1	Cyclotetrasiloxane, octamethyl-
0.1	Dodecane, 4-methyl-
0.11	Cyclododecane
0.12	Octanal
0.13	Decane, 2,3,8-trimethyl-
0.14	Tetradecane, 4-methyl-
0.2	Octane, 5-ethyl-2-methyl-
0.28	Undecane, 4-methyl-
0.29	Eicosane
0.32	1-Hexanol, 2-ethyl-
0.33	Octane, 6-ethyl-2-methyl-
0.35	trans-2-Dodecen-1-ol
0.44	1-Methoxy-2-propyl acetate
0.44	2-Propenoic acid, 2-ethylhexyl ester
0.44	Eicosane
0.44	Sulfurous acid, pentadecyl pentyl ester
0.45	Dodecane, 4,6-dimethyl-
0.45	Hexadecane
0.46	Hexasiloxane, tetradecamethyl-
0.48	Cyclotetracosane
0.48	Dodecane, 4-methyl-
0.49	Cyclopentasiloxane, decamethyl-
0.51	Octane, 2,3,6,7-tetramethyl-
0.61	Tetradecane, 5-methyl-
0.62	Tetradecane, 4-methyl-
0.67	Decanal
0.69	Isotridecanol-
0.69	Tridecyl heptafluorobutyrate
0.7	Dodecane, 4,6-dimethyl-
0.74	Decane, 5-propyl-
0.75	Dodecane, 4,6-dimethyl-
0.8	Hexadecane
0.82	Sulfurous acid, pentyl undecyl ester
0.86	Decane, 5-propyl-
0.86	Undecane, 2,5-dimethyl-
0.89	Dodecane, 4,6-dimethyl-
0.9	Phenol, 2,4-bis(1,1-dimethylethyl)-
0.93	Dodecane, 4,6-dimethyl-
1.01	Pentadecane, 2,6,10,14-tetramethyl-
1.05	Heptadecane, 8-methyl-

EXCLI Journal 2021;20:995-1008 – ISSN 1611-2156
Received: April 08, 2021, accepted: May 26, 2021, published: June 01, 2021

**1.06**	Tetradecane, 2-methyl-
**1.1**	Undecane
**1.2**	Eicosane
**1.21**	1-Butanol, 3-methoxy-
**1.28**	Dodecane, 4,6-dimethyl-
**1.4**	Sulfurous acid, pentyl tridecyl ester
**1.52**	Dodecane, 4-methyl-
**1.58**	Hexadecane
**1.62**	Dodecane, 4,6-dimethyl-
**1.68**	1-Dodecanol
**1.86**	Eicosane
**1.88**	Dodecane, 4,6-dimethyl-
**1.89**	Heptasiloxane, hexadecamethyl-
**1.98**	Dodecane, 4,6-dimethyl-
**1.98**	Octacosane
**2.15**	Tetradecane
**2.17**	Hexadecane
**2.75**	Isotridecanol-
**3.07**	Dodecane
**3.11**	1-Undecene, 7-methyl-
**3.31**	Caprolactam
**3.33**	Isotridecanol-
**3.59**	Hexadecane, 2,6,11,15-tetramethyl-
**5.46**	Dodecane, 4,6-dimethyl-
**6.71**	Eicosane
**11.13**	Hexadecane

EXCLI Journal 2021;20:995-1008 – ISSN 1611-2156
Received: April 08, 2021, accepted: May 26, 2021, published: June 01, 2021

Sample AC
Number of peaks: 106
Number of identified compounds: 76
Ratio of identified peak area: 95.57%

Peak Area [%]	Database comparison
**0.09**	Decane, 3,3,4-trimethyl-
**0.09**	Oxygen
**0.1**	Decane, 2,3,8-trimethyl-
**0.11**	Oxalic acid, bis(6-ethyloct-3-yl) ester
**0.12**	Cyclododecane
**0.14**	Cyclopentasiloxane, decamethyl-
**0.16**	2,5-Cyclohexadiene-1,4-dione, 2,6-bis(1,1-dimethylethyl)-
**0.17**	Nonacosane
**0.18**	Dichloroacetic acid, 6-ethyl-3-octyl ester
**0.18**	Heneicosane
**0.19**	Nonane, 3-methyl-
**0.2**	Decane, 3-ethyl-3-methyl-
**0.21**	1-Hexadecanesulfonyl chloride
**0.21**	1-Octanol, 2,7-dimethyl-
**0.21**	Pentadecane, 2,6,10,14-tetramethyl-
**0.21**	p-Xylene
**0.25**	Dodecanal
**0.25**	Eicosane
**0.26**	Decanal
**0.26**	Octane, 5-ethyl-2-methyl-
**0.3**	Tetratriacontane
**0.31**	Heneicosane
**0.36**	Tetradecane, 4-methyl-
**0.37**	Benzene, 3-cyclohexen-1-yl-
**0.38**	1-Undecene, 9-methyl-
**0.38**	Heptafluorobutanoic acid, heptadecyl ester
**0.38**	Hexadecane
**0.4**	3-Eicosene, (E)-
**0.41**	Caprolactam
**0.43**	Octane, 2,6,6-trimethyl-
**0.51**	2-Bromo dodecane
**0.52**	Decane, 5-propyl-
**0.55**	Hexasiloxane, tetradecamethyl-
**0.56**	Heptadecyl heptafluorobutyrate
**0.58**	Hexadecane, 2,6,10,14-tetramethyl-
**0.59**	Nonane, 2,6-dimethyl-
**0.59**	Pentadecane, 7-methyl-
**0.62**	Dodecane, 4,6-dimethyl-
**0.62**	Octane, 5-ethyl-2-methyl-
**0.65**	1-Decene, 2,4-dimethyl-

EXCLI Journal 2021;20:995-1008 – ISSN 1611-2156
Received: April 08, 2021, accepted: May 26, 2021, published: June 01, 2021

**0.65**	1-Methoxy-2-propyl acetate
**0.65**	Undecane
**0.72**	1-Undecene, 7-methyl-
**0.77**	Dodecane, 4,6-dimethyl-
**0.83**	Dodecane, 4,6-dimethyl-
**0.88**	Isotridecanol-
**0.9**	Dodecane, 4-methyl-
**0.91**	Phenol, 2,5-bis(1,1-dimethylethyl)-
**0.93**	1-Butanol, 3-methoxy-
**0.98**	Octane, 6-ethyl-2-methyl-
**0.98**	Tetradecane
**0.99**	Nonane, 5-methyl-5-propyl-
**1.1**	Heptasiloxane, hexadecamethyl-
**1.19**	Dodecane, 4,6-dimethyl-
**1.37**	Dodecane, 4,6-dimethyl-
**1.38**	Dodecane, 4,6-dimethyl-
**1.38**	Dodecane, 4,6-dimethyl-
**1.39**	Dodecane, 4,6-dimethyl-
**1.44**	1-Heptanol, 2,4-diethyl-
**1.59**	Dodecane, 4,6-dimethyl-
**1.65**	Sulfurous acid, pentyl tridecyl ester
**1.84**	Dodecane, 4,6-dimethyl-
**2.03**	Dodecane, 4,6-dimethyl-
**2.08**	Propanoic acid, 2-methyl-, 1-(1,1-dimethylethyl)-2-methyl-1,3-propanediyl ester
**2.24**	Eicosane
**2.26**	1-Hexadecanesulfonyl chloride
**2.33**	Dodecane, 4,6-dimethyl-
**2.48**	Dodecane
**2.81**	Octacosane
**3.04**	Eicosane
**3.28**	Heptasiloxane, hexadecamethyl-
**4.17**	Dodecane, 4,6-dimethyl-
**6.12**	Hexadecane
**7.51**	2-Isopropyl-5-methyl-1-heptanol
**7.63**	Isotridecanol-
**9.97**	Isotridecanol-

Received: April 08, 2021, accepted: May 26, 2021, published: June 01, 2021

Sample AD
Number of peaks: 97
Number of identified compounds: 70
Ratio of identified peak area: 94.93%

Peak Area [%]	Database comparison
**0.13**	1-Butanol, 3-methoxy-, acetate
**0.14**	Sulfurous acid, dodecyl hexyl ester
**0.15**	Dodecane, 2-methyl-
**0.19**	Cyclotetrasiloxane, octamethyl-
**0.19**	Oxygen
**0.21**	Nonane, 5-methyl-5-propyl-
**0.22**	Dodecane, 4-methyl-
**0.22**	Eicosane
**0.24**	Tetradecane, 4-methyl-
**0.27**	Benzeneethanol, .alpha.,.beta.-dimethyl-
**0.3**	Heptane, 2,2,4-trimethyl-
**0.31**	1-Octanol, 2-methyl-
**0.33**	7-Tetradecene
**0.33**	Eicosane
**0.33**	Octane, 5-ethyl-2-methyl-
**0.34**	Cyclopentane, nonyl-
**0.35**	Eicosane
**0.35**	Isopropyl Palmitate
**0.41**	1-Undecene, 7-methyl-
**0.41**	Sulfurous acid, pentyl undecyl ester
**0.43**	Eicosane
**0.43**	Heptasiloxane, hexadecamethyl-
**0.44**	Octane, 6-ethyl-2-methyl-
**0.49**	Hexadecane
**0.51**	1-Undecene, 7-methyl-
**0.55**	Decane, 2,8,8-trimethyl-
**0.62**	Hexasiloxane, tetradecamethyl-
**0.63**	2-Propenoic acid, 2-ethylhexyl ester
**0.63**	Disulfide, di-tert-dodecyl
**0.65**	Decanal
**0.67**	Decane, 3,3,8-trimethyl-
**0.72**	Dodecane, 4,6-dimethyl-
**0.73**	Heneicosane
**0.78**	Hexadecane
**0.85**	Dodecane, 4-methyl-
**0.85**	Octane, 5-ethyl-2-methyl-
**0.87**	3,5-Dimethyldodecane
**0.93**	Octane, 2,3,6,7-tetramethyl-
**0.94**	Dodecane, 4,6-dimethyl-
**0.97**	Decane, 3,3,6-trimethyl-

EXCLI Journal 2021;20:995-1008 – ISSN 1611-2156
Received: April 08, 2021, accepted: May 26, 2021, published: June 01, 2021

**1.05**	Cyclopentasiloxane, decamethyl-
**1.11**	Caprolactam
**1.13**	Dodecane, 4,6-dimethyl-
**1.13**	Dodecane, 4,6-dimethyl-
**1.15**	Cycloheptasiloxane, tetradecamethyl-
**1.23**	Heptasiloxane, hexadecamethyl-
**1.23**	Nonane, 5-methyl-5-propyl-
**1.24**	Nonane, 5-methyl-5-propyl-
**1.25**	1-Hexadecanesulfonyl chloride
**1.27**	Dodecane, 4,6-dimethyl-
**1.35**	Isotridecanol-
**1.37**	Benzene, 1,1'-(1,1,2,2-tetramethyl-1,2-ethanediyl)bis-
**1.45**	Sulfurous acid, pentadecyl pentyl ester
**1.5**	Dodecane, 4,6-dimethyl-
**1.52**	Dodecane, 4,6-dimethyl-
**1.53**	Undecane
**1.8**	Tetradecane
**1.99**	1-Methoxy-2-propyl acetate
**1.99**	Hexadecane
**2.27**	1-Decanol, 2-hexyl-
**3.06**	Eicosane
**3.12**	1-Butanol, 3-methoxy-
**3.57**	Isotridecanol-
**3.59**	Heptadecane
**3.84**	Hexadecane
**3.9**	Dodecane
**3.96**	1-Undecene, 7-methyl-
**5.2**	Dodecane, 4,6-dimethyl-
**8.33**	1-Heptanol, 2,4-diethyl-
**8.69**	Hexadecane

EXCLI Journal 2021;20:995-1008 – ISSN 1611-2156
Received: April 08, 2021, accepted: May 26, 2021, published: June 01, 2021

Sample AE
Number of peaks: 113
Number of identified compounds: 81
Ratio of identified peak area: 97.01%

Peak Area [%]	Database comparison
0.04	Dodecane, 2-methyl-
0.05	Undecane, 3-methyl-
0.06	Nonane, 2,5-dimethyl-
0.06	Octane, 3-methyl-
0.08	Benzaldehyde
0.08	Heptane, 2,5,5-trimethyl-
0.08	Hexane, 2,2,5-trimethyl-
0.08	Undecane, 2-methyl-
0.09	Oxalic acid, 6-ethyloct-3-yl isohexyl ester
0.09	Undecane
0.1	Heptane, 2,5,5-trimethyl-
0.11	Sulfurous acid, pentyl undecyl ester
0.12	Nonane, 5-(2-methylpropyl)-
0.12	Octane, 2,3,6,7-tetramethyl-
0.12	Tetradecane, 4-methyl-
0.14	Hexasiloxane, tetradecamethyl-
0.15	Dodecane, 4,6-dimethyl-
0.15	Dodecane, 4,6-dimethyl-
0.15	Eicosane
0.15	Tetradecane, 4-methyl-
0.16	Dodecane, 2,7,10-trimethyl-
0.16	Hexadecane, 2,6,10,14-tetramethyl-
0.16	Nonacosane
0.16	Tetradecane, 2-methyl-
0.16	Tetradecane, 5-methyl-
0.17	Octadecanal
0.18	p-Xylene
0.19	7-Tetradecene
0.2	1-Dodecene
0.2	Heptasiloxane, hexadecamethyl-
0.2	Undecane, 3,8-dimethyl-
0.21	Heptadecane
0.21	Hexadecane
0.22	Pentadecane, 7-methyl-
0.23	Hexadecane
0.24	Sulfurous acid, nonyl 2-pentyl ester
0.25	Eicosane
0.26	Octadecane
0.29	Decanal
0.3	Cyclotetrasiloxane, octamethyl-

EXCLI Journal 2021;20:995-1008 – ISSN 1611-2156
Received: April 08, 2021, accepted: May 26, 2021, published: June 01, 2021

**0.32**	Hexadecane
**0.34**	Dodecane, 4,6-dimethyl-
**0.39**	Dodecane, 4,6-dimethyl-
**0.45**	Nonane, 5-methyl-5-propyl-
**0.47**	Dodecane, 4-methyl-
**0.49**	Tetradecane
**0.5**	1-Heptanol, 6-methyl-
**0.58**	Sulfurous acid, dodecyl pentyl ester
**0.6**	Cyclopentasiloxane, decamethyl-
**0.62**	Eicosane
**0.64**	Hexadecane
**0.66**	Heptadecane
**0.73**	Caprolactam
**0.74**	Undecane
**0.77**	Acetamide, N,N-dimethyl-
**0.78**	Dodecane, 4,6-dimethyl-
**0.81**	1-Methoxy-2-propyl acetate
**0.86**	Heptane, 2,5,5-trimethyl-
**0.97**	Dodecane, 4,6-dimethyl-
**1.05**	1-Butanol, 3-methoxy-
**1.1**	Dodecane, 4,6-dimethyl-
**1.25**	Nonane, 2,6-dimethyl-
**1.28**	Isotridecanol-
**1.41**	Isotridecanol-
**1.66**	Dodecane
**1.67**	Dodecane, 4,6-dimethyl-
**1.69**	1-Decene, 2,4-dimethyl-
**1.71**	Hexadecane, 2,6,11,15-tetramethyl-
**1.73**	Dodecane, 4,6-dimethyl-
**1.73**	Octane, 2,3,6,7-tetramethyl-
**1.79**	Isotridecanol-
**2.13**	1-Undecene, 7-methyl-
**2.27**	Dodecane, 4,6-dimethyl-
**2.65**	Dodecane, 4,6-dimethyl-
**2.93**	Heneicosane
**4.05**	Octane, 6-ethyl-2-methyl-
**4.81**	Octane, 6-ethyl-2-methyl-
**5.69**	Dodecane, 4,6-dimethyl-
**8.81**	Octane, 6-ethyl-2-methyl-
**13.24**	Hexadecane
**14.47**	Octane, 5-ethyl-2-methyl-

EXCLI Journal 2021;20:995-1008 – ISSN 1611-2156
Received: April 08, 2021, accepted: May 26, 2021, published: June 01, 2021

Sample AF
Number of peaks: 120
Number of identified compounds: 89
Ratio of identified peak area: 98.18%

Peak Area [%]	Database comparison
**0.03**	Heptadecane
**0.04**	Cyclopentane, decyl-
**0.04**	Dodecanal
**0.04**	Heneicosane
**0.04**	Nonane, 3-methyl-5-propyl-
**0.05**	1-Undecene, 7-methyl-
**0.05**	3-Tetradecene, (E)-
**0.05**	Dodecane, 4,6-dimethyl-
**0.05**	Eicosane
**0.05**	Undecane, 3,8-dimethyl-
**0.06**	Cyclopentasiloxane, decamethyl-
**0.06**	Dodecane, 4-methyl-
**0.06**	Eicosane
**0.06**	Tetradecane
**0.07**	Benzene, 1,1'-(1,1,2,2-tetramethyl-1,2-ethanediyl)bis-
**0.07**	Propanoic acid, 2-methyl-, octyl ester
**0.08**	2,2,4,4-Tetramethyloctane
**0.09**	Decane, 5,6-dimethyl-
**0.09**	Decyl trifluoroacetate
**0.09**	Dodecane, 4,6-dimethyl-
**0.09**	Dodecane, 4,6-dimethyl-
**0.09**	p-Xylene
**0.1**	1-Undecene, 4-methyl-
**0.1**	Decane, 4-methyl-
**0.1**	Eicosane
**0.1**	Hexadecane
**0.14**	Decanal
**0.15**	Eicosane
**0.16**	Tetradecane
**0.17**	Dodecane, 4,6-dimethyl-
**0.18**	1-Hexadecanesulfonyl chloride
**0.18**	Eicosane
**0.18**	Tetradecane
**0.21**	Heneicosane
**0.21**	Undecane, 3,7-dimethyl-
**0.22**	Decane, 2,8,8-trimethyl-
**0.22**	Dodecane, 4,6-dimethyl-
**0.23**	Dodecane, 4,6-dimethyl-
**0.23**	Dodecane, 4,6-dimethyl-
**0.24**	Undecane

EXCLI Journal 2021;20:995-1008 – ISSN 1611-2156
Received: April 08, 2021, accepted: May 26, 2021, published: June 01, 2021

**0.25**	Dodecane, 4,6-dimethyl-
**0.25**	Hexane, 2,3,4-trimethyl-
**0.25**	Isotridecanol-
**0.26**	Eicosane
**0.27**	Decane
**0.31**	Caprolactam
**0.31**	Decane, 2,9-dimethyl-
**0.31**	Tetradecane
**0.33**	Decane, 3,3,5-trimethyl-
**0.33**	Undecane
**0.37**	1,3-Dioxolane-2-acetic acid, 2,4-dimethyl-, ethyl ester
**0.38**	Undecane
**0.47**	Hexadecane, 2,6,11,15-tetramethyl-
**0.48**	Eicosane
**0.5**	Dodecane, 4,6-dimethyl-
**0.53**	Dodecane, 4,6-dimethyl-
**0.57**	Hexasiloxane, tetradecamethyl-
**0.58**	Heptadecane
**0.59**	1,3-Dioxolane-2-acetic acid, 2,4-dimethyl-, ethyl ester
**0.62**	Undecane
**0.65**	Eicosane
**0.76**	Dodecane, 4,6-dimethyl-
**0.83**	Dodecane, 4,6-dimethyl-
**1.01**	Acetic acid, 3-methoxy-2-butyl ester
**1.17**	Heptane, 2,5,5-trimethyl-
**1.17**	Octane, 6-ethyl-2-methyl-
**1.18**	Acetic acid, butyl ester
**1.18**	Dodecane
**1.19**	Dodecane, 4-methyl-
**1.2**	Eicosane
**1.37**	Dodecane, 4,6-dimethyl-
**1.42**	Isotridecanol-
**1.5**	Isotridecanol-
**1.55**	Dodecane, 4,6-dimethyl-
**1.64**	1-Methoxy-2-propyl acetate
**1.78**	Dodecane, 4,6-dimethyl-
**1.78**	Isotridecanol-
**1.8**	1-Undecene, 7-methyl-
**1.96**	Dodecane, 4,6-dimethyl-
**2.01**	Dodecane, 4,6-dimethyl-
**2.29**	Eicosane
**2.53**	Octane, 2,3,6,7-tetramethyl-
**2.82**	1-Undecene, 7-methyl-
**4.05**	Dodecane, 4,6-dimethyl-
**5.05**	Octane, 6-ethyl-2-methyl-

EXCLI Journal 2021;20:995-1008 – ISSN 1611-2156
Received: April 08, 2021, accepted: May 26, 2021, published: June 01, 2021

**6.37**	Octane, 6-ethyl-2-methyl-
**9.03**	Octane, 6-ethyl-2-methyl-
**9.25**	Dodecane, 4,6-dimethyl-
**17.21**	Octane, 6-ethyl-2-methyl-

EXCLI Journal 2021;20:995-1008 – ISSN 1611-2156
Received: April 08, 2021, accepted: May 26, 2021, published: June 01, 2021

Sample AG
Number of peaks: 137
Number of identified compounds: 88
Ratio of identified peak area: 87.32%

Peak Area [%]	Database comparison
0.04	Undecane. 2-methyl-
0.05	1-Butanol. 3-methoxy-. acetate
0.05	4-Decene. 7-methyl-. (E)-
0.05	Oxygen
0.06	Heptadecane
0.08	1H-Indene. 2.3-dihydro-4-methyl-
0.1	Cyclopentasiloxane. decamethyl-
0.11	Cyclopropane. nonyl-
0.11	Heneicosane
0.12	2.5-Cyclohexadiene-1.4-dione. 2.6-bis(1.1-dimethylethyl)-
0.12	Pentatriacontane
0.14	2-Undecene. 2.5-dimethyl-
0.14	Dodecane. 4.6-dimethyl-
0.14	Pentadecane
0.14	Pentadecane. 2.6.10.14-tetramethyl-
0.18	Benzene. 1.2.3.5-tetramethyl-
0.19	Benzene. 1-ethyl-2.4.5-trimethyl-
0.22	Dodecane. 3-methyl-
0.22	Pentadecane. 2.6.10.14-tetramethyl-
0.22	p-Xylene
0.22	Undecane. 5-methyl-
0.24	Cyclopropane. 1.1-dimethyl-2-(3-methyl-1.3-butadienyl)-
0.24	Heptane. 2.5.5-trimethyl-
0.29	1H-Indene. 2.3-dihydro-4-methyl-
0.31	Undecane. 3-methyl-
0.32	Octane. 5-ethyl-2-methyl-
0.33	Disulfide. di-tert-dodecyl
0.33	Tridecane. 4-methyl-
0.37	Tetradecane. 2-methyl-
0.38	5-Tridecene. (E)-
0.38	Oxirane. hexadecyl-
0.4	Octane. 2.3.6-trimethyl-
0.41	Dodecane. 4.6-dimethyl-
0.42	Benzene. 1-ethyl-2.4.5-trimethyl-
0.49	1-Methoxy-2-propyl acetate
0.54	Undecane
0.55	Phenol. 3.5-bis(1.1-dimethylethyl)-
0.62	Octane. 6-ethyl-2-methyl-
0.64	2-Isopropyl-5-methyl-1-heptanol
0.64	Tetradecane. 3-methyl-

EXCLI Journal 2021;20:995-1008 – ISSN 1611-2156
Received: April 08, 2021, accepted: May 26, 2021, published: June 01, 2021

**0.65**	Heptasiloxane. hexadecamethyl-
**0.65**	Nonane. 5-methyl-5-propyl-
**0.68**	Benzene. 1.2.3.4-tetramethyl-
**0.68**	Oxalic acid. 6-ethyloct-3-yl heptyl ester
**0.69**	1-Hexadecanesulfonyl chloride
**0.72**	2-Bromo dodecane
**0.74**	Hexadecane
**0.74**	Octane. 2.3.6.7-tetramethyl-
**0.78**	1-Butanol. 3-methoxy-
**0.82**	Tetradecane. 5-methyl-
**0.87**	Dodecane. 4.6-dimethyl-
**0.89**	Nonane. 5-methyl-5-propyl-
**0.91**	Tetradecanal
**0.92**	Hexadecane. 2.6.10.14-tetramethyl-
**0.95**	Dodecane. 4-methyl-
**0.97**	1-Octanol. 2.7-dimethyl-
**0.98**	1-Undecene. 7-methyl-
**1**	Hexadecane
**1.07**	Tetradecane. 4-methyl-
**1.08**	Dodecane. 4.6-dimethyl-
**1.09**	Isotridecanol-
**1.14**	Octane. 5-ethyl-2-methyl-
**1.16**	Decane. 2.8.8-trimethyl-
**1.2**	Tetradecane
**1.24**	Caprolactam
**1.26**	Dodecane. 4.6-dimethyl-
**1.28**	Heptasiloxane. hexadecamethyl-
**1.29**	Sulfurous acid. decyl 2-pentyl ester
**1.35**	Octane. 5-ethyl-2-methyl-
**1.37**	Decane. 2.8.8-trimethyl-
**1.4**	Hexadecane
**1.51**	Hexasiloxane. tetradecamethyl-
**1.57**	Hexadecane. 2.6.10.14-tetramethyl-
**1.57**	Tetrapentacontane. 1.54-dibromo-
**1.7**	Dodecane. 4.6-dimethyl-
**1.81**	1-Hexadecanesulfonyl chloride
**1.83**	Dodecane. 4.6-dimethyl-
**1.95**	Eicosane
**1.98**	Eicosane
**2.2**	Hexadecane
**2.57**	Heptadecane
**2.86**	Nonane. 5-methyl-5-propyl-
**2.96**	2-Cyclohexen-1-one. 3.5.5-trimethyl-
**3.31**	Dodecane
**3.76**	1-Heptanol. 2.4-diethyl-

**3.99**	1-Heptanol. 2.4-diethyl-
**5.21**	Hexadecane
**5.37**	2-Isopropyl-5-methyl-1-heptanol

EXCLI Journal 2021;20:995-1008 – ISSN 1611-2156
Received: April 08, 2021, accepted: May 26, 2021, published: June 01, 2021

Sample AH
Number of peaks: 53
Number of identified compounds: 32
Ratio of identified peak area: 82.97%

Peak Area [%]	Database comparison
**0.55**	Undecane
**0.59**	Undecane, 3,6-dimethyl-
**0.62**	Dodecane, 4,6-dimethyl-
**0.72**	Dodecane, 4,6-dimethyl-
**0.95**	Hexasiloxane, tetradecamethyl-
**1.01**	Caprolactam
**1.08**	3-Hexadecene, (Z)-
**1.13**	Hexadecane, 2,6,10,14-tetramethyl-
**1.14**	Dodecane, 4,6-dimethyl-
**1.31**	Acetophenone
**1.4**	1-Hexadecanol, 3,7,11,15-tetramethyl-
**1.45**	Dodecane
**1.45**	Eicosane
**1.63**	Eicosane
**1.69**	Dodecane, 4,6-dimethyl-
**1.73**	1-Undecene, 7-methyl-
**1.78**	Decanal
**1.89**	Dodecane, 4,6-dimethyl-
**1.98**	Hexadecane
**2.09**	Cyclopentasiloxane, decamethyl-
**2.29**	Isotridecanol-
**2.35**	Nonane, 5-methyl-5-propyl-
**2.58**	Isotridecanol-
**2.72**	Dodecane, 4,6-dimethyl-
**2.97**	Isotridecanol-
**3.92**	Heptasiloxane, hexadecamethyl-
**3.98**	Tetradecane
**4.77**	Octacosane
**5.04**	Eicosane
**5.55**	Eicosane
**5.67**	Hexadecane
**14.94**	Nonanal

EXCLI Journal 2021;20:995-1008 – ISSN 1611-2156
Received: April 08, 2021, accepted: May 26, 2021, published: June 01, 2021

Sample AI
Number of peaks: 101
Number of identified compounds: 69
Ratio of identified peak area: 94.19%

Peak Area [%]	Database comparison
**0.11**	Tridecane, 4-methyl-
**0.12**	Nonane, 3-methyl-5-propyl-
**0.14**	Cyclododecane
**0.16**	3,3,5-Trimethylcyclohexyl acrylate
**0.16**	Eicosane
**0.16**	Pentadecane, 8-hexyl-
**0.2**	Hexadecane, 2,6,10,14-tetramethyl-
**0.2**	Tetradecyl trifluoroacetate
**0.22**	Undecane, 2,5-dimethyl-
**0.25**	Decane, 4-methyl-
**0.25**	Octadecane, 1-(ethenyloxy)-
**0.27**	Decane, 3,3,8-trimethyl-
**0.28**	Hexadecane, 2,6,10,14-tetramethyl-
**0.31**	Decane, 5-propyl-
**0.32**	Undecane, 3-methyl-
**0.33**	Dodecane, 2,6,10-trimethyl-
**0.35**	Decane
**0.36**	Cyclopentasiloxane, decamethyl-
**0.39**	Nonane, 5-(2-methylpropyl)-
**0.43**	2-Propenoic acid, 2-ethylhexyl ester
**0.46**	Dodecane, 4,6-dimethyl-
**0.47**	Pentadecane, 2,6,10,14-tetramethyl-
**0.5**	Hexadecane, 2,6,10,14-tetramethyl-
**0.51**	D-Limonene
**0.54**	Nonane, 2,3-dimethyl-
**0.55**	Disulfide, di-tert-dodecyl
**0.63**	Cyclopentane, 1-pentyl-2-propyl-
**0.64**	Eicosane
**0.67**	Tetradecanal
**0.69**	2-Bromo dodecane
**0.7**	Hexadecane
**0.74**	Dodecane, 4,6-dimethyl-
**0.74**	Hexasiloxane, tetradecamethyl-
**0.79**	Dodecane, 4,6-dimethyl-
**0.8**	Isotridecanol-
**0.82**	Dodecane, 4,6-dimethyl-
**0.83**	Octane, 6-ethyl-2-methyl-
**0.86**	Octane, 6-ethyl-2-methyl-
**0.87**	Heptasiloxane, hexadecamethyl-
**0.87**	Undecane

EXCLI Journal 2021;20:995-1008 – ISSN 1611-2156
Received: April 08, 2021, accepted: May 26, 2021, published: June 01, 2021

**0.98**	Dodecane, 4,6-dimethyl-
**1.01**	1-Undecene, 7-methyl-
**1.1**	1-Undecene, 7-methyl-
**1.1**	Decane, 2,8,8-trimethyl-
**1.29**	Dodecane, 4,6-dimethyl-
**1.44**	Dodecane, 4,6-dimethyl-
**1.48**	1-Decanol, 2-hexyl-
**1.54**	Hexadecane
**1.56**	Cyclopentanone, 2-methyl-
**1.61**	Octane, 2,3,6,7-tetramethyl-
**1.65**	Dodecane, 4,6-dimethyl-
**1.68**	Nonane, 5-(2-methylpropyl)-
**1.69**	Caprolactam
**1.8**	Dodecane, 4,6-dimethyl-
**1.96**	Eicosane
**2**	Heptasiloxane, hexadecamethyl-
**2.1**	Dodecane, 4,6-dimethyl-
**2.11**	Dodecane, 4,6-dimethyl-
**2.18**	Dodecane, 4-methyl-
**2.51**	Heneicosane
**2.59**	Eicosane
**2.65**	Tetradecane
**3.02**	Octane, 5-ethyl-2-methyl-
**3.59**	Dodecane
**4.02**	Isotridecanol-
**4.38**	1-Heptanol, 2,4-diethyl-
**5.89**	1-Heptanol, 2,4-diethyl-
**6.77**	Dodecane, 4,6-dimethyl-
**9.8**	Dodecane, 4,6-dimethyl-

Sample AJ
Number of peaks: 121
Number of identified compounds: 87
Ratio of identified peak area: 94.79%

Peak Area [%]	Database comparison
0.3	Decane, 3,3,8-trimethyl-
0.34	Dodecane
0.4	Octane, 6-ethyl-2-methyl-
0.45	Oxygen
0.47	Benzenamine, 2,3,4,5,6-pentafluoro-
0.48	2- Bromopropionic acid, octyl ester
0.51	1-Undecene, 7-methyl-
0.51	Decane, 3,3,6-trimethyl-
0.58	Dodecane, 4,6-dimethyl-
0.59	Decane, 1-iodo-
0.6	Dodecane, 4,6-dimethyl-
0.7	Cyclotetrasiloxane, octamethyl-
0.77	Heptadecane
0.77	Undecane, 3,8-dimethyl-
0.78	Heneicosane
0.84	Heptasiloxane, hexadecamethyl-
0.85	Eicosane
0.9	Hexasiloxane, tetradecamethyl-
1	Octane, 2,3,6,7-tetramethyl-
1.07	Isotridecanol-
1.13	Cyclopentasiloxane, decamethyl-
1.18	Dodecane, 4,6-dimethyl-
1.2	Undecane, 4,8-dimethyl-
1.21	Dodecane, 4,6-dimethyl-
1.22	Dodecane, 4,6-dimethyl-
1.4	Dodecane, 4,6-dimethyl-
1.5	Dodecane, 4,6-dimethyl-
1.56	Octane, 5-ethyl-2-methyl-
1.64	Dodecane, 4,6-dimethyl-
1.76	Nonane, 5-butyl-
1.85	Heptadecane
2	Tetradecane
2.01	Dodecane, 4,6-dimethyl-
2.12	Isotridecanol-
2.4	Dodecane, 4,6-dimethyl-
3	1-Hexadecanesulfonyl chloride
4.87	Hexadecane
5.3	Eicosane
5.46	1-Undecene, 7-methyl-
5.68	Dodecane, 4,6-dimethyl-

**5.72**	Isotridecanol-
**6.58**	Eicosane
**7.65**	1-Undecene, 7-methyl-
**12.54**	Hexadecane

Sample AK
Number of peaks: 102
Number of identified compounds: 76
Ratio of identified peak area: 96.92%

Peak Area [%]	Database comparison
**0.05**	Oxygen
**0.07**	Nonane, 5-butyl-
**0.08**	2-Bromotetradecane
**0.1**	3,5-Dimethyldodecane
**0.12**	Caprolactam
**0.12**	o-Xylene
**0.13**	Tetradecane, 4-methyl-
**0.14**	Undecane, 3-methyl-
**0.15**	2-Isopropyl-5-methyl-1-heptanol
**0.15**	7-Tetradecene, (Z)-
**0.17**	Heptane, 2,5,5-trimethyl-
**0.17**	Octadecane, 1-(ethenyloxy)-
**0.19**	6-Tridecanol, 3,9-diethyl-
**0.2**	Eicosane
**0.2**	Tridecane, 4-methyl-
**0.24**	1-Octanol, 2-methyl-
**0.24**	3-Octadecene, (E)-
**0.24**	Tetradecane, 4-methyl-
**0.25**	Cyclopentasiloxane, decamethyl-
**0.26**	1,2,4-Methenoazulene, decahydro-1,5,5,8a-tetramethyl-, [1S-(1.alpha.,2.alpha.,3a.beta.,4.alpha.,8a.beta.,9R*)]-
**0.31**	1-Methoxy-2-propyl acetate
**0.31**	3-Hexadecene, (Z)-
**0.31**	Octane, 2,6-dimethyl-
**0.32**	Decane, 5-propyl-
**0.33**	Dodecane, 4,6-dimethyl-
**0.34**	Hexadecane, 1-iodo-
**0.34**	Hexadecane, 1-iodo-
**0.36**	Isotridecanol-
**0.36**	Tetradecane, 4-methyl-
**0.37**	Dodecane, 4,6-dimethyl-
**0.41**	Dodecane, 4,6-dimethyl-
**0.41**	Octadecane
**0.41**	Tetradecane, 5-methyl-
**0.5**	Dodecane, 4,6-dimethyl-
**0.52**	2-Bromo dodecane
**0.55**	Eicosane
**0.57**	Undecane
**0.58**	Hexasiloxane, tetradecamethyl-
**0.6**	1-Decene, 2,4-dimethyl-

EXCLI Journal 2021;20:995-1008 – ISSN 1611-2156
Received: April 08, 2021, accepted: May 26, 2021, published: June 01, 2021

**0.6**	Hexadecane
**0.61**	Decane, 5-propyl-
**0.62**	Dodecane, 4,6-dimethyl-
**0.63**	Tetradecane, 2-methyl-
**0.69**	Dodecane, 4-methyl-
**0.71**	1-Undecene, 7-methyl-
**0.85**	Octane, 2,3,6,7-tetramethyl-
**0.86**	Dodecane, 4,6-dimethyl-
**0.87**	Pentadecane, 2,6,10,14-tetramethyl-
**0.88**	Disulfide, di-tert-dodecyl
**0.94**	Decane, 1-iodo-
**0.96**	Heptasiloxane, hexadecamethyl-
**1**	Hexadecane, 2,6,11,15-tetramethyl-
**1.02**	Dodecane, 4,6-dimethyl-
**1.02**	Hexadecane
**1.09**	Hexadecane
**1.12**	1-Butanol, 3-methoxy-
**1.17**	Sulfurous acid, pentyl tridecyl ester
**1.28**	Eicosane
**1.32**	Tetradecane
**1.49**	Dodecane, 4,6-dimethyl-
**1.82**	Isotridecanol-
**1.86**	Octane, 5-ethyl-2-methyl-
**1.9**	Dodecane, 4,6-dimethyl-
**2.04**	Eicosane
**2.15**	Undecane, 5-methyl-
**2.16**	Isotridecanol-
**2.25**	Dodecane
**2.29**	Dodecane, 4,6-dimethyl-
**2.71**	1-Heptanol, 2,4-diethyl-
**3.07**	Dodecane, 4,6-dimethyl-
**3.8**	Dodecane, 4,6-dimethyl-
**3.93**	Octane, 5-ethyl-2-methyl-
**5.45**	Eicosane
**6.03**	Dodecane, 4,6-dimethyl-
**6.69**	Octane, 5-ethyl-2-methyl-
**17.87**	Dodecane, 4,6-dimethyl-

EXCLI Journal 2021;20:995-1008 – ISSN 1611-2156
Received: April 08, 2021, accepted: May 26, 2021, published: June 01, 2021

Sample AL
Number of peaks: 124
Number of identified compounds: 82
Ratio of identified peak area: 94.43%

Peak Area [%]	Database comparison
**0.06**	Cyclohexane, decyl-
**0.07**	Decane, 2,5-dimethyl-
**0.08**	Dodecane, 4-methyl-
**0.1**	Nonane, 5-(2-methylpropyl)-
**0.1**	Tetratriacontyl pentafluoropropionate
**0.11**	4-Decene, 7-methyl-, (E)-
**0.11**	Undecane, 2-methyl-
**0.12**	1-Butanol, 3-methoxy-, acetate
**0.14**	Isotridecanol-
**0.14**	Octacosane
**0.14**	Undecane, 2-cyclohexyl-
**0.15**	Hexadecane, 2,6,10,14-tetramethyl-
**0.16**	Pentadecane, 8-hexyl-
**0.17**	Tetradecane, 4-methyl-
**0.18**	Dodecane, 2-methyl-
**0.19**	Cyclododecane
**0.19**	Eicosane
**0.19**	Nonane, 5-butyl-
**0.19**	Octane, 2,3,6,7-tetramethyl-
**0.21**	Dichloroacetic acid, 6-ethyl-3-octyl ester
**0.21**	Heneicosane
**0.22**	Hexadecane
**0.25**	p-Xylene
**0.27**	1-Octanol, 2-butyl-
**0.27**	Tetradecane, 4-methyl-
**0.28**	Nonacosane
**0.3**	2-Hexyl-1-octanol
**0.31**	Dodecane, 4,6-dimethyl-
**0.31**	Eicosane
**0.32**	Octadecane
**0.33**	Dodecyl trifluoroacetate
**0.33**	Pentadecane, 2,6,10,14-tetramethyl-
**0.34**	4-Methyl-dodecan-1-ol
**0.34**	Heneicosane
**0.35**	2-Isopropyl-5-methyl-1-heptanol
**0.38**	1-Tridecene
**0.39**	1-Methoxy-2-propyl acetate
**0.41**	Decane, 2,8,8-trimethyl-
**0.43**	Tetradecane, 2-methyl-
**0.48**	Heptane, 2,5,5-trimethyl-

EXCLI Journal 2021;20:995-1008 – ISSN 1611-2156
Received: April 08, 2021, accepted: May 26, 2021, published: June 01, 2021

**0.48**	Isotridecanol-
**0.51**	Dodecane, 4,6-dimethyl-
**0.54**	Hexadecane
**0.56**	Heneicosane
**0.62**	Dichloroacetic acid, dodecyl ester
**0.79**	Dodecane, 4,6-dimethyl-
**0.79**	Isotridecanol-
**0.8**	Dodecane, 4,6-dimethyl-
**0.83**	Dodecane, 4-methyl-
**0.9**	Dodecane, 4,6-dimethyl-
**0.94**	Octane, 6-ethyl-2-methyl-
**0.97**	Tetradecane
**1.02**	Undecane
**1.14**	Octane, 2,6,6-trimethyl-
**1.15**	Dodecane, 4,6-dimethyl-
**1.18**	Dodecane, 4,6-dimethyl-
**1.2**	Dodecane, 4,6-dimethyl-
**1.25**	Dodecane, 4,6-dimethyl-
**1.41**	Nonane, 5-methyl-5-propyl-
**1.54**	Hexadecane, 2,6,11,15-tetramethyl-
**1.59**	Dodecane, 4,6-dimethyl-
**1.69**	Sulfurous acid, pentyl tridecyl ester
**1.73**	Dodecane, 4,6-dimethyl-
**1.79**	Hexadecane, 2,6,11,15-tetramethyl-
**1.81**	Ethanol, 2-(dodecyloxy)-
**1.86**	Octane, 5-ethyl-2-methyl-
**1.87**	1-Undecene, 7-methyl-
**2**	Hexadecane
**2.04**	Octane, 5-ethyl-2-methyl-
**2.05**	Dodecane, 4,6-dimethyl-
**2.16**	1-Butanol, 3-methoxy-
**2.32**	Heneicosane
**2.54**	Heptasiloxane, hexadecamethyl-
**2.56**	Dodecane, 4,6-dimethyl-
**2.76**	Hexasiloxane, tetradecamethyl-
**2.88**	Dodecane
**3.57**	Octane, 5-ethyl-2-methyl-
**4.59**	Dodecane, 4,6-dimethyl-
**5.03**	Isotridecanol-
**5.07**	1-Heptanol, 2,4-diethyl-
**7.07**	1-Heptanol, 2,4-diethyl-
**7.51**	Dodecane, 4,6-dimethyl-

Sample AM
Number of peaks: 121
Number of identified compounds: 87
Ratio of identified peak area: 94.79%

Peak Area [%]	Database comparison
0.05	1-Butanol, 3-methoxy-, acetate
0.1	Nonacosane
0.1	Undecane, 5-methyl-
0.11	Eicosane
0.13	Sulfurous acid, dodecyl hexyl ester
0.15	1-Decanol, 2-hexyl-
0.15	Hexadecane
0.16	1-Decanol, 2-methyl-
0.16	Undecane, 2-methyl-
0.18	1-Nonanol, 4,8-dimethyl-
0.18	Nonane, 5-methyl-5-propyl-
0.19	Nonane, 5-(2-methylpropyl)-
0.2	1-Octene, 3,7-dimethyl-
0.2	Nonane, 3-methyl-5-propyl-
0.21	Hexadecane
0.21	Tetradecane, 4-methyl-
0.21	Undecane, 3-methyl-
0.22	Eicosane
0.22	Heptadecane
0.22	p-Xylene
0.24	Dichloroacetic acid, 6-ethyl-3-octyl ester
0.24	Eicosane
0.27	Nonane, 2,5-dimethyl-
0.29	1-Heptanol, 2,4-diethyl-
0.29	Cyclopentane, nonyl-
0.29	Dodecane, 4,6-dimethyl-
0.29	Heptane, 3-ethyl-2-methyl-
0.35	Decane, 2,8,8-trimethyl-
0.36	Decane, 4-methyl-
0.38	1-Dodecene
0.4	1-Methoxy-2-propyl acetate
0.4	1-Tetradecanol
0.4	Pentadecane
0.42	Heneicosane
0.43	Heptadecane
0.43	Heptadecyl heptafluorobutyrate
0.43	Undecane, 2-methyl-
0.46	Eicosane
0.47	Tetradecane
0.48	Dichloroacetic acid, decyl ester

**0.53**	Dodecane, 4,6-dimethyl-
**0.56**	Dodecane, 4,6-dimethyl-
**0.56**	Isotridecanol-
**0.61**	Sulfurous acid, decyl hexyl ester
**0.65**	Decane, 4-methyl-
**0.66**	Phenol, 2,4-bis(1,1-dimethylethyl)-
**0.68**	Heptane, 2,5,5-trimethyl-
**0.69**	Hexasiloxane, tetradecamethyl-
**0.72**	Dodecane, 4,6-dimethyl-
**0.81**	Dodecane, 4,6-dimethyl-
**0.81**	Heptasiloxane, hexadecamethyl-
**0.87**	2-Isopropyl-5-methyl-1-heptanol
**0.9**	Undecyl trifluoroacetate
**0.91**	Tetradecane
**0.92**	Dodecane, 4-methyl-
**0.94**	Undecane
**0.96**	Dodecane, 4,6-dimethyl-
**0.96**	Dodecane, 4,6-dimethyl-
**1**	Dodecane, 4,6-dimethyl-
**1.1**	Dodecane, 4,6-dimethyl-
**1.17**	Decane, 3,3,6-trimethyl-
**1.2**	Dodecane, 4,6-dimethyl-
**1.2**	Sulfurous acid, pentyl tridecyl ester
**1.24**	Dodecane, 4,6-dimethyl-
**1.33**	Dodecane, 2,7,10-trimethyl-
**1.34**	Octane, 2,3,6,7-tetramethyl-
**1.35**	Dodecane, 4,6-dimethyl-
**1.39**	Eicosane
**1.4**	Octane, 5-ethyl-2-methyl-
**1.5**	Eicosane
**1.64**	Tetradecane, 5-methyl-
**1.74**	Octane, 2,3,6,7-tetramethyl-
**1.83**	1-Octanol, 2,7-dimethyl-
**1.9**	1-Undecene, 7-methyl-
**2.01**	1-Butanol, 3-methoxy-
**2.04**	Dodecane, 4,6-dimethyl-
**2.21**	Undecane, 2,5-dimethyl-
**2.26**	Eicosane
**2.46**	Dodecane, 4,6-dimethyl-
**3.39**	Octane, 5-ethyl-2-methyl-
**3.56**	Dodecane
**3.56**	Dodecane, 4,6-dimethyl-
**3.96**	2-Isopropyl-5-methyl-1-heptanol
**4.02**	Octane, 5-ethyl-2-methyl-
**4.14**	Isotridecanol-

EXCLI Journal 2021;20:995-1008 – ISSN 1611-2156
Received: April 08, 2021, accepted: May 26, 2021, published: June 01, 2021

**5.41**	1-Heptanol, 2,4-diethyl-
**9.03**	Dodecane, 4,6-dimethyl-

EXCLI Journal 2021;20:995-1008 – ISSN 1611-2156
Received: April 08, 2021, accepted: May 26, 2021, published: June 01, 2021

Sample AN
Number of peaks: 101
Number of identified compounds: 75
Ratio of identified peak area: 97.54%

Peak Area [%]	Database comparison
**0.06**	1-Butanol, 3-methoxy-
**0.07**	1-Undecene, 7-methyl-
**0.08**	Eicosane
**0.09**	Hexadecane, 1,1-bis(dodecyloxy)-
**0.1**	2,5-Cyclohexadiene-1,4-dione, 2,6-bis(1,1-dimethylethyl)-
**0.1**	Decane
**0.12**	7-Tetradecene
**0.13**	2,3-Dimethyldodecane
**0.13**	Dodecane, 2,6,10-trimethyl-
**0.13**	p-Xylene
**0.14**	Decane, 2,3,5-trimethyl-
**0.15**	Hexadecane
**0.15**	Nonacosane
**0.15**	Octadecane
**0.16**	Nonane, 5-methyl-5-propyl-
**0.16**	Undecane, 3,7-dimethyl-
**0.18**	Undecane, 3,4-dimethyl-
**0.19**	Tetradecane, 4-methyl-
**0.19**	Undecane, 3,4-dimethyl-
**0.21**	2-Ethylhexyl methacrylate
**0.23**	2-Undecene, 2,5-dimethyl-
**0.25**	Nonane, 5-methyl-5-propyl-
**0.27**	10-Methylnonadecane
**0.27**	Pentadecane, 2,6,10,14-tetramethyl-
**0.31**	Dodecane, 4,6-dimethyl-
**0.32**	1-Heptanol, 2,4-diethyl-
**0.34**	Decane, 5-propyl-
**0.34**	Heptadecane, 8-methyl-
**0.37**	4-Methyl-dodecan-1-ol
**0.4**	Heneicosane
**0.4**	Tetradecane, 2-methyl-
**0.41**	1-Tridecanol
**0.42**	Octacosyl trifluoroacetate
**0.43**	Undecane
**0.45**	Dodecane, 4,6-dimethyl-
**0.45**	Hexadecane, 2,6,11,15-tetramethyl-
**0.49**	Hexasiloxane, tetradecamethyl-
**0.53**	Caprolactam
**0.53**	Dodecane, 4-methyl-
**0.55**	Phenol, 2,4-bis(1,1-dimethylethyl)-

EXCLI Journal 2021;20:995-1008 – ISSN 1611-2156
Received: April 08, 2021, accepted: May 26, 2021, published: June 01, 2021

**0.68**	Dodecane, 4,6-dimethyl-
**0.7**	Dodecane, 4,6-dimethyl-
**0.72**	Octacosyl trifluoroacetate
**0.81**	4-Decene, 7-methyl-, (E)-
**0.94**	Dodecane, 4,6-dimethyl-
**0.98**	1-Hexadecanesulfonyl chloride
**0.98**	Dodecane, 4,6-dimethyl-
**1.02**	Heptasiloxane, hexadecamethyl-
**1.05**	Tetradecane
**1.06**	Sulfurous acid, pentyl tridecyl ester
**1.07**	Dodecane, 4,6-dimethyl-
**1.19**	Dodecane, 4,6-dimethyl-
**1.3**	Hexadecane
**1.32**	Heneicosane
**1.34**	Dodecane, 4,6-dimethyl-
**1.36**	Dodecane, 4,6-dimethyl-
**1.41**	Eicosane
**1.45**	Dodecane, 4,6-dimethyl-
**1.52**	Dodecane
**1.6**	Octane, 2,3,6,7-tetramethyl-
**2**	Octane, 5-ethyl-2-methyl-
**2.05**	Octane, 5-ethyl-2-methyl-
**2.27**	Dodecane, 4,6-dimethyl-
**3.05**	Heptane, 2,5,5-trimethyl-
**3.09**	Dodecane, 4,6-dimethyl-
**3.09**	Dodecane, 4,6-dimethyl-
**3.7**	Heptane, 2,5,5-trimethyl-
**3.98**	Octane, 5-ethyl-2-methyl-
**4.29**	Octane, 5-ethyl-2-methyl-
**5.54**	1-Undecene, 7-methyl-
**5.63**	2-Isopropyl-5-methyl-1-heptanol
**5.67**	1-Heptanol, 2,4-diethyl-
**5.71**	1-Undecene, 7-methyl-
**6.49**	Hexadecane
**8.03**	Isotridecanol-

EXCLI Journal 2021;20:995-1008 – ISSN 1611-2156
Received: April 08, 2021, accepted: May 26, 2021, published: June 01, 2021

Sample AO
Number of peaks: 107
Number of identified compounds: 80
Ratio of identified peak area: 96.11%

Peak Area [%]	Database comparison
0.06	Dodecane, 4,6-dimethyl-
0.07	Sulfurous acid, dodecyl hexyl ester
0.08	Undecane, 3-methyl-
0.09	1-Octene, 3,7-dimethyl-
0.1	Nonadecyl trifluoroacetate
0.11	1-Butanol, 3-methoxy-, acetate
0.13	2-Isopropyl-5-methyl-1-heptanol
0.13	Hexadecane, 2,6,11,15-tetramethyl-
0.18	Cyclopentasiloxane, decamethyl-
0.18	Heneicosane
0.18	Tetratetracontane
0.18	Tetratetracontane
0.19	2,2,4,4-Tetramethyloctane
0.19	Octacosyl heptafluorobutyrate
0.21	Heneicosane
0.23	2,5-di-tert-Butyl-1,4-benzoquinone
0.24	Tetradecane, 4-methyl-
0.25	2-Isopropyl-5-methyl-1-heptanol
0.26	2-Isopropyl-5-methyl-1-heptanol
0.29	Eicosane
0.29	Nonane, 3-methyl-5-propyl-
0.29	p-Xylene
0.33	1-Decanol, 2-hexyl-
0.34	Eicosane
0.35	2-Undecene, 2,5-dimethyl-
0.36	Decane, 5-propyl-
0.41	Heptane, 2,5,5-trimethyl-
0.41	Tetradecane, 3-methyl-
0.42	Octane, 2,6,6-trimethyl-
0.43	Hexadecane, 1,1-bis(dodecyloxy)-
0.48	Hexadecane, 2,6,11,15-tetramethyl-
0.49	Hexadecane, 1-iodo-
0.51	Dodecane, 4,6-dimethyl-
0.54	Tetradecane, 2,5-dimethyl-
0.57	Undecane, 5-methyl-
0.6	1-Heptanol, 2,4-diethyl-
0.6	Ethanone, 1,1'-(1,4-phenylene)bis-
0.67	Eicosane
0.7	Decane, 2,8,8-trimethyl-
0.73	Dodecane, 4,6-dimethyl-

EXCLI Journal 2021;20:995-1008 – ISSN 1611-2156
Received: April 08, 2021, accepted: May 26, 2021, published: June 01, 2021

**0.76**	1-Decene, 2,4-dimethyl-
**0.79**	Heptasiloxane, hexadecamethyl-
**0.82**	Dodecane, 4,6-dimethyl-
**0.86**	Heptasiloxane, hexadecamethyl-
**0.86**	Octane, 5-ethyl-2-methyl-
**0.87**	1-Methoxy-2-propyl acetate
**0.88**	1-Undecene, 7-methyl-
**0.95**	Dodecane, 4,6-dimethyl-
**0.95**	Dodecane, 4,6-dimethyl-
**0.96**	Tetradecane
**1.04**	Dodecane, 4,6-dimethyl-
**1.05**	Heptadecane, 8-methyl-
**1.07**	Cyclopentanone, 2-methyl-
**1.21**	1-Hexadecanesulfonyl chloride
**1.3**	Dodecane, 4,6-dimethyl-
**1.33**	Dodecane, 4,6-dimethyl-
**1.4**	Dodecane, 4,6-dimethyl-
**1.44**	Undecane
**1.47**	Isotridecanol-
**1.5**	Dodecane, 4,6-dimethyl-
**1.51**	Dodecane, 4,6-dimethyl-
**1.53**	Octane, 5-ethyl-2-methyl-
**1.62**	Caprolactam
**1.63**	Dodecane, 4,6-dimethyl-
**1.75**	Octane, 5-ethyl-2-methyl-
**1.77**	Dodecane, 4,6-dimethyl-
**1.98**	Dodecane, 4,6-dimethyl-
**2.05**	Decane, 2,2-dimethyl-
**2.15**	2-Isopropyl-5-methyl-1-heptanol
**2.25**	Dodecane
**2.73**	1-Butanol, 3-methoxy-
**2.9**	Octacosane
**3.04**	Phenol, 2,4-bis(1,1-dimethylethyl)-
**3.52**	Isotridecanol-
**3.59**	Eicosane
**3.91**	1-Heptanol, 2,4-diethyl-
**4.14**	Eicosane
**4.85**	1-Heptanol, 2,4-diethyl-
**6.03**	Dodecane, 4,6-dimethyl-
**8.78**	Dodecane, 4,6-dimethyl-

Sample AP
Number of peaks: 115
Number of identified compounds: 76
Ratio of identified peak area: 91.66%

Peak Area [%]	Database comparison
**0.13**	Cyclotetrasiloxane, octamethyl-
**0.15**	Heneicosane
**0.15**	Heptadecane, 8-methyl-
**0.17**	Hexasiloxane, tetradecamethyl-
**0.17**	Nonane, 5-(2-methylpropyl)-
**0.18**	1-Butanol, 3-methoxy-, acetate
**0.18**	2,5-Cyclohexadiene-1,4-dione, 2,6-bis(1,1-dimethylethyl)-
**0.18**	Octacosane
**0.2**	2,3-Dimethyldecane
**0.2**	Decane, 3-ethyl-3-methyl-
**0.21**	1-Dodecene
**0.21**	Pentatriacontane
**0.22**	Heptane, 3,3,4-trimethyl-
**0.22**	Hexadecane, 2,6,10,14-tetramethyl-
**0.25**	Eicosane
**0.26**	Dodecane, 2,6,11-trimethyl-
**0.26**	Dodecane, 4-methyl-
**0.3**	Pentadecane, 2,6,10,14-tetramethyl-
**0.31**	Hexadecane, 2,6,10,14-tetramethyl-
**0.33**	Undecane, 3-methyl-
**0.34**	Octane, 2,3,6-trimethyl-
**0.34**	Undecane, 2,5-dimethyl-
**0.35**	Tetradecanal
**0.39**	Decane, 3,3,8-trimethyl-
**0.39**	Dodecane, 1-iodo-
**0.41**	Tetracosane
**0.43**	Cyclopentane, decyl-
**0.44**	1-Tridecene
**0.44**	Nonane, 5-methyl-5-propyl-
**0.45**	1-Decene, 3,3,4-trimethyl-
**0.49**	Isotridecanol-
**0.51**	Dodecane, 4,6-dimethyl-
**0.52**	Heneicosane, 11-(1-ethylpropyl)-
**0.61**	Eicosane
**0.62**	2-Undecene, 7-methyl-
**0.65**	Tetradecane, 2-methyl-
**0.66**	Octane, 2,3,6,7-tetramethyl-
**0.67**	1-Undecene, 9-methyl-
**0.69**	Dodecane, 4,6-dimethyl-
**0.71**	Octane, 6-ethyl-2-methyl-

EXCLI Journal 2021;20:995-1008 – ISSN 1611-2156
Received: April 08, 2021, accepted: May 26, 2021, published: June 01, 2021

**0.73**	1-Undecene, 7-methyl-
**0.77**	Decanal
**0.78**	Phenol, 2,4-bis(1,1-dimethylethyl)-
**0.81**	Dodecane, 4,6-dimethyl-
**0.83**	Dodecane, 4,6-dimethyl-
**0.84**	1-Methoxy-2-propyl acetate
**0.88**	3-Hexadecene, (Z)-
**0.88**	Isotridecanol-
**0.91**	2,2,4,4-Tetramethyloctane
**0.96**	Dodecane, 4,6-dimethyl-
**1.05**	Dodecane, 4,6-dimethyl-
**1.1**	Nonane, 5-methyl-5-propyl-
**1.18**	Dodecane, 4-methyl-
**1.18**	Sulfurous acid, pentyl tridecyl ester
**1.24**	Octane, 5-ethyl-2-methyl-
**1.26**	Dodecane, 4,6-dimethyl-
**1.31**	Dodecane, 4,6-dimethyl-
**1.32**	Nonane, 5-(2-methylpropyl)-
**1.37**	2-Isopropyl-5-methyl-1-heptanol
**1.38**	Tetradecane
**1.4**	Undecane, 5-methyl-
**1.51**	Dodecane, 4,6-dimethyl-
**1.78**	Dodecane, 4,6-dimethyl-
**1.92**	Undecane
**1.97**	Heptasiloxane, hexadecamethyl-
**2.02**	Eicosane
**2.25**	Eicosane
**2.92**	Eicosane
**3.4**	1-Butanol, 3-methoxy-
**3.82**	Dodecane, 4,6-dimethyl-
**3.88**	Isotridecanol-
**3.96**	1-Heptanol, 2,4-diethyl-
**4.93**	Dodecane
**5.33**	1-Heptanol, 2,4-diethyl-
**5.33**	Decane, 2,2-dimethyl-
**10.07**	Hexadecane

EXCLI Journal 2021;20:995-1008 – ISSN 1611-2156
Received: April 08, 2021, accepted: May 26, 2021, published: June 01, 2021

Sample AQ
Number of peaks: 106
Number of identified compounds: 76
Ratio of identified peak area: 95.57%

Peak Area [%]	Database comparison
**0.09**	Decane, 3,3,4-trimethyl-
**0.09**	Oxygen
**0.1**	Decane, 2,3,8-trimethyl-
**0.11**	Oxalic acid, bis(6-ethyloct-3-yl) ester
**0.12**	Cyclododecane
**0.14**	Cyclopentasiloxane, decamethyl-
**0.16**	2,5-Cyclohexadiene-1,4-dione, 2,6-bis(1,1-dimethylethyl)-
**0.17**	Nonacosane
**0.18**	Dichloroacetic acid, 6-ethyl-3-octyl ester
**0.18**	Heneicosane
**0.19**	Nonane, 3-methyl-
**0.2**	Decane, 3-ethyl-3-methyl-
**0.21**	1-Hexadecanesulfonyl chloride
**0.21**	1-Octanol, 2,7-dimethyl-
**0.21**	Pentadecane, 2,6,10,14-tetramethyl-
**0.21**	p-Xylene
**0.25**	Dodecanal
**0.25**	Eicosane
**0.26**	Decanal
**0.26**	Octane, 5-ethyl-2-methyl-
**0.3**	Tetratriacontane
**0.31**	Heneicosane
**0.36**	Tetradecane, 4-methyl-
**0.37**	Benzene, 3-cyclohexen-1-yl-
**0.38**	1-Undecene, 9-methyl-
**0.38**	Heptafluorobutanoic acid, heptadecyl ester
**0.38**	Hexadecane
**0.4**	3-Eicosene, (E)-
**0.41**	Caprolactam
**0.43**	Octane, 2,6,6-trimethyl-
**0.51**	2-Bromo dodecane
**0.52**	Decane, 5-propyl-
**0.55**	Hexasiloxane, tetradecamethyl-
**0.56**	Heptadecyl heptafluorobutyrate
**0.58**	Hexadecane, 2,6,10,14-tetramethyl-
**0.59**	Nonane, 2,6-dimethyl-
**0.59**	Pentadecane, 7-methyl-
**0.62**	Dodecane, 4,6-dimethyl-
**0.62**	Octane, 5-ethyl-2-methyl-
**0.65**	1-Decene, 2,4-dimethyl-

EXCLI Journal 2021;20:995-1008 – ISSN 1611-2156
Received: April 08, 2021, accepted: May 26, 2021, published: June 01, 2021

**0.65**	1-Methoxy-2-propyl acetate
**0.65**	Undecane
**0.72**	1-Undecene, 7-methyl-
**0.77**	Dodecane, 4,6-dimethyl-
**0.83**	Dodecane, 4,6-dimethyl-
**0.88**	Isotridecanol-
**0.9**	Dodecane, 4-methyl-
**0.91**	Phenol, 2,5-bis(1,1-dimethylethyl)-
**0.93**	1-Butanol, 3-methoxy-
**0.98**	Octane, 6-ethyl-2-methyl-
**0.98**	Tetradecane
**0.99**	Nonane, 5-methyl-5-propyl-
**1.1**	Heptasiloxane, hexadecamethyl-
**1.19**	Dodecane, 4,6-dimethyl-
**1.37**	Dodecane, 4,6-dimethyl-
**1.38**	Dodecane, 4,6-dimethyl-
**1.38**	Dodecane, 4,6-dimethyl-
**1.39**	Dodecane, 4,6-dimethyl-
**1.44**	1-Heptanol, 2,4-diethyl-
**1.59**	Dodecane, 4,6-dimethyl-
**1.65**	Sulfurous acid, pentyl tridecyl ester
**1.84**	Dodecane, 4,6-dimethyl-
**2.03**	Dodecane, 4,6-dimethyl-
**2.08**	Propanoic acid, 2-methyl-, 1-(1,1-dimethylethyl)-2-methyl-1,3-propanediyl ester
**2.24**	Eicosane
**2.26**	1-Hexadecanesulfonyl chloride
**2.33**	Dodecane, 4,6-dimethyl-
**2.48**	Dodecane
**2.81**	Octacosane
**3.04**	Eicosane
**3.28**	Heptasiloxane, hexadecamethyl-
**4.17**	Dodecane, 4,6-dimethyl-
**6.12**	Hexadecane
**7.51**	2-Isopropyl-5-methyl-1-heptanol
**7.63**	Isotridecanol-
**9.97**	Isotridecanol-

Sample AR
Number of peaks: 68
Number of identified compounds: 49
Ratio of identified peak area: 92.15%

Peak Area [%]	Database comparison
0.23	1-Nonanol, 4,8-dimethyl-
0.28	Dodecane
0.31	Decane, 3,3,8-trimethyl-
0.31	Heptadecane
0.32	2-Isopropyl-5-methyl-1-heptanol
0.33	Cyclotetrasiloxane, octamethyl-
0.33	Dodecane, 4,6-dimethyl-
0.4	Dodecane, 4,6-dimethyl-
0.58	Heptadecane
0.61	1-Hexadecanesulfonyl chloride
0.62	Dodecane, 4,6-dimethyl-
0.63	Dodecane, 4,6-dimethyl-
0.65	Hexasiloxane, tetradecamethyl-
0.66	Decanal
0.66	Dodecane, 4,6-dimethyl-
0.68	Cyclopentane, 1-pentyl-2-propyl-
0.73	Dodecane, 4,6-dimethyl-
0.77	Dodecane, 4,6-dimethyl-
0.77	Eicosane
0.79	Dodecane, 4,6-dimethyl-
0.85	Cyclopentasiloxane, decamethyl-
0.87	Dodecane, 4-methyl-
0.87	Eicosane
0.98	Octane, 5-ethyl-2-methyl-
1.02	Octane, 2,3,6,7-tetramethyl-
1.03	Caprolactam
1.12	Decane, 3,3,6-trimethyl-
1.2	Tetradecane
1.5	Eicosane
1.52	Dodecane, 4,6-dimethyl-
1.54	Dodecane, 4,6-dimethyl-
1.63	Dodecane, 4,6-dimethyl-
1.74	Eicosane
1.94	Eicosane
1.97	Isotridecanol-
2.09	Heptasiloxane, hexadecamethyl-
2.09	Isotridecanol-
2.16	Dodecane, 4,6-dimethyl-
2.16	Octane, 5-ethyl-2-methyl-
2.38	Dodecane, 4,6-dimethyl-

EXCLI Journal 2021;20:995-1008 – ISSN 1611-2156
Received: April 08, 2021, accepted: May 26, 2021, published: June 01, 2021

**2.53**	Octane, 6-ethyl-2-methyl-
**2.71**	Dodecane, 4,6-dimethyl-
**2.73**	Hexadecane
**2.75**	Propanoic acid, 2-methyl-, 1-(1,1-dimethylethyl)-2-methyl-1,3-propanediyl ester
**2.76**	1-Undecene, 7-methyl-
**6.18**	Hexadecane
**8.04**	Dodecane, 4,6-dimethyl-
**8.81**	Eicosane
**14.32**	Hexadecane

Sample AS
Number of peaks: 91
Number of identified compounds: 69
Ratio of identified peak area: 95.92%

Peak Area [%]	Database comparison
0.12	Cyclopentasiloxane, decamethyl-
0.16	Oxygen
0.18	2,3-Dimethyldodecane
0.18	Propanoic acid, 2-methyl-, 1-(1,1-dimethylethyl)-2-methyl-1,3-propanediyl ester
0.21	2-Isopropyl-5-methyl-1-heptanol
0.21	Heptane, 2,5,5-trimethyl-
0.23	Eicosane, 2-methyl-
0.24	Dodecane, 4-methyl-
0.25	Dotriacontane
0.27	1-Hexadecanesulfonyl chloride
0.31	2-Undecene, 2,5-dimethyl-
0.31	Sulfurous acid, hexyl octyl ester
0.33	Dodecane, 4,6-dimethyl-
0.37	Eicosane
0.4	Heneicosane
0.4	o-Xylene
0.41	Tetradecane, 4-methyl-
0.45	Tetradecane, 5-methyl-
0.48	Heptadecane
0.49	Hexasiloxane, tetradecamethyl-
0.49	Undecane, 5-methyl-
0.52	1-Tridecene
0.53	Nonane, 2-methyl-5-propyl-
0.59	Octane, 5-ethyl-2-methyl-
0.61	1-Methoxy-2-propyl acetate
0.66	Heptadecyl heptafluorobutyrate
0.67	Decane, 2,8,8-trimethyl-
0.7	3,5-Dimethyldodecane
0.7	Pentadecane, 2,6,10,14-tetramethyl-
0.7	Undecane, 2,6-dimethyl-
0.73	Undecane
0.77	1,3-Dioxan-5-ol, 4,4,5-trimethyl-
0.82	Dodecane, 4-methyl-
0.91	Decane, 3,3,8-trimethyl-
0.98	Eicosane
1	Phenol, 2,4-bis(1,1-dimethylethyl)-
1.03	2-Undecene, 2,5-dimethyl-
1.06	Dodecane, 4,6-dimethyl-
1.07	Dodecane, 4,6-dimethyl-

EXCLI Journal 2021;20:995-1008 – ISSN 1611-2156
Received: April 08, 2021, accepted: May 26, 2021, published: June 01, 2021

**1.09**	Caprolactam
**1.11**	Isotridecanol-
**1.12**	1-Undecene, 7-methyl-
**1.12**	Octane, 5-ethyl-2-methyl-
**1.13**	Octane, 2,3,6,7-tetramethyl-
**1.25**	Cyclopentanone, 2-methyl-
**1.32**	Dodecane, 4,6-dimethyl-
**1.32**	Nonane, 5-methyl-5-propyl-
**1.39**	Sulfurous acid, decyl 2-pentyl ester
**1.42**	Nonane, 5-methyl-5-propyl-
**1.43**	Dodecane, 4,6-dimethyl-
**1.51**	Isotridecanol-
**1.56**	Dodecane, 4,6-dimethyl-
**1.59**	Tetradecane
**1.64**	Dodecane, 4,6-dimethyl-
**1.82**	Dodecane, 4,6-dimethyl-
**2.04**	Dodecane
**2.08**	1-Hexadecanesulfonyl chloride
**2.12**	Dodecane, 4,6-dimethyl-
**2.21**	Heptasiloxane, hexadecamethyl-
**2.33**	Dodecane, 4,6-dimethyl-
**2.73**	Eicosane
**2.81**	Eicosane
**2.81**	Heptasiloxane, hexadecamethyl-
**3.22**	Eicosane
**3.97**	Dodecane, 4,6-dimethyl-
**5.54**	Isotridecanol-
**6.06**	2-Isopropyl-5-methyl-1-heptanol
**7.48**	Hexadecane
**8.16**	1-Heptanol, 2,4-diethyl-

EXCLI Journal 2021;20:995-1008 – ISSN 1611-2156
Received: April 08, 2021, accepted: May 26, 2021, published: June 01, 2021

Sample AT
Number of peaks: 97
Number of identified compounds: 70
Ratio of identified peak area: 95.29%

Peak Area [%]	Database comparison
0.09	Oxygen
0.09	Undecane, 6,6-dimethyl-
0.14	Cyclopropane, nonyl-
0.14	Heptadecane
0.14	Pentadecane, 7-methyl-
0.15	2,5-Cyclohexadiene-1,4-dione, 2,6-bis(1,1-dimethylethyl)-
0.15	5-Undecene, 3-methyl-, (E)-
0.17	Eicosane
0.19	Cyclopentasiloxane, decamethyl-
0.19	Octane, 6-ethyl-2-methyl-
0.22	Decane, 3,6-dimethyl-
0.22	Hexasiloxane, tetradecamethyl-
0.23	Dodecane, 4-methyl-
0.23	Hexadecane, 1-iodo-
0.24	Nonane, 5-methyl-5-propyl-
0.25	1-Undecene, 7-methyl-
0.25	Hexadecane, 1-iodo-
0.27	1-Undecene, 7-methyl-
0.28	Dodecane, 2,7,10-trimethyl-
0.29	Decane, 3,3,8-trimethyl-
0.3	Cyclopentane, nonyl-
0.34	Heneicosane
0.34	Octane, 6-ethyl-2-methyl-
0.37	Eicosane
0.39	Decane, 2,8,8-trimethyl-
0.39	Undecyl trifluoroacetate
0.42	1-Hexadecanesulfonyl chloride
0.42	Heptadecane
0.43	Eicosane
0.48	Octane, 5-ethyl-2-methyl-
0.49	1-Butanol, 3-methoxy-, acetate
0.52	2-Undecene, 2,5-dimethyl-
0.64	2-Bromo dodecane
0.66	Octane, 5-ethyl-2-methyl-
0.71	Dodecane, 4,6-dimethyl-
0.75	p-Xylene
0.79	Dodecane, 4,6-dimethyl-
0.79	Heptasiloxane, hexadecamethyl-
0.8	Dodecane, 4,6-dimethyl-
0.85	1-Decanol, 2-methyl-

EXCLI Journal 2021;20:995-1008 – ISSN 1611-2156
Received: April 08, 2021, accepted: May 26, 2021, published: June 01, 2021

**0.88**	Dodecane, 4,6-dimethyl-
**0.88**	Isotridecanol-
**0.89**	Eicosane
**0.94**	Sulfurous acid, dodecyl pentyl ester
**0.97**	2,2,4,4-Tetramethyloctane
**0.99**	Caprolactam
**1.01**	Dodecane, 4,6-dimethyl-
**1.02**	Dodecane, 4,6-dimethyl-
**1.16**	Tetradecane
**1.25**	1-Decanol, 2-hexyl-
**1.41**	Sulfurous acid, pentadecyl pentyl ester
**1.56**	Dodecane, 4,6-dimethyl-
**1.6**	Dodecane, 4,6-dimethyl-
**1.78**	Undecane
**1.81**	Nonane, 5-methyl-5-propyl-
**1.99**	1-Methoxy-2-propyl acetate
**2.19**	1-Tetradecene
**2.27**	Eicosane
**2.37**	Dodecane, 4,6-dimethyl-
**2.42**	Nonane, 5-methyl-5-propyl-
**2.66**	Eicosane
**3.59**	Eicosane
**3.72**	Isotridecanol-
**3.75**	1-Heptanol, 2,4-diethyl-
**3.91**	1-Butanol, 3-methoxy-
**4.17**	Dodecane
**4.8**	1-Heptanol, 2,4-diethyl-
**5.97**	Dodecane, 4,6-dimethyl-
**5.99**	Decane, 2,2-dimethyl-
**12.53**	Hexadecane

EXCLI Journal 2021;20:995-1008 – ISSN 1611-2156
Received: April 08, 2021, accepted: May 26, 2021, published: June 01, 2021

Sample AU
Number of peaks: 108
Number of identified compounds: 73
Ratio of identified peak area: 93.78%

Peak Area [%]	Database comparison
**0.05**	Heneicosane, 10-methyl-
**0.07**	1-Octanol, 2-butyl-
**0.08**	1-Butanol, 3-methoxy-, acetate
**0.1**	Docosane
**0.1**	Eicosane
**0.13**	Ditetradecyl ether
**0.14**	2,2,4,4-Tetramethyloctane
**0.14**	Hexadecane, 2,6,10,14-tetramethyl-
**0.15**	2,3-Dimethyldodecane
**0.17**	Dodecane, 4-methyl-
**0.21**	Nonacosane
**0.22**	Tetradecane, 4-methyl-
**0.22**	Undecyl trifluoroacetate
**0.23**	Cyclopentasiloxane, decamethyl-
**0.24**	Hexadecane, 1-iodo-
**0.25**	Decane, 2,8,8-trimethyl-
**0.26**	Heptane, 3,3,4-trimethyl-
**0.26**	Octadecane
**0.28**	Eicosane
**0.29**	1-Octanol, 2-methyl-
**0.29**	Tetradecane, 2-methyl-
**0.33**	Tetradecane, 3-methyl-
**0.33**	Undecane, 3-methyl-
**0.34**	Cyclopentane, undecyl-
**0.34**	Pentadecane, 7-methyl-
**0.35**	Heneicosane
**0.35**	Nonane, 5-propyl-
**0.38**	Decane, 2,8,8-trimethyl-
**0.41**	Eicosane
**0.42**	Isotridecanol-
**0.44**	Decane, 2,8,8-trimethyl-
**0.47**	Dodecane, 1-iodo-
**0.48**	Undecane
**0.5**	Dodecane, 4,6-dimethyl-
**0.5**	Dodecane, 4,6-dimethyl-
**0.52**	1-Methoxy-2-propyl acetate
**0.52**	2-Undecene, 2,5-dimethyl-
**0.52**	Decane, 3,3,5-trimethyl-
**0.55**	1-Tridecene
**0.6**	2-Bromo dodecane

EXCLI Journal 2021;20:995-1008 – ISSN 1611-2156
Received: April 08, 2021, accepted: May 26, 2021, published: June 01, 2021

**0.63**	Dodecane, 4,6-dimethyl-
**0.64**	Eicosane
**0.67**	1-Undecene, 7-methyl-
**0.67**	Dodecane, 2,6,11-trimethyl-
**0.77**	Octane, 2,6,6-trimethyl-
**0.85**	1-Butanol, 3-methoxy-
**0.9**	Heneicosane
**1.01**	Tetradecane
**1.07**	Octane, 5-ethyl-2-methyl-
**1.09**	Pentadecane, 8-hexyl-
**1.11**	Dodecane, 4,6-dimethyl-
**1.23**	Sulfurous acid, pentyl tridecyl ester
**1.55**	Decane, 2,2-dimethyl-
**1.56**	Eicosane
**1.7**	Dodecane, 4,6-dimethyl-
**1.72**	Dodecane, 4,6-dimethyl-
**2.01**	Isotridecanol-
**2.11**	1-Heptanol, 2,4-diethyl-
**2.18**	Octane, 5-ethyl-2-methyl-
**2.21**	Dodecane, 4,6-dimethyl-
**2.34**	Dodecane, 4,6-dimethyl-
**2.36**	Heptasiloxane, hexadecamethyl-
**2.64**	Dodecane, 4,6-dimethyl-
**2.84**	Caprolactam
**2.91**	Isotridecanol-
**2.95**	Hexasiloxane, tetradecamethyl-
**2.99**	Octane, 5-ethyl-2-methyl-
**3.03**	Dodecane
**3.04**	Eicosane
**4.19**	Octane, 5-ethyl-2-methyl-
**4.39**	Eicosane
**7.69**	Dodecane, 4,6-dimethyl-
**14.5**	Dodecane, 4,6-dimethyl-

EXCLI Journal 2021;20:995-1008 – ISSN 1611-2156
Received: April 08, 2021, accepted: May 26, 2021, published: June 01, 2021

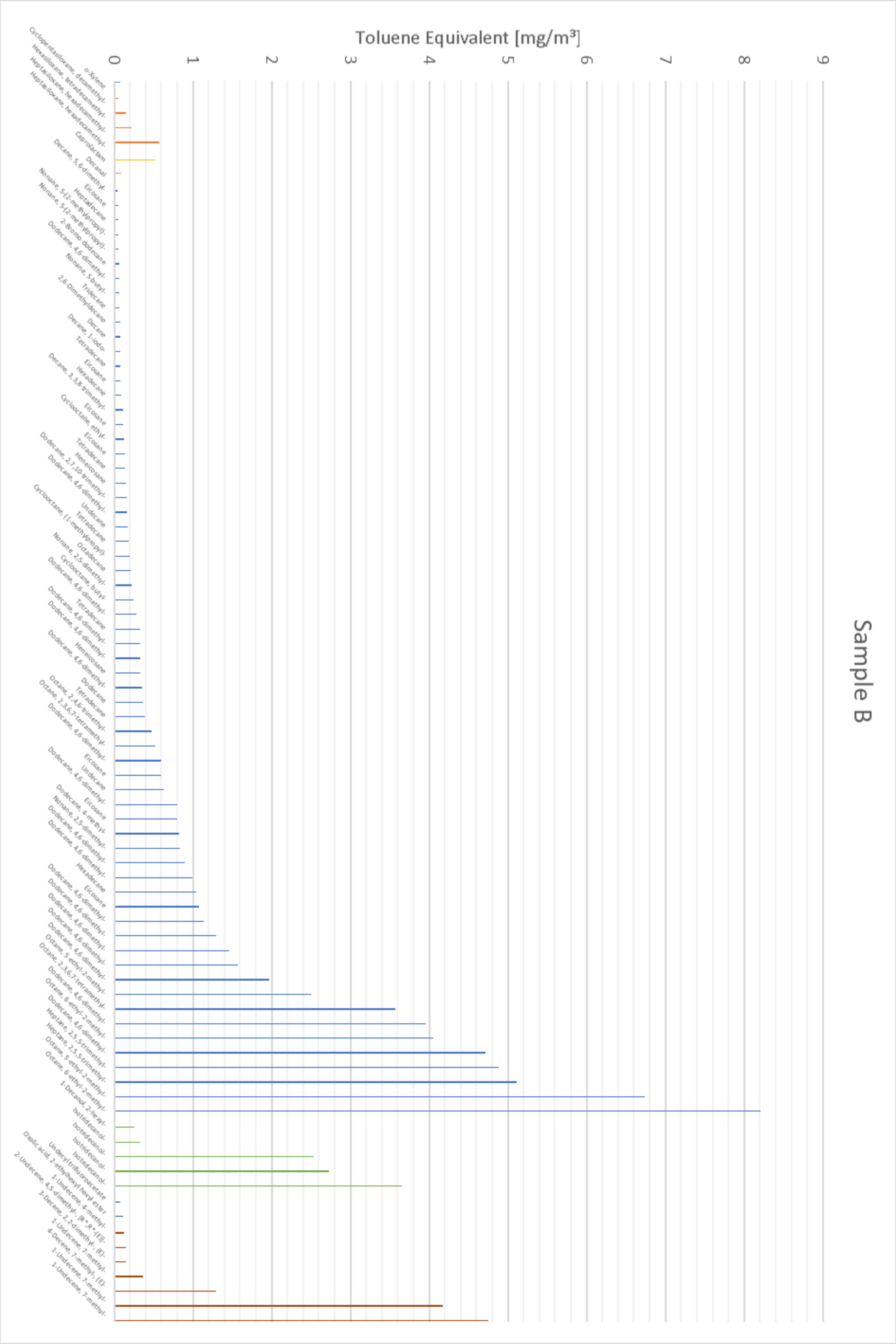

Figure S1 Toluene Equivalents [mg/m³] for all qualified analytes from Sample B

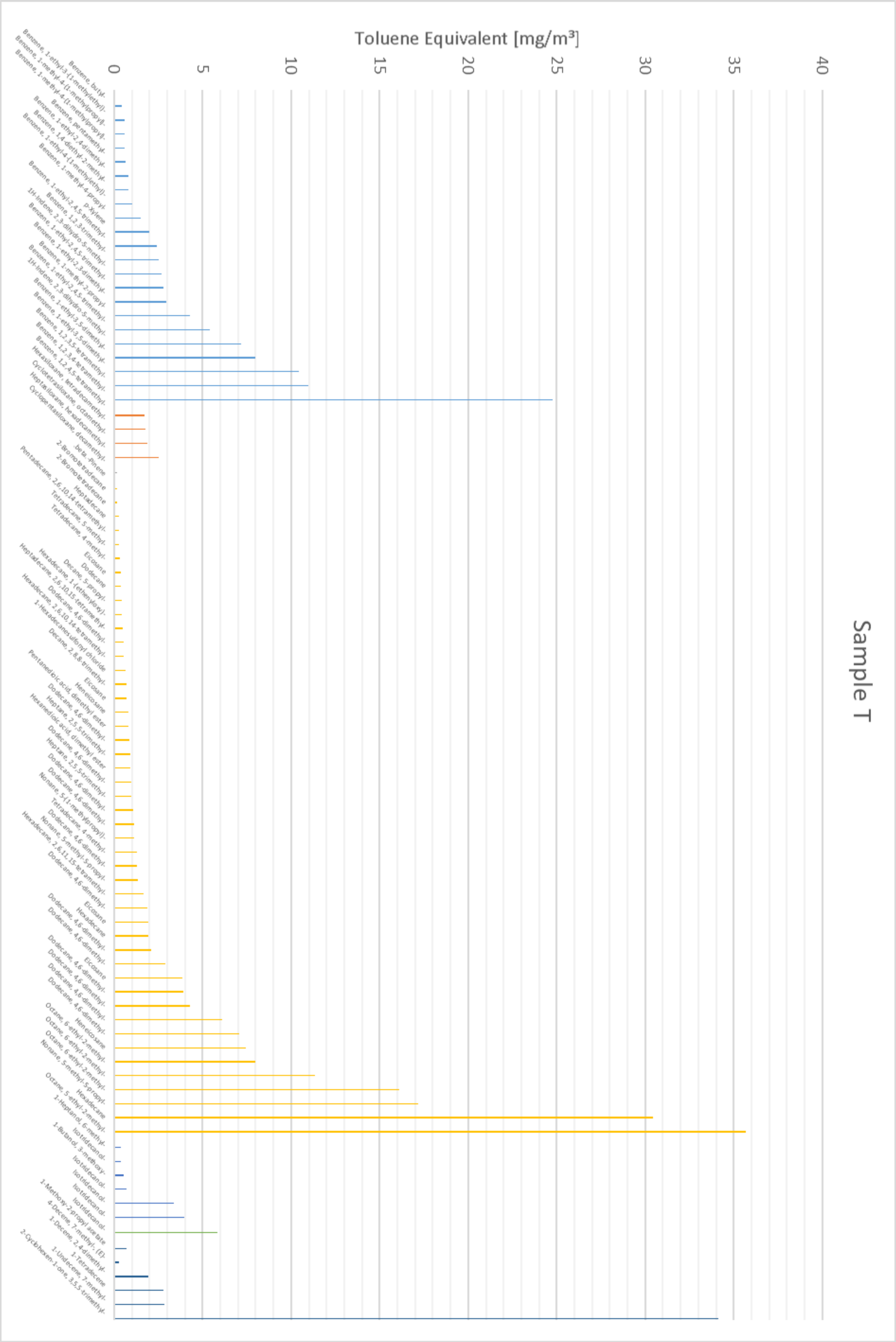

Figure S2 Toluene Equivalents [mg/m³] for all qualified analytes from Sample T

Table S3 Tabulated VOC values für Sample B, subdivided into substance classes with the corresponding LCI values

Substance / Class	Emission at 40°C [mg/m³]	LCI [mg/m³]	Comment
**Aromatic compounds**			
o-Xylene	0.07	0.5	
**Siloxanes**			
Cyclopentasiloxane, deca-methyl-	0.05	1.4	LCI for octamethyl-cyclotetrasilox-ane.
Hexasiloxane, tetradeca-methyl-	0.15		
Heptasiloxane, hexadeca-methyl-	0.23		
Heptasiloxane, hexadeca-methyl-	0.57		
**Amides**			
Caprolactam	0.53	0.3	
**Aldehydes**			
Decanal	0.09	0.9	
**Alkanes**			
Decane, 5,6-dimethyl-	0.04	14	other saturated C6-C8 hydrocar-bons
Nonane, 5-(2-methylpro-pyl)-	0.05	6	other saturated C9-C16 hydrocar-bons
Nonane, 5-(2-methylpro-pyl)-	0.05	6	other saturated C9-C16 hydrocar-bons
Dodecane, 4,6-dimethyl-	0.06	6	other saturated C9-C16 hydrocar-bons
Nonane, 5-butyl-	0.06	6	other saturated C9-C16 hydrocar-bons
Tridecane	0.06	6	other saturated C9-C16 hydrocar-bons
2,6-Dimethyldecane	0.07	6	other saturated C9-C16 hydrocar-bons
Decane	0.07	6	other saturated C9-C16 hydrocar-bons
Tetradecane	0.07	6	other saturated C9-C16 hydrocar-bons
Hexadecane	0.09	6	other saturated C9-C16 hydrocar-bons
Decane, 3,3,8-trimethyl-	0.11	6	other saturated C9-C16 hydrocar-bons
Cyclooctane, ethyl-	0.13	6	other saturated C9-C16 hydrocar-bons

EXCLI Journal 2021;20:995-1008 – ISSN 1611-2156
Received: April 08, 2021, accepted: May 26, 2021, published: June 01, 2021

Tetradecane	0.14	6	other saturated C9-C16 hydrocar-bons
Dodecane, 2,7,10-trime-thyl-	0.16	6	other saturated C9-C16 hydrocar-bons
Dodecane, 4,6-dimethyl-	0.16	6	other saturated C9-C16 hydrocar-bons
Undecane	0.17	6	other saturated C9-C16 hydrocar-bons
Tetradecane	0.18	6	other saturated C9-C16 hydrocar-bons
Cyclooctane, (1-methylpropyl)-	0.20	6	other saturated C9-C16 hydrocar-bons
Octadecane	0.21	6	other saturated C9-C16 hydrocar-bons
Nonane, 2,5-dimethyl-	0.22	6	other saturated C9-C16 hydrocar-bons
Cyclooctane, butyl-	0.24	6	other saturated C9-C16 hydrocar-bons
Dodecane, 4,6-dimethyl-	0.28	6	other saturated C9-C16 hydrocar-bons
Tetradecane	0.33	6	other saturated C9-C16 hydrocar-bons
Dodecane, 4,6-dimethyl-	0.34	6	other saturated C9-C16 hydrocar-bons
Dodecane, 4,6-dimethyl-	0.34	6	other saturated C9-C16 hydrocar-bons
Dodecane, 4,6-dimethyl-	0.35	6	other saturated C9-C16 hydrocar-bons
Dodecane	0.37	6	other saturated C9-C16 hydrocar-bons
Tetradecane	0.39	6	other saturated C9-C16 hydrocar-bons
Octane, 2,4,6-trimethyl-	0.47	6	other saturated C9-C16 hydrocar-bons
Octane, 2,3,6,7-tetrame-thyl-	0.52	6	other saturated C9-C16 hydrocar-bons
Dodecane, 4,6-dimethyl-	0.60	6	other saturated C9-C16 hydrocar-bons
Undecane	0.64	6	other saturated C9-C16 hydrocar-bons
Dodecane, 4,6-dimethyl-	0.80	6	other saturated C9-C16 hydrocar-bons
Dodecane, 4-methyl-	0.83	6	other saturated C9-C16 hydrocar-bons
Nonane, 2,5-dimethyl-	0.83	6	other saturated C9-C16 hydrocar-bons

EXCLI Journal 2021;20:995-1008 – ISSN 1611-2156
Received: April 08, 2021, accepted: May 26, 2021, published: June 01, 2021

Dodecane, 4,6-dimethyl-	0.90	6	other saturated C9-C16 hydrocarbons
Dodecane, 4,6-dimethyl-	1.00	6	other saturated C9-C16 hydrocarbons
Hexadecane	1.04	6	other saturated C9-C16 hydrocarbons
Dodecane, 4,6-dimethyl-	1.13	6	other saturated C9-C16 hydrocarbons
Dodecane, 4,6-dimethyl-	1.29	6	other saturated C9-C16 hydrocarbons
Dodecane, 4,6-dimethyl-	1.46	6	other saturated C9-C16 hydrocarbons
Dodecane, 4,6-dimethyl-	1.57	6	other saturated C9-C16 hydrocarbons
Dodecane, 4,6-dimethyl-	1.97	6	other saturated C9-C16 hydrocarbons
Octane, 5-ethyl-2-methyl-	2.50	6	other saturated C9-C16 hydrocarbons
Octane, 2,3,6,7-tetramethyl-	3.57	6	other saturated C9-C16 hydrocarbons
Dodecane, 4,6-dimethyl-	3.96	6	other saturated C9-C16 hydrocarbons
Octane, 6-ethyl-2-methyl-	4.06	6	other saturated C9-C16 hydrocarbons
Dodecane, 4,6-dimethyl-	4.72	6	other saturated C9-C16 hydrocarbons
Heptane, 2,5,5-trimethyl-	4.88	6	other saturated C9-C16 hydrocarbons
Heptane, 2,5,5-trimethyl-	5.12	6	other saturated C9-C16 hydrocarbons
Octane, 5-ethyl-2-methyl-	6.74	6	other saturated C9-C16 hydrocarbons
Octane, 6-ethyl-2-methyl-	8.21	6	other saturated C9-C16 hydrocarbons
Sum C9-C16 alkanes	*63.20*	6	other saturated C9-C16 hydrocarbons
Eicosane	1.08		
Eicosane	0.80		
Eicosane	0.60		
Eicosane	0.14		
Eicosane	0.11		
Eicosane	0.08		
Eicosane	0.05		
Heptadecane	0.05		
Heneicosane	0.34		

EXCLI Journal 2021;20:995-1008 – ISSN 1611-2156
Received: April 08, 2021, accepted: May 26, 2021, published: June 01, 2021

Heneicosane	0.15		
**Alcohols**			
1-Decanol, 2-hexyl-	0.25		
Isotridecanol-	0.34		
Isotridecanol-	2.53		
Isotridecanol-	2.73		
Isotridecanol-	3.66		
**Halogenated compounds**			
Decane, 1-iodo-	0.07		
Undecyl trifluoroacetate	0.07		
2-Bromo dodecane	0.06		
**Ester**			
Oxalic acid, 2-ethylhexyl hexyl ester	0.12		
**Alkenes**			
1-Undecene, 4-methyl-	0.13		
2-Undecene, 4,5-dimethyl-, [R*,R*-(E)]-	0.15		
3-Decene, 2,2-dimethyl-, (E)-	0.15		
1-Undecene, 7-methyl-	0.36		
4-Decene, 7-methyl-, (E)-	1.30		
1-Undecene, 7-methyl-	4.17		
1-Undecene, 7-methyl-	4.75		

EXCLI Journal 2021;20:995-1008 – ISSN 1611-2156
Received: April 08, 2021, accepted: May 26, 2021, published: June 01, 2021

Table S4 Tabulated VOC values für Sample T, subdivided into substance classes with the corresponding LCI values

Substance / Class	Emission at 40°C [mg/m³]	LCI [mg/m³]	Comment
**Aromatic compounds**			
Benzene, butyl-	0.07	1.1	
Benzene, 1-ethyl-3-(1-methylethyl)-	0.43		
Benzene, 1-methyl-4-(1-methylpropyl)-	0.58		
Benzene, 1-methyl-4-(1-methylpropyl)-	0.61		
Benzene, pentamethyl-	0.61		
Benzene, 1-ethyl-2,4-dimethyl-	0.65		
Benzene, 1,4-diethyl-2-methyl-	0.83		
Benzene, 1-ethyl-4-(1-methylethyl)-	0.83		
Benzene, 1-methyl-4-propyl-	1.01		
p-Xylene	1.52	0.5	
Benzene, 1-ethyl-2,4,5-trimethyl-	1.99		
Benzene, 1,2,3-trimethyl-	2.42		
Benzene, 1-ethyl-2,4,5-trimethyl-	2.67		
Benzene, 1-ethyl-2,3-dimethyl-	2.78		
Benzene, 1-methyl-2-propyl-	2.96		
Benzene, 1-ethyl-2,4,5-trimethyl-	4.30		
Benzene, 1-ethyl-3,5-dimethyl-	7.15		
Benzene, 1-ethyl-3,5-dimethyl-	7.98		
Benzene, 1,2,3,5-tetramethyl-	10.44	0.25	Tetramethylbenzene
Benzene, 1,2,3,4-tetramethyl-	10.95	0.25	Tetramethylbenzene
Benzene, 1,2,4,5-tetramethyl-	24.78	0.25	Tetramethylbenzene
**Siloxanes**			
Hexasiloxane, tetradecamethyl-	1.73		
Cyclotetrasiloxane, octamethyl-	1.77	1.2	
Heptasiloxane, hexadecamethyl-	1.88		

EXCLI Journal 2021;20:995-1008 – ISSN 1611-2156
Received: April 08, 2021, accepted: May 26, 2021, published: June 01, 2021

Cyclopentasiloxane, decame-thyl-	2.49		
**Alkanes**			
Tetradecane, 5-methyl-	0.29	6	other saturated C9-C16 hydrocar-bons
Tetradecane, 4-methyl-	0.33	6	other saturated C9-C16 hydrocar-bons
Dodecane	0.40	6	other saturated C9-C16 hydrocar-bons
Decane, 5-propyl-	0.43	6	other saturated C9-C16 hydrocar-bons
Dodecane, 4,6-dimethyl-	0.54	6	other saturated C9-C16 hydrocar-bons
Decane, 2,8,8-trimethyl-	0.69	6	other saturated C9-C16 hydrocar-bons
Dodecane, 4,6-dimethyl-	0.87	6	other saturated C9-C16 hydrocar-bons
Heptane, 2,5,5-trimethyl-	0.90	6	other saturated C9-C16 hydrocar-bons
Dodecane, 4,6-dimethyl-	0.98	6	other saturated C9-C16 hydrocar-bons
Heptane, 2,5,5-trimethyl-	0.98	6	other saturated C9-C16 hydrocar-bons
Dodecane, 4,6-dimethyl-	1.05	6	other saturated C9-C16 hydrocar-bons
Dodecane, 4,6-dimethyl-	1.12	6	other saturated C9-C16 hydrocar-bons
Nonane, 5-(1-methylpropyl)-	1.12	6	other saturated C9-C16 hydrocar-bons
Tetradecane, 4-methyl-	1.26	6	other saturated C9-C16 hydrocar-bons
Dodecane, 4,6-dimethyl-	1.30	6	other saturated C9-C16 hydrocar-bons
Nonane, 5-methyl-5-propyl-	1.34	6	other saturated C9-C16 hydrocar-bons
Dodecane, 4,6-dimethyl-	1.88	6	other saturated C9-C16 hydrocar-bons
Hexadecane	1.95	6	other saturated C9-C16 hydrocar-bons
Dodecane, 4,6-dimethyl-	2.10	6	other saturated C9-C16 hydrocar-bons
Dodecane, 4,6-dimethyl-	2.89	6	other saturated C9-C16 hydrocar-bons
Dodecane, 4,6-dimethyl-	3.90	6	other saturated C9-C16 hydrocar-bons

EXCLI Journal 2021;20:995-1008 – ISSN 1611-2156
Received: April 08, 2021, accepted: May 26, 2021, published: June 01, 2021

Dodecane, 4,6-dimethyl-	4.30	6	other saturated C9-C16 hydrocar-bons
Dodecane, 4,6-dimethyl-	6.11	6	other saturated C9-C16 hydrocar-bons
Dodecane, 4,6-dimethyl-	7.04	6	other saturated C9-C16 hydrocar-bons
Octane, 6-ethyl-2-methyl-	7.98	6	other saturated C9-C16 hydrocar-bons
Octane, 6-ethyl-2-methyl-	11.34	6	other saturated C9-C16 hydrocar-bons
Octane, 6-ethyl-2-methyl-	16.11	6	other saturated C9-C16 hydrocar-bons
Nonane, 5-methyl-5-propyl-	17.20	6	other saturated C9-C16 hydrocar-bons
Hexadecane	30.42	6	other saturated C9-C16 hydrocar-bons
Octane, 5-ethyl-2-methyl-	35.69	6	other saturated C9-C16 hydrocar-bons
Sum of C9-C16-alkanes	*162.50*	6	other saturated C9-C16 hydrocar-bons
Eicosane	3.87		
Eicosane	1.91		
Hexadecane, 2,6,11,15-tetra-methyl-	1.66		
Heneicosane	7.44		
Eicosane	0.69		
Heneicosane	0.79		
Eicosane	0.36		
Heptadecane	0.29		
Pentadecane, 2,6,10,14-tetra-methyl-	0.29		
Heptadecane, 2,6,10,15-tetra-methyl-	0.51		
Hexadecane, 2,6,10,14-tetra-methyl-	0.54		
**Alcohols**			
1-Heptanol, 6-methyl-	0.36		
Isotridecanol-	0.36		
1-Butanol, 3-methoxy-	0.54		
Isotridecanol-	0.72		
Isotridecanol-	3.40		
Isotridecanol-	3.97		
Isotridecanol-	5.85		
**Esters**			
1-Methoxy-2-propyl acetate	0.72	0.65	

EXCLI Journal 2021;20:995-1008 – ISSN 1611-2156
Received: April 08, 2021, accepted: May 26, 2021, published: June 01, 2021

Hexanedioic acid, dimethyl ester	0.90	0.05	
Pentanedioic acid, dimethyl ester	0.79	0.025	
**Alkenes**			
4-Decene, 7-methyl-, (E)-	0.29		
1-Decene, 2,4-dimethyl-	1.95		
1-Tetradecene	2.78		
1-Undecene, 7-methyl-	2.82		
2-Cyclohexen-1-one, 3,5,5-trimethyl-	34.14		
1H-Indene, 2,3-dihydro-5-methyl-	2.49		
1H-Indene, 2,3-dihydro-5-methyl-	5.42		
Hexadecane, 1-(ethenyloxy)-	0.43		
.beta.-Pinene	0.14		
**Halogenated Compounds**			
1-Hexadecanesulfonyl chloride	0.65		
2-Bromotetradecane	0.14		
2-Bromotetradecane	0.14		
